# Covariance decomposition for distance based species tree estimation

**DOI:** 10.1186/s12859-026-06495-z

**Published:** 2026-07-04

**Authors:** Georgios Aliatimis, Ruriko Yoshida, Burak Boyacı, James A. Grant

**Affiliations:** 1 STOR-i Centre for Doctoral Training, Lancaster University, Lancaster, LA1 4YW UK; 2 Department of Operations Research, Naval Postgraduate School, 1411 Cunningham Road, Monterey, CA 93943 USA; 3 Management School, Lancaster University, Lancaster, LA1 4YX UK; 4 School of Mathematical Sciences, Lancaster University, Lancaster, LA1 4YF UK

**Keywords:** METAL, Multispecies coalescent model, Substitution models, Covariance, Split support

## Abstract

In phylogenomics, species-tree methods must contend with two major sources of noise; stochastic gene-tree variation under the multispecies coalescent model (MSC) and finite-sequence substitutional noise. Fast agglomerative methods such as GLASS, STEAC, and METAL combine multi-locus information via distance-based clustering. We derive the exact covariance matrix of these pairwise distance estimates under a joint MSC-plus-substitution model and leverage it for reliable confidence estimation, and we algebraically decompose it into components attributable to coalescent variation versus sequence-level stochasticity. Our theory identifies parameter regimes where one source of variance greatly exceeds the other. For both very low and very high mutation rates, substitutional noise dominates, while coalescent variance is the primary contributor at intermediate mutation rates. Moreover, the interval over which coalescent variance dominates becomes narrower as the species-tree height increases. These results imply that in some settings one may legitimately ignore the weaker noise source when designing methods or collecting data. In particular, when gene-tree variance is dominant, adding more loci is most beneficial, while when substitution noise dominates, longer sequences or imputation are needed. Finally, leveraging the derived covariance matrix, we implement a Gaussian-sampling procedure to generate split support values for METAL trees and demonstrate empirically that this approach yields more reliable confidence estimates than traditional bootstrapping.

## Introduction

A core problem in phylogenomics is the reconstruction of a species tree from gene alignments. A species tree encodes the evolutionary relationships and divergence times among given species, while gene trees represent the evolutionary histories of individual genes. The structure of a gene tree may differ from the species tree due to variation induced by processes such as incomplete lineage sorting (ILS), horizontal gene transfer and hybridization [1]. Finally, gene alignments are comparisons of DNA, RNA, or protein sequences across different species, from which gene trees may inferred. The task of species tree reconstruction is to infer the species tree from gene alignments.

**Fig. 1:**
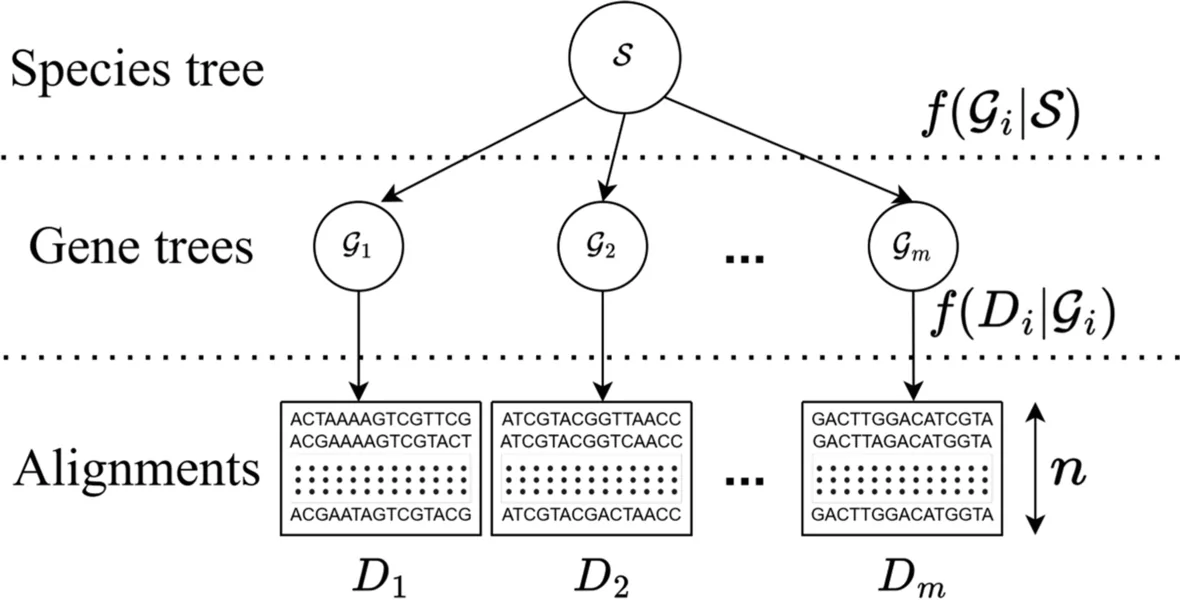
In phylogenetics, the goal is to reconstruct gene trees $G_i$ for *i ∈ [m]* (intermediate level) from *m* gene alignments (bottom level) through a nucleotide substitution model *F(D|G)*. In phylogenomics, the aim is to infer the species tree $\mathcal {S}$ (top level) from which these gene trees arose through the MSC model $f(G|\mathcal {S})$

Hence, species tree reconstruction is commonly formulated as a hierarchical statistical model with three levels, as illustrated in Fig. 1. At the top level, the species tree $\mathcal {S}$ is the parameter of ultimate interest, to be estimated either as a point estimate or via a (approximate) posterior distribution. Given $\mathcal {S}$, the second level consists of *m* gene trees $\{G_i : i \in [m]\}$, whose likelihoods $f(G_i \mid \mathcal {S})$ are defined by the multispecies coalescent (MSC) model, which captures the stochasticity introduced by incomplete lineage sorting (ILS) under a given species tree [2]. At the bottom level are the gene alignments $\{D_i : i \in [m]\}$, whose likelihoods $f(D_i \mid G_i)$ depend only on the corresponding gene trees, and are computed using nucleotide substitution models such as the Jukes-Cantor (JC69) model [3], the Hasegawa-Kishino-Yano (HKY) model [4], and the Generalized Time Reversible (GTR) model [5]. In practice, we observe only the gene alignments. Depending on the method, one may first infer the gene trees $\{G_i: i\in [m]\}$ and then the species tree, or infer the species tree $\mathcal {S}$ directly from sequence data without explicit gene-tree reconstruction. While the present work focuses on the Jukes-Cantor model for simplicity and analytical tractability, more general distance corrections may be considered when substitution rates deviate from the assumptions of Jukes-Cantor model (e.g., unequal base frequencies or transition–transversion biases). In such cases, alternatives such as logdet distances [6], which enjoy desirable properties under heterogeneous substitution processes, may provide less biased distance estimates.

Viewing a species tree reconstruction via a hierarchical model has several conceptual advantages: it clarifies the distinct sources of randomness (under coalescent and substitution models), provides a foundation for Bayesian approaches [7], and facilitates the development of statistical methods for quantifying uncertainty at each level. Moreover, it enables the integration of multiple data types and model components within a coherent inferential framework, as seen in widely used tools such as MrBayes [8], BEAST [9], and BPP [10].

### Species tree reconstruction methods

In contrast to computationally intensive methods for species tree reconstruction, distance-based approaches based on summary statistics—such as METAL [11], GLASS [12], and STEAC [13]—offer simpler and more computationally efficient alternatives. These methods rely on estimated coalescent times or Hamming distances between species pairs, and then define summary statistics over these estimates across all genes. GLASS, which uses the *minimum* coalescent times across genes as its summary statistic, can provide more accurate species tree estimates than METAL or STEAC when gene tree uncertainty is minimal. In contrast, METAL and STEAC, which rely on *averages* as their summary measure, tend to be more robust in the presence of greater variance in gene tree estimates or forms of gene tree heterogeneity other than ILS.

Both STEAC and GLASS were originally developed as methods that take gene trees as input and produce an inferred species tree as output. Their application in phylogenomics thus depends on the accurate reconstruction of gene trees from gene sequence data. In contrast, METAL was specifically designed to infer species trees directly from gene sequences without requiring intermediate gene-tree estimation. As a result, METAL typically achieves accurate species tree reconstruction with fewer loci than methods that rely on gene-tree estimation, such as GLASS and STEAC. Notably, when working directly with sequence data, METAL requires only $\mathcal {O}(\tau ^{-2})$ total sites to recover the correct species tree topology with a a specified high probability, where *τ * is the shortest internal branch length in the species tree. By comparison, GLASS and STEAC require $\mathcal {O}(\tau ^{-3})$ and $\mathcal {O}(\tau ^{-4})$ total sites respectively [11]. However, GLASS can outperform METAL in regimes when the number of bases per gene is large; as substitutional noise diminishes, GLASS can yield more accurate estimates than METAL due to its minimum distance criterion.

Despite the computational efficiency and practical appeal of distance-based methods, their behavior under different sources of uncertainty has remained only partially understood. Prior work has noted limitations of early approaches such as GLASS and proposed refinements (e.g., iGLASS [14]) to mitigate sensitivity to mutational noise. Other studies have explicitly examined the impact of gene-tree variance and substitutional error on inference accuracy, including simulation-based analyses that teased apart their relative contributions [15]. What remains less clear, however, is how these two sources of uncertainty–coalescent variation versus substitutional noise–interact across parameter regimes, and under which conditions methods such as STEAC, METAL, or more recent variants of GLASS will be most reliable. The relative influence of these error sources can shift dramatically with mutation rate, sequence length, and tree height, making a systematic understanding essential.

Although bootstrap resampling of concatenated sequences is common for assessing split support, it conflates coalescent and substitutional uncertainties and can yield misleading confidence values under extreme variance regimes. By deriving the full covariance matrix of METAL’s pairwise distances, we introduce a Gaussian-sampling procedure that produces more accurate split support estimates than traditional bootstrapping.

### Contributions

Considering the hierarchical nature of species tree reconstruction, uncertainty arises from two distinct sources; (1) variation in tree topologies governed by MSC, and (2) sequence-level stochasticity introduced by the substitution process used to infer gene trees from alignments (e.g., as JC69). By explicitly deriving the covariance matrices corresponding to each source, we obtain a quantitative framework for understanding how uncertainty propagates through distance-based estimates. Related work by Guerra and Nielsen [16] has also derived the covariance of pairwise distances under the multispecies coalescent with mutation, in a more general setting that accommodates arbitrary species trees and varying ancestral population sizes.

Our analysis differs in focusing specifically on distance-based species tree reconstruction methods (METAL, GLASS, STEAC), and in decomposing the total covariance into coalescent and substitutional components to study how their relative magnitudes govern method performance. In particular, we demonstrate that METAL outperforms GLASS when substitutional variance dominates the total uncertainty, and vice versa - this finding supports practitioners in using intuition about the prominent sources of uncertainty to select a method. Furthermore, we find that METAL outperforms STEAC in all of our experiments.

This decomposition enables informed decisions about study design, such as whether to prioritize collecting longer sequences or more independent loci. When coalescent variance dominates, increasing the number of loci is most effective; when substitutional noise is dominant, longer alignments (perhaps via imputation strategies) are preferable.

In Sect. “Species tree reconstruction from gene trees”, we analyze species tree reconstruction from known gene trees under METAL, GLASS, and STEAC. We compute the covariance structure of METAL and STEAC, and show that METAL effectively interpolates between STEAC and GLASS as a function of mutation rate. In Sect. “Gene tree reconstruction from base sequences”, we study species tree reconstruction from gene base sequences, analyzing the statistical properties (signal and noise) of the distance estimates used by METAL and STEAC under finite sequence lengths. Section “Theoretical results” presents our theoretical results: the decomposition of the total covariance of METAL and STEAC into coalescent and substitutional components, analysis of the spectrum of the METAL covariance matrix, and asymptotic expressions quantifying their relative magnitudes across a range of model parameters. Section “Simulation results” contains two empirical applications. Section “Species-tree estimation” compares the performance of METAL, GLASS, and STEAC across regimes where substitutional or coalescent variance dominates, showing that the relative error of GLASS versus METAL depends critically on the proportion of variance attributable to substitutional noise. In Sect. “Bipartition confidence”, we introduce a Gaussian-sampling procedure for computing support values for METAL trees, enabled by our covariance derivations. We show empirically that this method provides more reliable split confidence than traditional bootstrapping. Finally, Sect. “Discussion” concludes the paper with a discussion of the implications, limitations, and future directions of this work.

## Species tree reconstruction from gene trees

First, we introduce the notation that will be used in the rest of the paper. The task is to successfully reconstruct the species tree *S = (V,E)*, where *V* and *E* is the set of nodes and edges in the tree respectively. The leaf set *L ⊆ V* corresponds to the *n=|L|* extant species examined. We assume throughout that the species tree is ultrametric, consistent with a molecular clock model in which all lineages evolve at the same rate, so that all root-to-leaf path lengths are equal. We define $\tau _e$ to be the length of *e ∈ E* in coalescent units and the diameter of the tree as $\Delta = \max _{a,b \in L} \tau _{ab}$.

For simplicity, we sample a single individual per species, following the approach implemented in agglomerative, distance-based frameworks such as METAL [17]. We consider *m* loci with associated gene trees $G^{(i)}, i \in [m]$ with the same leaf set *L*, and define $g_{ab}^{(i)}$ as twice the time in coalescent units to the most recent ancestor of *a* and *b* in $G^{(i)}$. This is the evolutionary distance between the two leaves. In this section, we assume that the gene trees are known. Each of the three methods examined in this paper uses a dissimilarity map $d: L^2 \rightarrow \mathbb {R}_ +$ and subsequently performs hierarchical clustering on that distance matrix. The resulting dendrogram is an estimate of the species tree. Pseudocode for these algorithms can be found in Appendix C, Algorithm 2.

The dissimilarity maps are defined as follows:

1 $$\begin{aligned} d^\mathrm{(GLASS)}(a,b)&= \min _{i \in [m]} g_{ab}^{(i)}, \end{aligned}$$

2 $$\begin{aligned} d^\mathrm{(STEAC)}(a,b)&= \frac{1}{m} \sum _{i=1}^{m} g_{ab}^{(i)}, \end{aligned}$$

3 $$\begin{aligned} d^\mathrm{(METAL)}(a,b)&= \frac{1}{m} \sum _{i=1}^{m} p_{ab}^{(i)} = \frac{3}{4}\left( 1 - \frac{1}{m} \sum _{i=1}^{m} \exp \left( - \mu g_{ab}^{(i)} \right) \right) , \end{aligned}$$

for all pairs $$(a,b) \in \left( {\begin{array}{c}L\\ 2\end{array}}\right) $$ where $p_{ab}^{(i)}$ is the probability that the bases of species *a* and *b* differ at any given site in locus *i* under the Jukes-Cantor evolutionary model, and *μ * is rate of substitution.

Note that METAL does not require prior knowledge of the mutation rate *μ * when applied to empirical sequence alignments. In practice, one simply computes the normalized Hamming distance $\hat{p}_{ab}^{(i)}$ between alignments of species *a* and *b* for each locus *i*, which is the proportion of sites that differ between those two alignments, and then averages over loci to obtain

$$ \hat{d}^\mathrm{(METAL)}(a,b) = \frac{1}{m} \sum _{i=1}^{m} \hat{p}_{ab}^{(i)}. $$

As the number of sites per locus tends to infinity, $\hat{p}_{ab}^{(i)} \rightarrow p_{ab}^{(i)}$, and thus $\hat{d}^\mathrm{(METAL)}(a,b) \rightarrow d^\mathrm{(METAL)}(a,b)$. Under the Jukes–Cantor model [3], the *p*-distance $p_{ab}$ is related to the Jukes-Cantor distance $d_\textrm{JC}(a,b)$, which is measured in expected number of substitution time units, through

$$ p_{ab } = \frac{3}{4} \left( 1- \exp \left( -\frac{4}{3} d_\textrm{JC}(a,b) \right) \right) , $$

which, yields Eq. (3) by noting that $d_\textrm{JC}(a,b) = \frac{3}{4} \mu g_{ab}$.

Proposition 1 shows that, under perfect gene-tree knowledge, the METAL distance “interpolates” between STEAC and GLASS: as the substitution rate $\mu \rightarrow 0$, it reduces to STEAC’s average coalescent time, whereas as $\mu \rightarrow \infty$, it collapses to GLASS’s minimum-coalescent-time criterion.

### **Proposition 1**

*Let* $\hat{T}_\textrm{METAL}(\mu ), \hat{T}_\textrm{STEAC}, \hat{T}_\textrm{GLASS}$* be the reconstructed species tree topologies of each method provided that the correct gene trees are given, assuming that all methods use the same hierarchical clustering method, and that all the minima attained by GLASS are unique. Then,*

$$\begin{aligned} \lim _{\mu \rightarrow 0} \hat{T}_\textrm{METAL}(\mu )&= \hat{T}_\textrm{STEAC}, \\ \lim _{\mu \rightarrow \infty } \hat{T}_\textrm{METAL}(\mu )&= \hat{T}_\textrm{GLASS}. \end{aligned}$$

*For the second limit, it is assumed that the hierarchical clustering method is single or complete linkage clustering.*

### *Proof*

All proofs can be found in Appendix A. *□ *

Proposition 2 then establishes that, for any four leaves *a,b,c,d∈ L*, the pairwise distances produced by STEAC and by METAL are both nonnegatively correlated, with STEAC correlations always at least as large as those of METAL. The covariance matrix of METAL pairwise distances–assembled from the individual covariance components derived in the proof of Proposition 2–will be used for uncertainty quantification in Sect. “Theoretical results” and to formulate the total covariance in our simulation results (Sect. “Simulation results”).

### **Proposition 2**

*Let* *a,b,c,d ∈ L* *be leaves in the species tree. Then, under MSC,*

$$\begin{aligned} \textrm{Cor}\left( g_{ab}^{(1)}, g_{cd}^{(1)} \right) \ge \textrm{Cor}\left( e^{t g_{ab}^{(1)}}, e^{t g_{cd}^{(1)}} \right) \ge 0, \forall t\le 0, \end{aligned}$$

*where* $\textrm{Cor}$ *denotes the correlation between two random variables. Moreover,* $\textrm{Cor}\left( e^{t g_{ab}^{(1)}}, e^{t g_{cd}^{(1)}} \right)$ *as a function of* *t* *is continuous and strictly increasing on* *(-∞ ,0)* *with limit*

$$\begin{aligned} \lim _{t \rightarrow 0} \textrm{Cor}\left( e^{t g_{ab}^{(1)}}, e^{t g_{cd}^{(1)}} \right)&= \textrm{Cor}\left( g_{ab}^{(1)}, g_{cd}^{(1)} \right) . \\ \end{aligned}$$

*Finally,*

$$\begin{aligned} \textrm{Cor}\left( d^\mathrm{(STEAC)}(a,b), d^\mathrm{(STEAC)}(c,d) \right)&= \textrm{Cor}\left( g_{ab}^{(1)}, g_{cd}^{(1)} \right) \\ \textrm{Cor}\left( d^\mathrm{(METAL)}(a,b), d^\mathrm{(METAL)}(c,d) \right)&= \textrm{Cor}\left( e^{t g_{ab}^{(1)}}, e^{t g_{cd}^{(1)}} \right) \end{aligned}$$

## Gene tree reconstruction from base sequences

In the previous section, we considered properties of the distribution of gene trees $G_i | S$ under the MSC model. In this section, we investigate statistical properties of gene base sequence conditional on the gene tree or $\chi ^{i}| G_i$. Let $\chi _{a}^{ij} \in \{A,C,T,G\}$ be the $j^\textrm{th}$ base of the $i^\textrm{th}$ locus of species *a ∈ L*, where *i ∈ [m]*, $j \in [K_i]$, *m* is the number of loci studied and $K_i$ is the sequence length of loci *i*. For notational simplicity in the formulae that follow, we assume that all loci have the same number of bases $K = K_i, ~ \forall i \in [m]$. The analysis can be readily extended to the case of loci with varying sequence lengths. The normalized Hamming distance, $\hat{p}^{(i)}_{ab}$ and Jukes-Cantor distance $\hat{\nu }_{ab}^{(i)}$ between species *a, b∈ L* are then defined as

$$\begin{aligned} \hat{p}_{ab}^{(i)}&= \frac{1}{K} \sum _{j=1}^K 1\!\!1\left( \chi _{a}^{ij} \ne \chi _{b}^{ij} \right) , \text { and}\\ \hat{\nu }_{ab}^{(i)}&= \frac{3}{4}\mu \hat{g}_{ab}^{(i)} = - \frac{3}{4} \log \left( 1 - \frac{4}{3} \hat{p}_{ab}^{(i)} \right) . \end{aligned}$$

METAL computes the average of the normalized Hamming distances, while STEAC and GLASS consider the average and minimum Jukes-Cantor distances respectively. However, a problem with using the Jukes-Cantor distances directly is that they are infinite if $\hat{p}_{ab}^{(i)} \ge \frac{3}{4}$ for any *a,b ∈ L*, *i ∈ [m]*, which has a non-zero chance of occurring.

### Hamming distances

Given the gene tree $G^{(i)}$ of locus *i*, the distribution of $\hat{p}^{(i)}_{ab}$ is

$$\begin{aligned} \hat{p}_{ab}^{(i)} = \frac{1}{K} \sum _{j=1}^K 1\!\!1\left( \chi _{a}^{ij} \ne \chi _{b}^{ij} \right)&\sim { \frac{1}{K}} \textrm{Bin}\left( K, p_{ab}^{(i)}\right) , \text { where}\\ p_{ab}^{(i)}&= \frac{3}{4}\left( 1 - \exp \left( -\mu g_{ab}^{(i)} \right) \right) . \end{aligned}$$

Effectively, $p_{ab}^{(i)}$ is the true probability that species *a* and *b* would share the same base in locus *i* and $\hat{p}_{ab}^{(i)}$. The number of bases, *K*, is typically large and so we can apply the common Normal approximation of the Binomial distribution,

$$\begin{aligned} \hat{p}_{ab}^{(i)}\sim \mathcal {N}\left( p_{ab}^{(i)}, \frac{p_{ab}^{(i)} \left( 1-p_{ab}^{(i)}\right) }{K}\right) . \end{aligned}$$

**Fig. 2:**
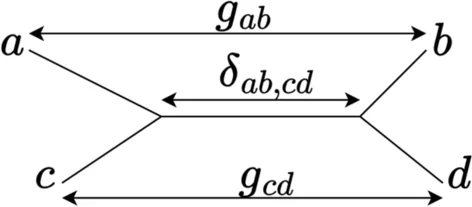
The evolutionary tree used in proposition 3. Here $\delta _{ab,cd} = \delta _{ac,bd}$ is the length of the intersection of the shortest path from *a* to *b* and the shortest path from *c* to *d*. All trees with 4 distinct leaves *a,b,c,d ∈ L* can be drawn in this way. For cherry trees, the pairs (*a*, *c*), (*b*, *d*) are sisters/cherries and the root of the 4-leaf tree is contained in the *δ *-segment. For comb trees, without loss of generality, the topology is (( (*a*, *c*), *b*), *d*) with *a*, *c* being sisters and the root of the tree contained in the shortest path from *b* to *d*. Note that since the paths from *a* to *c* and *b*, *d* are disconnected $\delta _{ac,bd} =0$

While the normal approximation above applies to each pairwise distance $\hat{p}_{ab}^{(i)}$ individually, we require a joint multivariate description of all $$\left( {\begin{array}{c}L\\ 2\end{array}}\right) $$ distances at locus *i*. In fact, the multivariate extension of that result is

$$\hat{p}_{\left( {\begin{array}{c}L\\ 2\end{array}}\right) }^{(i)} \;\sim \; \mathcal {N}\left(p_{\left( {\begin{array}{c}L\\ 2\end{array}}\right) }^{(i)},\,\tfrac{1}{K}\,\Sigma ^{(i)}\right), $$

where $$\hat{p}_{\left( {\begin{array}{c}L\\ 2\end{array}}\right) }^{(i)},\,p_{\left( {\begin{array}{c}L\\ 2\end{array}}\right) }^{(i)}\in \mathbb {R}^{\left( {\begin{array}{c}n\\ 2\end{array}}\right) }$$ are vectors containing the estimated and true pairwise distances respectively and $$\Sigma ^{(i)}\in \mathbb {R}^{\left( {\begin{array}{c}n\\ 2\end{array}}\right) \times \left( {\begin{array}{c}n\\ 2\end{array}}\right) }$$ is the covariance matrix whose entries are of the form given by Proposition 3.

#### **Proposition 3**

*Let* *a,b,c,d ∈ L* *be leaves of a gene tree, where the pairs* *a*, *c* and *b*, *d are sisters as shown in Fig. *2. *Letting* $\delta _{ab,cd}$ *be the length of the intersection of the shortest path from* *a to b* *the shortest path from c to d, the covariance is*

4 $$\begin{aligned} \textrm{Cov}\left( 1\!\!1\left( \chi _a^{(i)} \ne \chi _b^{(i)} \right) , 1\!\!1\left( \chi _c^{(i)} \ne \chi _d^{(i)} \right) | G \right) = \frac{3}{16} e^{-\mu \left( g_{ab}^{(i)} + g_{cd}^{(i)} \right) } \left( e^{2\mu \delta _{ab,cd}^{(i)}} + 2 e^{\mu \delta _{ab,cd}^{(i)}} - 3 \right) \end{aligned}$$

Using Proposition 3, we see in particular, that the diagonal entries of $\Sigma ^{(i)}$ coincide with the univariate variances: by expanding Eq. (4) and noting that $\delta _{ab,ab}=g_{ab}$, we have,

$$ {{\,\textrm{Var}\,}}\bigl (1\!\!1(\chi _a^{(i)}\ne \chi _b^{(i)}) | G\bigr )\;=\; p_{ab}^{(i)}\left( 1-p_{ab}^{(i)}\right) \,. $$

### Jukes-Cantor distances

For the STEAC method, instead of using normalized Hamming distances, $\hat{p}_{ab}$, it uses estimates for the gene tree branch lengths

5 $$\begin{aligned} \hat{g}_{ab} = - \frac{1}{\mu } \log \left( 1- \frac{4}{3} \hat{p}_{ab} \right) , \forall a,b \in L. \end{aligned}$$

The major issue with this approach is that if at any loci *i ∈ [m]* we get $\hat{p}_{ab}^{(i)} \ge 3/4$, then $\hat{g}_{ab}^{(i)} = \infty$ and so $\hat{d}_{ab}^\mathrm{(STEAC)} = \infty$. Clearly, this is undesirable, especially when the number of genes is large and the number of sites per gene *K* is low, when such events are most common. Hierarchical clustering methods such as UPGMA would not allow clusters containing leaves *a* and *b* to ever merge.

In this paper we consider the case when *K ≫ 1*, when the Delta method is typically used to approximate the distribution of $\hat{g}_{ab}$ in Eq. (5), which dictates that

$$\begin{aligned} \hat{g}_{\left( {\begin{array}{c}L\\ 2\end{array}}\right) }^{(i)}&\sim \mathcal {N}\left( g_{\left( {\begin{array}{c}L\\ 2\end{array}}\right) }^{(i)}, \Sigma _\textrm{STEAC}^{(i)} \right) , \end{aligned}$$

where $$\hat{g}_{\left( {\begin{array}{c}L\\ 2\end{array}}\right) }^{(i)}$$, $${g}_{\left( {\begin{array}{c}L\\ 2\end{array}}\right) }^{(i)} \in \mathbb {R}^{\left( {\begin{array}{c}n\\ 2\end{array}}\right) }$$ are vectors containing the estimated and true Jukes-Cantor distances respectively, and the row *ab*, column *cd* element of the covariance matrix $\Sigma _\textrm{STEAC}^{(i)}$ is

6 $$\begin{aligned}&\textrm{Cov}\left( \hat{g}_{ab}^{(i)}, \hat{g}_{cd}^{(i)} | G\right) \nonumber \\&\approx \frac{ \textrm{Cov}\left( 1\!\!1\left( \chi _a^{(i)} \ne \chi _b^{(i)} \right) , 1\!\!1\left( \chi _c^{(i)} \ne \chi _d^{(i)} \right) \right) }{K \mu ^2 \left( \frac{3}{4} - p_{ab}^{(i)} \right) \left( \frac{3}{4} - p_{cd}^{(i)} \right) }\nonumber \\&=\frac{e^{2\mu \delta _{ab,cd}^{(i)}} + 2e^{\mu \delta _{ab,cd}^{(i)}} - 3}{3\mu ^2 K}, \end{aligned}$$

where the last equality follows from Proposition 3 and the definition of $p_{ab}^{(i)}$. In the following section, we make use of these derived covariance matrices to compute the total covariance matrix of the METAL and STEAC pair distances.

## Theoretical results

In this section, we analyze both the *signal* and *noise* of distance-based inference under the MSC+Jukes–Cantor model. The signal is quantified by the *expected gap* in the *four-point condition* for quartets, which reflects the intrinsic separation of sister pairs in the species tree. Intuitively, the greater the expected gap, the easier it is to correctly reconstruct the quartet. The noise of METAL and STEAC estimators is captured by their total covariance which can be decomposed into coalescent and substitution components, thereby quantifying the relative contributions of coalescent/genealogical and substitution/mutational uncertainty. The spectral properties of these matrices are also analyzed.

### Expected gap

The following Proposition formalizes the expected four-point gap for a quartet of leaves.

#### **Proposition 4**

*Let* $\{a,b,c,d\}$ *be four leaves with* *a* *and* *b* *forming a sister pair (i.e.* $$ S_{ab} = \min _{p \in \left( {\begin{array}{c}\{a,b,c,d\}\\ 2\end{array}}\right) } S_p $$*). Then the expected four-point gap is*

7 $$\begin{aligned} \mathbb {E}\!\left( \hat{d}_{ad} + \hat{d}_{bc} - \hat{d}_{ab} - \hat{d}_{cd} \right) = \frac{1}{1+2\mu } \left( e^{-\mu S_{ab}} + e^{-\mu S_{cd}} - e^{-\mu S_{ad}} - e^{-\mu S_{bc}} \right) , \end{aligned}$$

*where* $\hat{d}$ *is the pairwise distances of the METAL estimator. If the tree has cherry topology* *((a,b),(c,d))* *with quartet diameter* $\Delta _{\{a,b,c,d\}} = S_{ad} = S_{bc}$*, Eq. (*7*) simplifies to*

$$ \mathbb {E}\!\left( \hat{d}_{ad} + \hat{d}_{bc} - \hat{d}_{ab} - \hat{d}_{cd} \right) = \left( e^{-\mu S_{ab}} + e^{-\mu S_{cd}} - 2 e^{-\mu \Delta _{\{a,b,c,d\}}} \right) /(1+2\mu ). $$

*For a comb topology, w.l.o.g. taking the topology* *(( (a,b),c),d)**, the expression reduces to*

$$ \mathbb {E}\!\left( \hat{d}_{ad} + \hat{d}_{bc} - \hat{d}_{ab} - \hat{d}_{cd} \right) = \big ( e^{-\mu S_{ab}} - e^{-\mu S_{ac}} \big )/(1+2\mu ), $$

*and the same formula applies if c and d are swapped.*

### Decomposition of covariance

We begin by defining the total covariance of METAL distance estimates as

8 $$\begin{aligned} \left( \Sigma _\textrm{total}\right) _{ab,cd} {:}{=}\textrm{Cov}\left( \hat{d}^\mathrm{(METAL)}(a,b), \hat{d}^\mathrm{(METAL)}(c,d) \right) = \frac{1}{m} \textrm{Cov}\left( \hat{p}_{ab}^{(1)}, \hat{p}_{cd}^{(1)} \right) , \end{aligned}$$

where $$\Sigma _\textrm{total} \in \mathbb {R}^{\left( {\begin{array}{c}n\\ 2\end{array}}\right) \times \left( {\begin{array}{c}n\\ 2\end{array}}\right) }$$. The following proposition shows how the total covariance can be decomposed as a coalescent and substitution covariance.

#### **Proposition 5**

*The total covariance can be expressed as*

9 $$\begin{aligned} \Sigma _\textrm{total} = \frac{1}{m} \Sigma _\textrm{coal} + \frac{1}{mK}\Sigma _\textrm{sub}, \end{aligned}$$

*where*

10 $$\begin{aligned} \left( \Sigma _\textrm{coal}\right) _{ab,cd}&= \frac{9}{16} \textrm{Cov}\left( e^{-\mu g_{ab}^{(1)}}, e^{-\mu g_{cd}^{(1)}}\right) , \end{aligned}$$

11 $$\begin{aligned} \left( \Sigma _\textrm{sub}\right) _{ab,cd}&= \frac{3}{16} \mathbb {E}_{G^{(1)}| \mathcal {S}} \left( e^{-\mu \left( g_{ab}^{(i)} + g_{cd}^{(i)} \right) } \left( e^{2\mu \delta _{ab,cd}^{(i)}} + 2 e^{\mu \delta _{ab,cd}^{(i)}} - 3 \right) \right) , \end{aligned}$$

*and* $$\Sigma _\textrm{coal}, \Sigma _\textrm{sub}, \Sigma _\textrm{total} \in \mathbb {R}^{\left( {\begin{array}{c}n\\ 2\end{array}}\right) \times \left( {\begin{array}{c}n\\ 2\end{array}}\right) }$$ *are positive semi-definite matrices.*

The decomposition of Proposition 5 arises from conditioning on the latent gene trees and utilizing the law of total covariance: variation across loci due to random coalescent histories contributes the $\Sigma _\textrm{coal}$ term (scaled by 1/*m*), while conditional variation in the site patterns within each gene tree contributes the $\Sigma _\textrm{sub}$ term (scaled by 1/(*mK*)). Although both matrices are influenced by the coalescent and substitution processes, we use the terminology “coalescent covariance” and “substitutional covariance” to highlight which source of randomness dominates each component: $\Sigma _\textrm{coal}$ reflects the variability across gene trees even with infinitely long sequences, while $\Sigma _\textrm{sub}$ reflects the additional noise from having only finitely many sites per gene.

Expressions for $\Sigma _\textrm{coal}$ defined in Eq. (10) have been derived in the proof of Proposition 2. Specific expressions for $\Sigma _\textrm{sub}$ defined in Eq. (11), for all tree topology cases can be found in Appendix B.

The decomposition in Proposition 5 sets the stage for Proposition 6, in which we specialize to the case of a star-shaped species tree and derive explicit formulae and asymptotics for each component of covariance. These results allow use both to compare the magnitude of coalescent versus substitution uncertainty and to study how their ratio varies with the mutation rate *μ * and tree diameter *Δ *. While the star-tree setting of Proposition 6 does not capture the full complexity of species-tree inference, it provides a tractable case that highlights the relative contributions of coalescent and substitutional variance.

#### **Proposition 6**

*Suppose that the species tree is a star tree with diameter* *Δ* *i.e.* $\tau _{ab} = \Delta , \forall a,b \in L= [n], a\ne b$*. Then*

$$\begin{aligned} (\Sigma _\textrm{mode})_{ab,cd} = {\left\{ \begin{array}{ll} \sigma ^{(2)}_\textrm{mode}(\mu ,\Delta ), & \text{ if } |\{a,b\} \cap \{c,d\}| = 2 \\ \sigma ^{(1)}_\textrm{mode}(\mu ,\Delta ), & \text{ if } |\{a,b\} \cap \{c,d\}| = 1 \\ \sigma ^{(0)}_\textrm{mode}(\mu ,\Delta ), & \text{ if } |\{a,b\} \cap \{c,d\}| = 0, \end{array}\right. } \end{aligned}$$

*for* $$\textrm{mode} \in \{ \textrm{coal, sub, total} \}$$ *with*

$$\begin{aligned} \sigma _\textrm{total}^{(i)}(\mu ,\Delta ) = \frac{1}{m} \left( \sigma _\textrm{coal}^{(i)}(\mu ,\Delta ) + \frac{1}{K} \sigma _\textrm{sub}^{(i)}(\mu ,\Delta ) \right) , \, \forall i \in \{0,1,2\}, \end{aligned}$$

*where m is the number of loci and K is the number of sites per loci.*

*Moreover, *$$\sigma _\textrm{mode}^{(2)} \ge \sigma _\textrm{mode}^{(1)} \ge \sigma _\textrm{mode}^{(0)} > 0$$*, and*

$$\begin{aligned} \sigma _\textrm{coal}^{(i)}&= {\left\{ \begin{array}{ll} \mathcal {O}(\mu ^2), & \text { as } \mu \rightarrow 0, \\ \mathcal {O}(\mu ^{-3+i} e^{-2\mu \Delta }), & \text { as } \mu \rightarrow \infty \end{array}\right. } \\ \sigma _\textrm{sub}^{(i)}&= {\left\{ \begin{array}{ll} \mathcal {O}(\mu ), & \text { as } \mu \rightarrow 0, \\ \mathcal {O}(\mu ^{-2+i} e^{(-2+i)\mu \Delta }), & \text { as } \mu \rightarrow \infty \end{array}\right. } \\ \end{aligned}$$

*for all* $i \in \{0,1,2\}$ *and consequently*

$$\begin{aligned} \frac{\sigma _\textrm{sub}^{(i)} }{\sigma _\textrm{coal}^{(i)}}&= \mathcal {O}(\mu ^{-1}) \,\,\, \text { as } \mu \rightarrow 0, \forall i \in \{0,1,2\}, \textrm{and }\\ \frac{\sigma _\textrm{sub}^{(i)} }{\sigma _\textrm{coal}^{(i)}}&= \mathcal {O}(\mu e^{i \mu \Delta } ) \,\,\, \text { as } \mu \rightarrow \infty , \forall i \in \{0,1,2\}. \\ \end{aligned}$$

Proposition 1 shows that in the low-mutation regime ($\mu \ll 1$), STEAC and METAL agree to first order, since $p_{ab} \;=\;\tfrac{3}{4}\bigl (1-e^{-\mu g_{ab}}\bigr )\approx \tfrac{3}{4}\,\mu \,g_{ab}.$ However, a fundamental drawback of STEAC is that, for any positive value of *μ *, the distribution of the estimate $\hat{g}^{(i)}_{ab}$ has strictly positive probability of being infinite, because $\hat{p}_{ab}^{(i)}$ can exceed *3/4* with nonzero probability. Consequently, the variance of the STEAC estimator $\hat{g}_{ab}$ is infinite for all *μ *. This implies that with many loci the STEAC average is highly unstable: a single locus producing an infinite estimate suffices to drive the average to infinity. Although one can truncate or discard these infinite estimates, doing so introduces downward bias by systematically removing the highest inferred branch lengths. We conjecture that, in the presence of substitution uncertainty–i.e. when gene trees must be estimated rather than known a priori–STEAC is uniformly inferior to METAL for all *μ *; indeed, STEAC was originally developed under the assumption of almost perfect gene-tree knowledge. We assess this conjecture empirically in Sect. “Species-tree estimation”.

### Principal components of variance

As we have seen in Proposition 6, the coalescent, substitution and total covariance matrices of the METAL estimator for the star species tree only depend on $|\{a,b\} \cap \{c,d\}|$. In this subsection we study the spectrum of these matrices. Specifically, for a covariance matrix $$C \in \mathbb {R}^{\left( {\begin{array}{c}n\\ 2\end{array}}\right) \times \left( {\begin{array}{c}n\\ 2\end{array}}\right) }$$, with entries

12 $$\begin{aligned} C_{ab,cd} = \textrm{Cov}\left( \hat{d}_{ab}^{\mathrm{(METAL)}}, \hat{d}_{cd}^{\mathrm{(METAL)}} \right) = {\left\{ \begin{array}{ll} \alpha , & \text{ if } |\{a,b\} \cap \{c,d\}| = 2 \\ \beta , & \text{ if } |\{a,b\} \cap \{c,d\}| = 1 \\ \gamma , & \text{ if } |\{a,b\} \cap \{c,d\}| = 0 , \end{array}\right. } \end{aligned}$$

for pairs $$(a,b), (c,d) \in \left( {\begin{array}{c}L\\ 2\end{array}}\right) $$ where $\alpha> \beta> \gamma > 0$, Proposition 7 derives its spectrum.

#### **Proposition 7**

*The matrix *$C \in \mathbb {R}^{ {n \atopwithdelims ()2} \times {n \atopwithdelims ()2} }, n\ge 3$*, defined in Eq. (**12**), has eigenvalues*

- *the dominant eigenvalue *$\gamma {n \atopwithdelims ()2} + 2(\beta -\gamma )(n-1) + \gamma + \alpha - 2\beta$*with multiplicity 1 and corresponding first principal component *$\textrm{PC1} =\textbf{1},$
- $(\beta -\gamma )(n-2) +\gamma + \alpha - 2 \beta$ *with corresponding principal components *$\textrm{PC}2, \dots , \textrm{PC}{n}$ *dependent on n and independent of* $\alpha ,\beta ,\gamma$*, and*
- $$\gamma + \alpha - 2\beta $$ *with multiplicity* *n(n-3)/2.*

*Note that these eigenvalues are presented in decreasing magnitude and that C* *is positive definite if and only if* $\alpha + \gamma > 2\beta$*. It follows that the variance ratio of the principal component tends to *$\frac{\gamma }{\alpha }$ *as*$n \rightarrow \infty$*. The variance ratio of the first n* *most dominant eigenvectors tends to* $\frac{2\beta - \gamma }{\alpha }$ *as* $n \rightarrow \infty .$

Proposition 7 shows that each covariance matrix in our model has exactly three distinct eigenvalues. In particular, the all-ones vector $\textbf{1}$ is an eigenvector corresponding to the largest eigenvalue. From the perspective of tropical geometry, $\textbf{1}$ plays a canonical role: the tropical projective torus $$ \mathbb {R}^{\left( {\begin{array}{c}n\\ 2\end{array}}\right) } \big / \mathbb {R}\textbf{1} $$ is precisely the quotient that identifies any vector $\textbf{v}$ with its translate $\textbf{v} + c\,\textbf{1}$ for all real *c*. Equivalently, adding a constant to every coordinate does not change the point in the toric quotient. Statistical analysis on the tropical projective torus has been explored in various works, e.g. [18–20].

Corollary 1 (to Proposition 7) further quantifies that up to one-third of the total variance of our distance-vector estimates lies along the $\textbf{1}$-direction. Since the tropical projective torus “modes out” precisely the $\textbf{1}$-direction, restricting our inference to $$\mathbb {R}^{\left( {\begin{array}{c}n\\ 2\end{array}}\right) } / \mathbb {R}\textbf{1}$$ removes this single principal-component subspace. As a result, the remaining variance–now confined to the $$(\left( {\begin{array}{c}n\\ 2\end{array}}\right) -1)$$-dimensional tropical torus–drops by up to one-third. Working in this quotient removes a large portion of extraneous variation while preserving all information relevant to tree topology, since adding any constant multiple of $\textbf{1}$ to the distance vector does not alter the inferred tree topology.

**Fig. 3:**
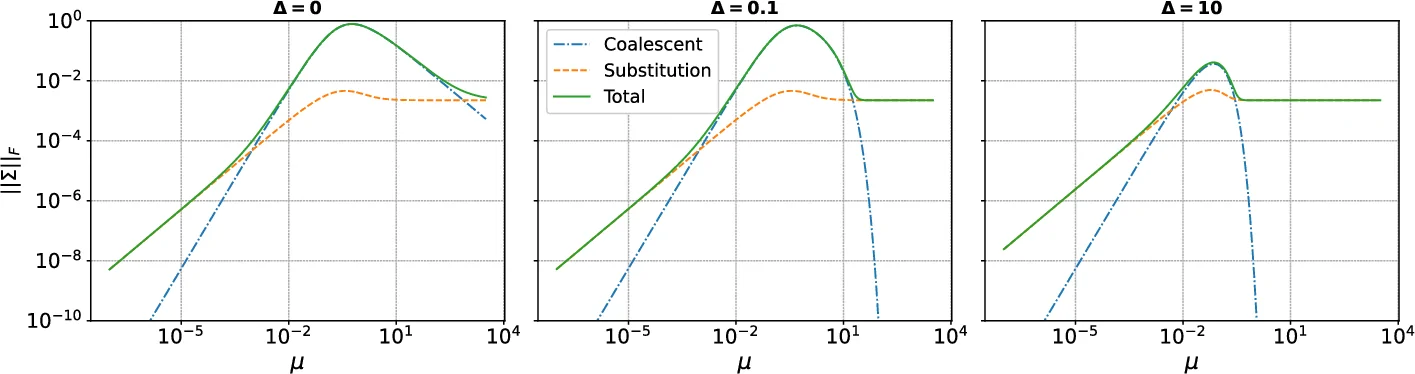
Frobenius norms of $\Sigma _\textrm{coal}(\mu )$, $\Sigma _\textrm{sub}(\mu )$, and $\Sigma _\textrm{total}(\mu )$ as functions of the mutation rate *μ *, shown for three values of the species-tree diameter *Δ * with *K = 1000*. For $\mu \ll 1$ and for $\mu \gg 1$, the substitution variance $\Sigma _\textrm{sub}$ dominates; in the intermediate *μ * range, the coalescent variance $\Sigma _\textrm{coal}$ prevails. As *Δ * increases, the interval in which $\Sigma _\textrm{coal}$ dominates becomes narrower

**Fig. 4:**
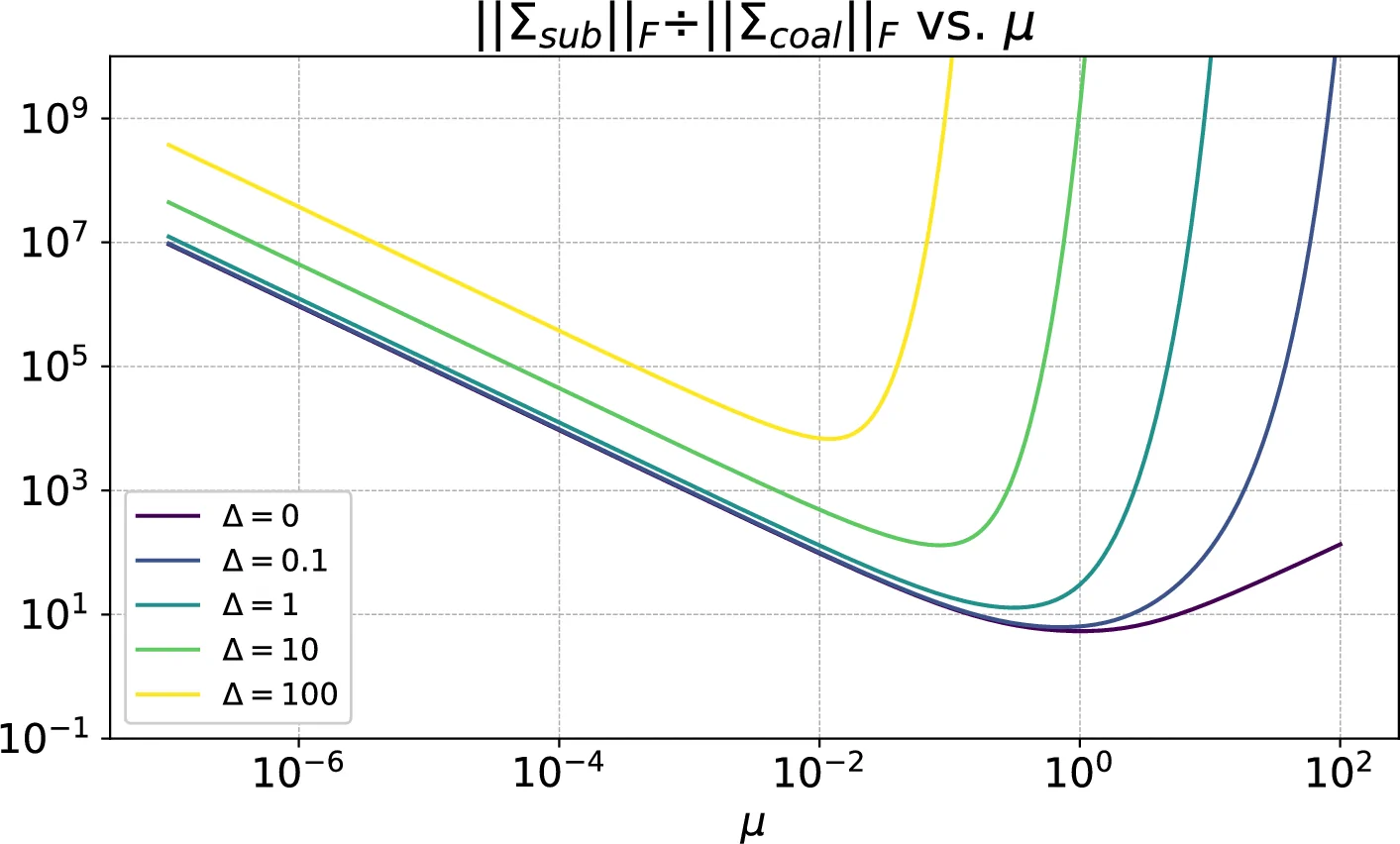
Ratio $\Vert \Sigma _\textrm{sub}(\mu )\Vert _F \big / \Vert \Sigma _\textrm{coal}(\mu )\Vert _F$ plotted against *μ * for several values of *Δ *. Substitution variance ($\Sigma _\textrm{sub}$) dominates when $\mu \ll 1$ or $\mu \,\Delta \gg 1$, whereas coalescent variance dominates in the intermediate *μ * regime

**Fig. 5:**
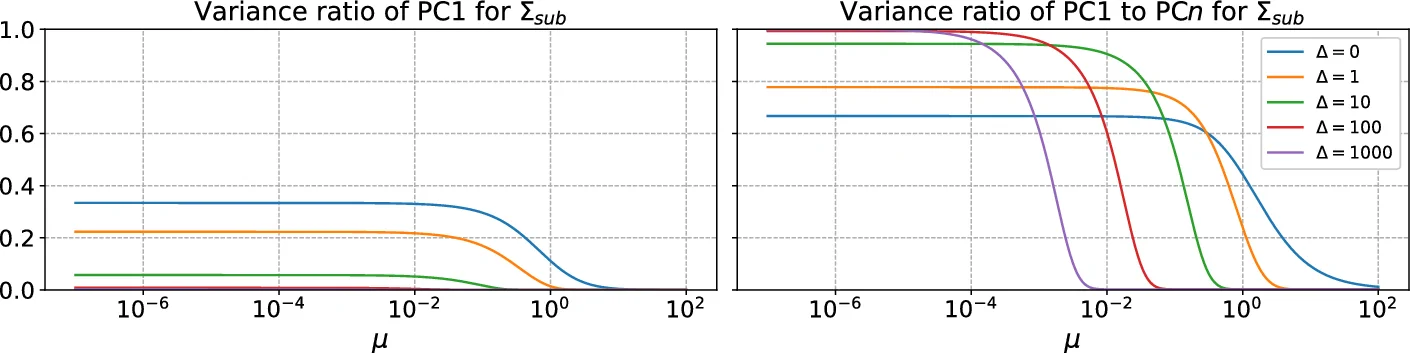
Variance ratios for $\Sigma _\textrm{sub}$ plotted against mutation rate *μ * and species-tree diameter *Δ *. (Left) Fraction of total variance explained by the first principal component. (Right) Fraction of total variance explained by the first *n* principal components. For corresponding plots of $\Sigma _\textrm{coal}$ and $\Sigma _\textrm{total}$, see Fig. 13 in the Appendix

Moreover, Corollary 1 shows that when $\mu \ll 1$, the relative magnitude of substitution versus coalescent uncertainty scales with *μ K*, the expected number of substitutions per locus: as *μ K* increases, substitution noise becomes negligible. In particular, if

13 $$\begin{aligned} K^{-1} \ll \mu \ll \frac{1}{2\Delta } \log \left( \frac{1+\sqrt{8K\Delta }}{2} \right) , \end{aligned}$$

then the coalescent covariance term dominates the total uncertainty. Note that as the number of bases per gene *K* increases, the interval widens on both ends, whereas increasing *Δ * narrows the interval from the upper bound. Figure 3 confirms this behavior: substitution-driven variance is largest for very small and very large *μ *, whereas for intermediate *μ * the coalescent variance is predominant. Figure 4 plots the ratio of Frobenius norms $\Vert \Sigma _\textrm{sub}\Vert _F/\Vert \Sigma _\textrm{coal}\Vert _F$, which attains its minimum at $\mu = \mathcal {O}(1/\Delta )$ when *Δ * is large. Finally, Fig. 5 displays the cumulative variance explained by the leading *n* principal components of $\Sigma _\textrm{sub}$. Remarkably, for $\mu \ll 1$ and large *Δ *, these *n* components capture nearly all of the METAL estimator’s variance, while for larger *μ * the explained fraction vanishes, in agreement with Proposition 8.

It is worth mentioning that Proposition 8 further establishes that as $\mu \rightarrow \infty$, the limiting coalescent correlation matrix, as defined in Eq. (14), has rank *n-1*, and its nonzero principal components are the split-indicator vectors which are exactly the generators of the unique maximal cone of the ultrametric fan that contains the species-tree ultrametric. In other words, all variation of the METAL distance vector lies within the cone corresponding to the true species-tree ultrametric.

#### **Corollary 1**

*Consider the matrices* $$\Sigma _\textrm{coal}(\mu ), \Sigma _\textrm{sub}(\mu ), \Sigma _\textrm{total}(\mu ) \in \mathbb {R}^{\left( {\begin{array}{c}n\\ 2\end{array}}\right) \times \left( {\begin{array}{c}n\\ 2\end{array}}\right) }$$* defined in Eqs. (*8*), (*10*), (*11*) for a star species tree with diameter* *Δ **. They have the following asympototic properties*

$$\begin{aligned} \frac{ \textrm{Tr}(\Sigma _\textrm{sub} )}{ \textrm{Tr}(\Sigma _\textrm{coal} )}, \root \left( {\begin{array}{c}n\\ 2\end{array}}\right) \of { \frac{\det (\Sigma _\textrm{sub} )}{ \det (\Sigma _\textrm{coal} )}}, \frac{\left\Vert \Sigma _\textrm{sub} \right\Vert _2 }{\left\Vert \Sigma _\textrm{coal} \right\Vert _2 }, \frac{\left\Vert \Sigma _\textrm{sub} \right\Vert _\textrm{F} }{\left\Vert \Sigma _\textrm{coal} \right\Vert _\textrm{F} }= {\left\{ \begin{array}{ll} \mathcal {O}(\mu ^{-1}K^{-1}), & \text { as } \mu \rightarrow 0, \\ \mathcal {O}(\mu e^{2\mu \Delta } K^{-1} ), & \text { as } \mu \rightarrow \infty \end{array}\right. } \\ \end{aligned}$$

*As* $\mu \rightarrow 0$ *and* $n \rightarrow \infty$*, the first principal component PC1=****1*** *explains* *2/(6+3Δ )* *of the variance of *$\Sigma _\textrm{evol}$ *and* $\Sigma _\textrm{total}$*, and 2/9 of the variance of* $\Sigma _\textrm{coal}$*, while the first n* *principal components explain* $(4+3\Delta )/(6+3\Delta )$ *and 4/9 of the variance respectively.*

#### *Proposition 8*

*Consider a species tree S with positive pair distances* $\tau _{i,j} = \rho _{i,j} \Delta$*, where **Δ* *is the diameter of the tree and* $\rho _{ij} \in (0,1]$*. From the covariance matrix defined in Eq. (*10*), if we construct the correlation matrix* $C_\textrm{coal}(\Delta ) = D_\textrm{coal}^{-1} \Sigma _\textrm{coal}(\Delta ) D_\textrm{coal}^{-1}$*, where *$D_\textrm{coal} = \sqrt{\textrm{diag}(\Sigma _\textrm{coal}(\Delta ))}$ *and consider the limiting case for large trees, then we let*

14 $$\begin{aligned} C_\textrm{coal}^{\infty } {:}{=}\lim _{\Delta \rightarrow \infty } C_\textrm{coal}(\Delta ). \end{aligned}$$

*Let* $i \in V\backslash L$ *be an internal node of the species tree* *S, and the sets* $L_i,R_i \subset L = [n]$ *are the sets of leaf nodes left and right of i respectively. Define the vector,*

$$\begin{aligned} v^{(i)}_{kl}&{:}{=}\mathbb {I} \left( i \text { is the most recent common ancestor of } k \text { and } l \right) \\ &= \mathbb {I} \left( (k,l) \in (L_i \times R_i) \cup (R_i \times L_i) \right) , \end{aligned}$$

*which are also known as split-indicator vectors. Then,* $C_\textrm{coal}^{\infty } v^{(i)} = |L_i| |R_i| v^{(i)}$ *i.e.* $v^{(i)}$ *is an eigenvector with corresponding eigenvalues* $|L_i| |R_i|$ *. These are exactly* *n-1* *principal components corresponding to distinct internal nodes in **V\backslash L* *. The remaining eigenvalues are zero, and so *$\textrm{rank}(C_\textrm{coal}^{\infty }) = n-1$ *. Finally, let the species tree pairwise distance vector *$$d^\textrm{S} \in \mathbb {R}^{\left( {\begin{array}{c}n\\ 2\end{array}}\right) \times \left( {\begin{array}{c}n\\ 2\end{array}}\right) },$$ $d^\textrm{S}_{ab} = \tau _{ab},\, \forall a,b \in L$ *be an ultrametric i.e.* $d \in \mathcal {U}_n$ *and suppose it does not lie on topological boundaries. Then, there exists* $\delta _i > 0$ *such that for all* $\epsilon _i \in (0,\delta _i),$

$$\begin{aligned} d^\textrm{S} + \sum _{i = 1}^{n-1} \epsilon _i v^{(i)} \in \mathcal {U}_n \end{aligned}$$

*Finally,* $$C_\textrm{sub}^{\infty } = C_\textrm{total}^{\infty } = I_{\left( {\begin{array}{c}n\\ 2\end{array}}\right) }$$ *, where the correlation matrices are defined as above, and* $I_N \in \mathbb {R}^{N\times N}$ *is the identity matrix.*

## Simulation results

Two applications of the derived covariance matrix in phylogenetic analysis are considered in this section. In the first application (Sect. “Species-tree estimation”), our derivations of the covariance matrix enable us to quantify uncertainty from the substitution model, which predicts the relative performance of GLASS against METAL. In the second application (Sect. “Bipartition confidence”), the covariance matrix is used to compute node-support values as confidence for each bipartition of the estimated METAL tree. We compare our method to the standard bootstrap approach. Our repository can be found in [21].

### Species-tree estimation

We begin by simulating data under the multi-species coalescent (MSC) combined with a site-substitution model. Using default parameters

$$ \mu = 1,\quad \Delta = 1,\quad K = 100,\quad n = 10,\quad m = 100, $$

we first generate a species tree of diameter *Δ * (in coalescent units) via the Yule model. Then *m* gene trees are sampled under MSC on that species tree. Finally, sequences of length *K* are evolved along each gene tree under a substitution model with rate *μ *, producing *m* alignments.

Under these default conditions, the simulated alignments contain approximately *0.3%* constant sites, corresponding to a relatively high mutation rate (*μ = 1*). Although this rate is higher than what is typically observed in empirical datasets, we use it deliberately to keep the simulations tractable. Using a more realistic mutation rate (e.g., *μ = 0.01*) would result in over *99%* constant sites. In simulated data, compensating for this would require increasing the number of sites per genes *K*by two orders of magnitude, making simulations computationally impractical. Moreover, the additional constant sites would contribute no information to any inference method. Hence, we adopt the higher mutation rate solely to ensure that the simulated datasets contain a reasonable proportion of informative sites, and not because the method requires unrealistically high mutation rates in practice.

From these *m* alignments, we infer the species tree using three approaches (see Algorithm 2): STEAC, METAL, and GLASS. In STEAC and GLASS, each gene’s pairwise Hamming distances $\hat{p}^{(i)}_{ab}$ are transformed via the inverse Jukes–Cantor formula to branch-length estimates $\hat{g}^{(i)}_{ab}$. STEAC then averages across all *m* genes before applying UPGMA. GLASS takes the minimum branch gene tree branch lengths before applying UPGMA. METAL concatenates all *m* alignments, computes Hamming distances on the full superalignment, and reconstructs a UPGMA tree from that distance matrix. Here, UPGMA is an appropriate choice for phylogenetic inference since it will return ultrametric trees, compatible with our covariance decomposition.

**Fig. 6:**
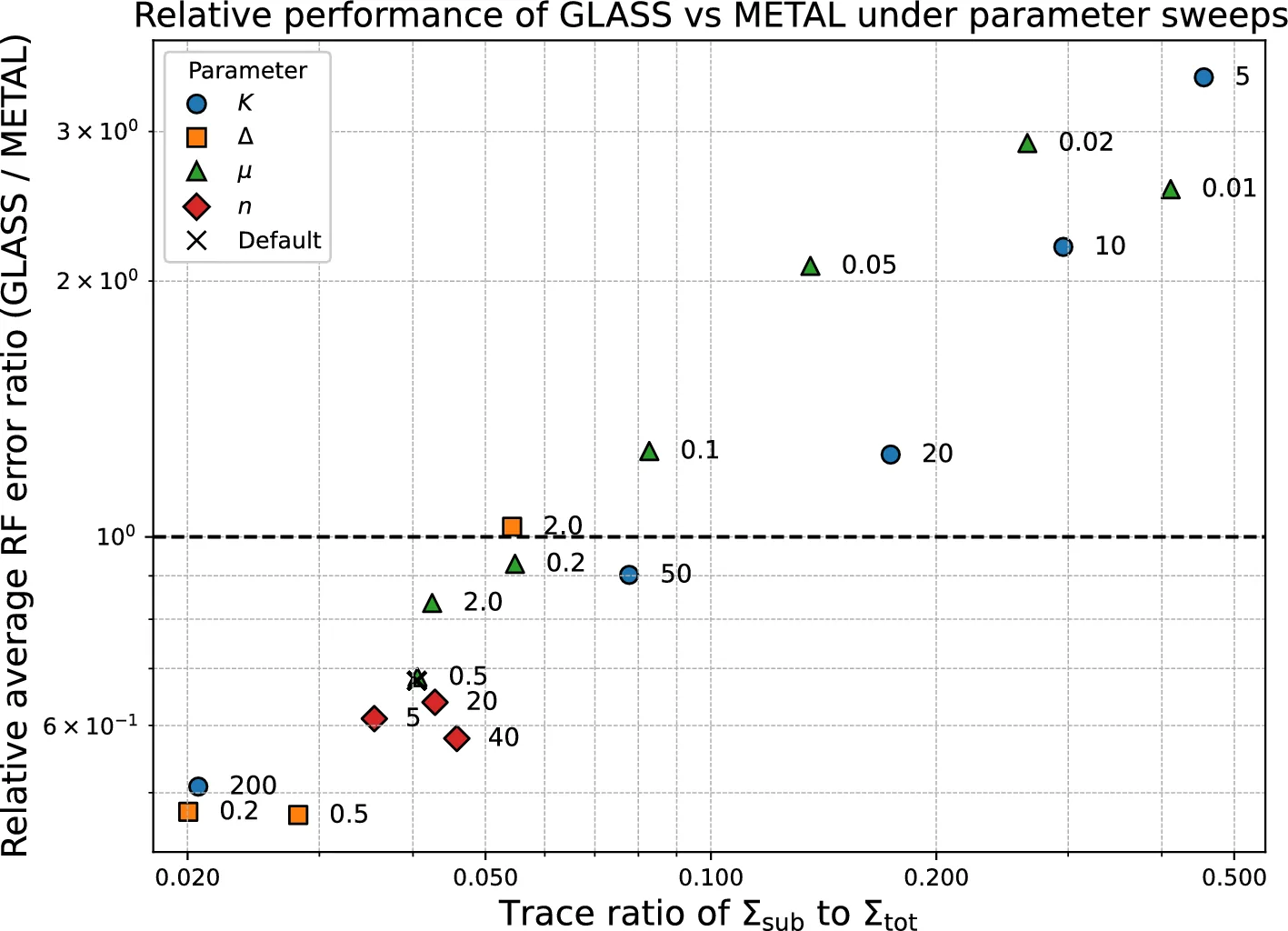
Comparison of GLASS and METAL performance as a function of the ratio $\textrm{tr}(\Sigma _{\textrm{sub}})/\textrm{tr}(\Sigma _{\textrm{total}})$. When substitution-model variance constitutes a large fraction of total variance, METAL outperforms GLASS

Figure 6 illustrates how the relative accuracy of GLASS versus METAL depends on the fraction of variance contributed by the substitution model. As this ratio increases, METAL (which explicitly pools substitution noise across genes) consistently outperforms GLASS. To quantify reconstruction accuracy, each inferred tree $\widehat{T}_{\textrm{method}}$ is compared against the true species tree $T_{\textrm{true}}$ via the Robinson–Foulds (RF) distance. Over *R* replicates (here *R=100*), we obtain RF distances $\textrm{RF}_{\textrm{STEAC}}^{(r)},\,\textrm{RF}_{\textrm{METAL}}^{(r)},\,\textrm{RF}_{\textrm{GLASS}}^{(r)}$. The mean RF error for each method is

$$ \overline{\textrm{RF}}_{\textrm{method}} \;=\; \frac{1}{R}\sum _{r=1}^{R} \textrm{RF}_{\textrm{method}}^{(r)}. $$

We consistently observe

$$ \overline{\textrm{RF}}_{\textrm{STEAC}} \;>\; \overline{\textrm{RF}}_{\textrm{METAL}}, $$

so STEAC underperforms METAL in all tested regimes (see Fig. 14 in the Appendix). In contrast,

$$ \frac{\overline{\textrm{RF}}_{\textrm{GLASS}}}{\overline{\textrm{RF}}_{\textrm{METAL}}} $$

increases with $\textrm{tr}(\Sigma _{\textrm{sub}})/\textrm{tr}(\Sigma _{\textrm{total}})$. When substitution variance dominates, GLASS yields higher RF error than METAL. However, if coalescent variance from MSC is the primary source (for instance, when *K* is large or *μ * lies in an intermediate regime), GLASS approaches the Kullback–Leibler-optimal species-tree estimator and outperforms METAL, i.e.

$$ \overline{\textrm{RF}}_{\textrm{GLASS}} \;<\; \overline{\textrm{RF}}_{\textrm{METAL}}. $$

In summary, the ratio

$$ \frac{\textrm{tr}(\Sigma _{\textrm{sub}})}{\textrm{tr}(\Sigma _{\textrm{total}})} $$

determines the preferable method: if this ratio exceeds a threshold (when *μ * is very low or very high, *Δ * is large, or *K* is small), METAL–by aggregating substitution noise–yields lower RF error. Otherwise, for moderate *μ * and large *K*, the coalescent term dominates and GLASS (leveraging accurate gene-tree estimates) gives superior accuracy.

### Bipartition confidence

The procedure begins with data generation: we first simulate a species tree, then simulate gene trees under the multi-species coalescent (MSC) model using DendroPy [22], and finally perform sequence evolution along those gene trees using Pyvolve [23]. This pipeline is illustrated in Fig. 1.

Once we have simulated data, we obtain the METAL estimate by computing Hamming distances between the concatenated sequences. These distances serve as pairwise dissimilarities, and we reconstruct a tree via the UPGMA algorithm, following the METAL methodology.

Our main objective is to assign confidence scores to tree splits (bipartitions). The standard approach uses bootstrapping: sites from the concatenated alignment are resampled (with replacement) to generate pseudo-replicate alignments. For each replicate, we compute a new METAL tree, yielding a collection of bootstrap trees. The proportion of replicates in which a given split appears provides its bootstrap support.

As an alternative, we propose a Gaussian-sampling approach. The METAL estimator of the average pairwise distances can, for a sufficiently large number of genes, be approximated by a multivariate normal distribution. The mean of this distribution is the METAL estimate itself, and the covariance is derived from the METAL tree (in coalescent units to scale Hamming distances appropriately). The quality of this normal approximation improves as the number of genes increases.

We draw samples from this multivariate distribution to obtain synthetic vectors of pairwise distances. Each sampled distance matrix is then converted into a tree via UPGMA, exactly as in the bootstrap procedure. We estimate split support by computing the frequency of each bipartition across these “Gaussian-sampled” trees.

**Fig. 7:**
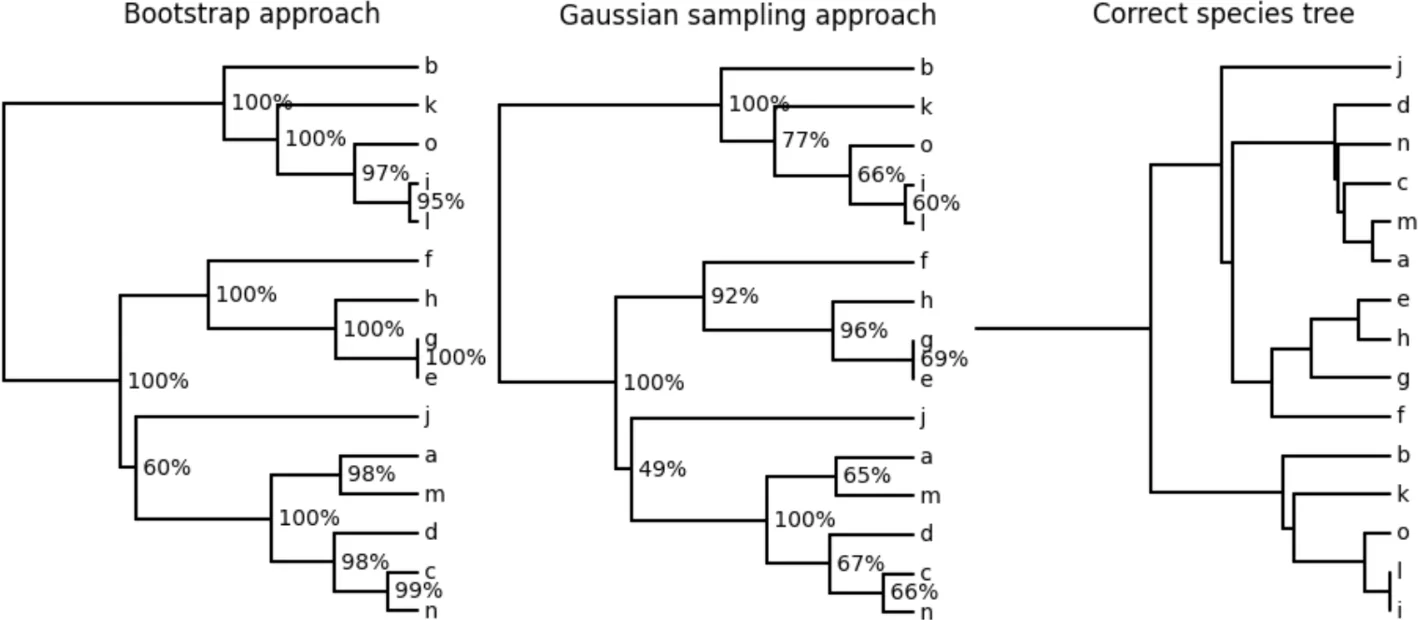
Example of METAL estimate (left) with bootstrap support (percentages at each node), METAL estimate (center) with Gaussian-sampling support, and the true species tree (right). Bootstrap supports tend to be higher than Gaussian supports, but may also overestimate confidence for incorrect splits

Figure 7 provides an illustrative comparison. The leftmost tree is the METAL estimate with bootstrap-derived support values, the middle tree shows the same METAL topology but with support values from Gaussian sampling, and the rightmost tree is the true species topology. Note that Gaussian sampling typically yields more conservative (lower) support values than bootstrapping.

To compare the two confidence-estimation methods quantitatively, we exploit the fact that the true species tree is known in simulation. With *n = 40* taxa, there are *n - 2 = 38* non-trivial splits. We label each split in the METAL estimate as correct (1) if it appears in the true tree, or incorrect (0) otherwise. Using the support scores assigned by each method, we compute the area under the ROC curve (AUC) to measure how well each set of support values discriminates correct splits from incorrect ones.

This experiment is repeated 100 times. In each series of 100 replicates, we vary exactly one simulation parameter (e.g., mutation rate *μ *) while holding all other parameters (species-tree diameter *Δ *, number of sites per gene *K*, and number of genes *m*, number of taxa *n*) fixed. We then repeat separately for each of the other parameters (*Δ *, *K*, *m*, and *n*), always keeping the remaining factors constant.

**Fig. 8:**
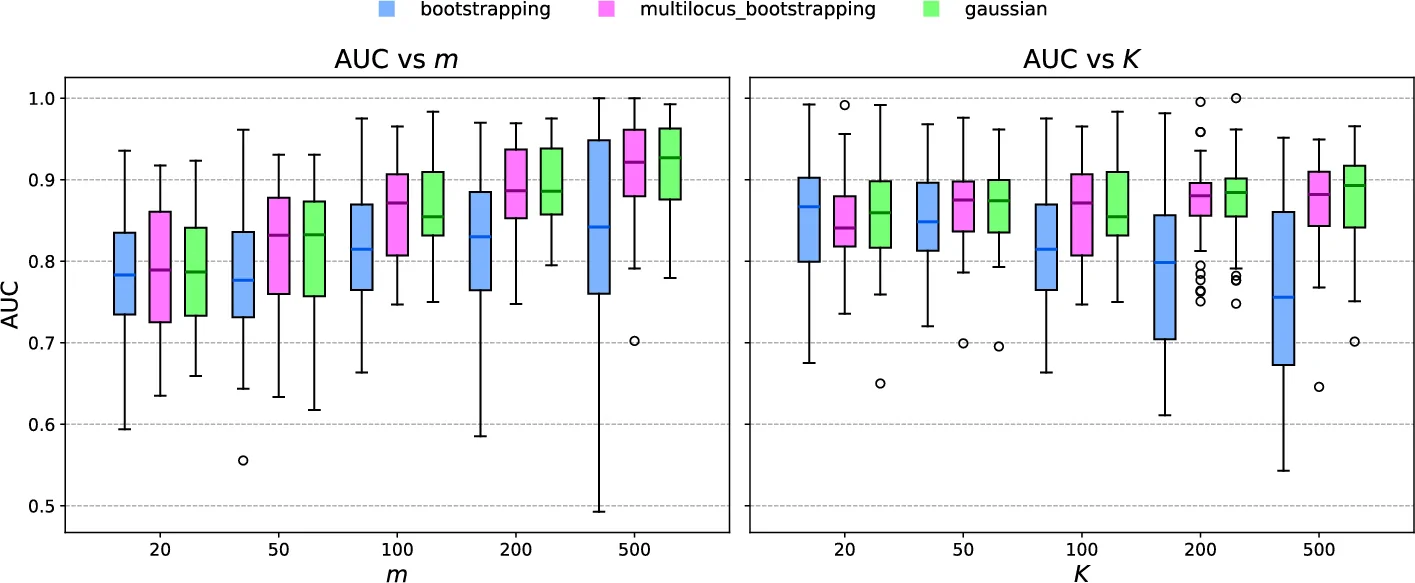
Boxplots of AUC values comparing standard bootstrap (blue), Gaussian sampling (green), and multilocus bootstrap (magenta) support scores. (Left:) AUC distributions as the number of genes *m* varies (with *μ *, *Δ *, *n*, and *K* held constant). (Right:) AUC distributions as the number of sites per gene *K* varies (with *μ *, *Δ *, *n*, and *m* held constant). Gaussian sampling and multilocus bootstrapping yield similar median AUC and lower variability than standard bootstrapping, while Gaussian sampling retains a clear computational advantage

Figure 8 shows boxplots of AUC scores for three methods: standard bootstrapping (blue), Gaussian sampling (green), and multilocus bootstrapping (magenta). In the left panel, we vary the number of genes *m*; in the right panel, we vary the number of sites per gene *K*. In both cases, Gaussian-sampling and multilocus bootstrapping yield higher median AUC and lower variance than standard bootstrapping. Similar figures for the other parameters can be found in Appendix D. What is interesting about parameters *m* and *K* is that the concatenated sequence length has *mK* bases. As the number of genes *m* or the number of sites per gene *K* increases, the runtime of standard and multilocus bootstrap procedures grows roughly in proportion to *m · K*, the size of the concatenated sequence. In contrast, the computational cost of the Gaussian-sampling approach is essentially unaffected by changes in *m* or *K*, since it depends only on the number of taxa *n*. Moreover, although standard bootstrapping requires more time as *m* and *K* increase, its ability to correctly classify true splits does not improve–in fact, its classification accuracy becomes more erratic. Nonetheless, for low values of both *m* and *K*, the results of gaussian sampling can be slightly worse than those of multilocus bootstrapping as can be seen in Fig. 17 of Appendix D.

#### Computational complexity comparison

For bootstrapping, each replicate requires recomputing the METAL tree from a resampled alignment of total length *m · K*. Computing all pairwise Hamming distances is $\mathcal {O}(n^2 \cdot m \cdot K)$, since there are $$\left( {\begin{array}{c}n\\ 2\end{array}}\right) = \mathcal {O}(n^2)$$ taxon pairs. To ensure METAL is accurate with probability *1 - ɛ *, one needs

$$ m \;=\; O\!\bigl (\tfrac{\log (n/\varepsilon )}{\tau ^2}\bigr ), $$

where *τ * is the shortest internal branch length. Since $\tau \le \Delta / (n - 1)$, one obtains $m = \Omega (n^2 \log n)$. Hence each bootstrap replicate costs $\mathcal {O}(n^4 \log n \cdot K)$, and obtaining *B* replicates is *B* times that.

In contrast, the Gaussian sampling approach requires computing a covariance matrix over

$$ N = \left( {\begin{array}{c}n\\ 2\end{array}}\right) \;=\; \mathcal {O}(n^2) $$

entries, resulting in a full covariance matrix of size *N × N*, containing $\mathcal {O}(n^4)$ elements. Computing the Cholesky decomposition of this matrix takes $\mathcal {O}(N^3) = \mathcal {O}(n^6)$ time, but only once. Each subsequent multivariate-normal sample (to produce one synthetic distance vector) costs $\mathcal {O}(N^2) = \mathcal {O}(n^4)$. Crucially, this Gaussian method is independent of the sequence length *K*, making it far more efficient when *K* is large.

**Fig. 9:**
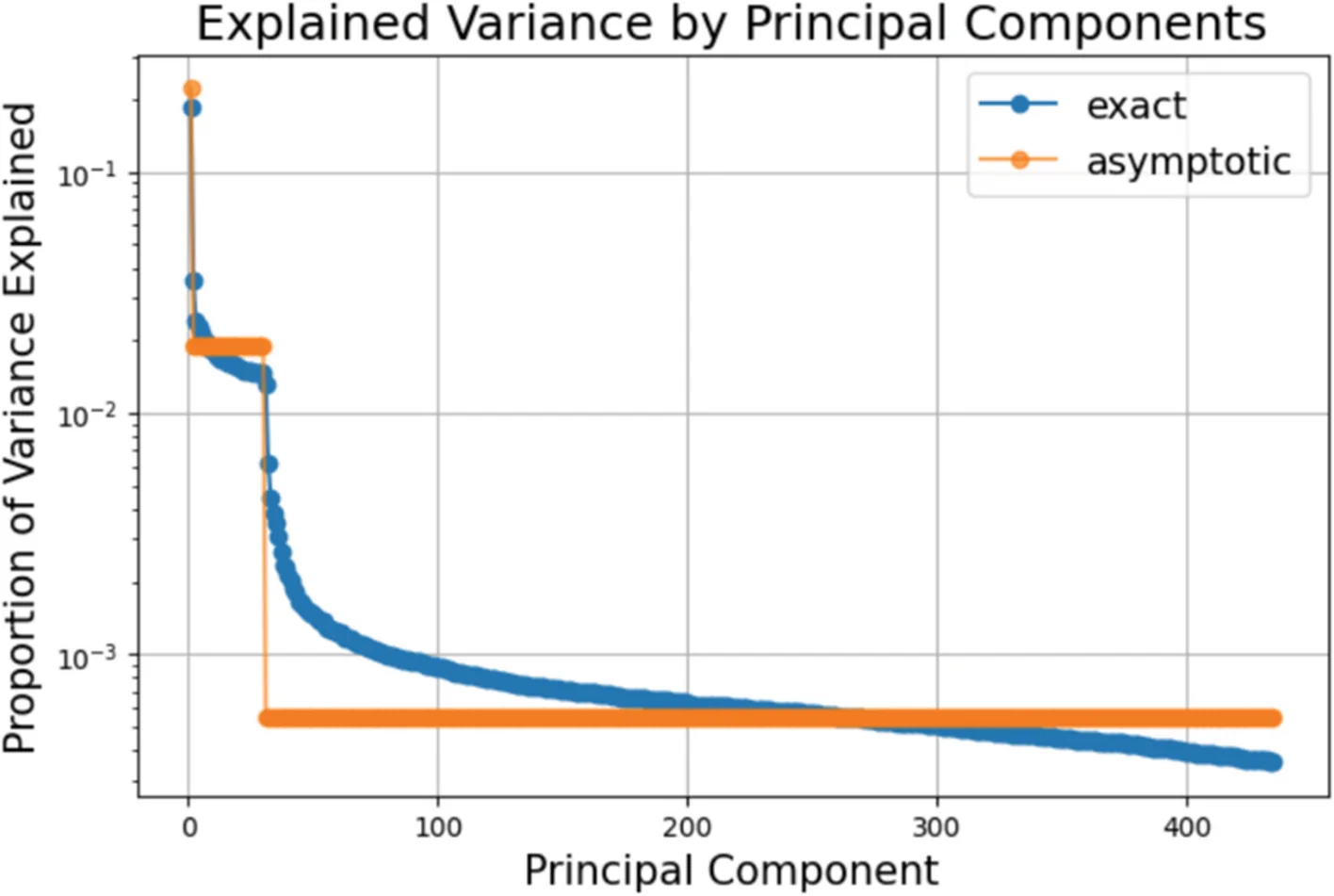
Explained variance by principal components of the total covariance matrix for a species tree with $\Delta =1, n =30, \mu =0.2, K= 100$. The exact curve (blue) shows the empirical proportion of variance explained by each principal component, while the "asymptotic" curve (orange) represents theoretical predictions from Corollary 1. The close agreement validates the asymptotic spectrum in the low mutation rate regime ( $\mu \ll 1$ )

**Fig. 14:**
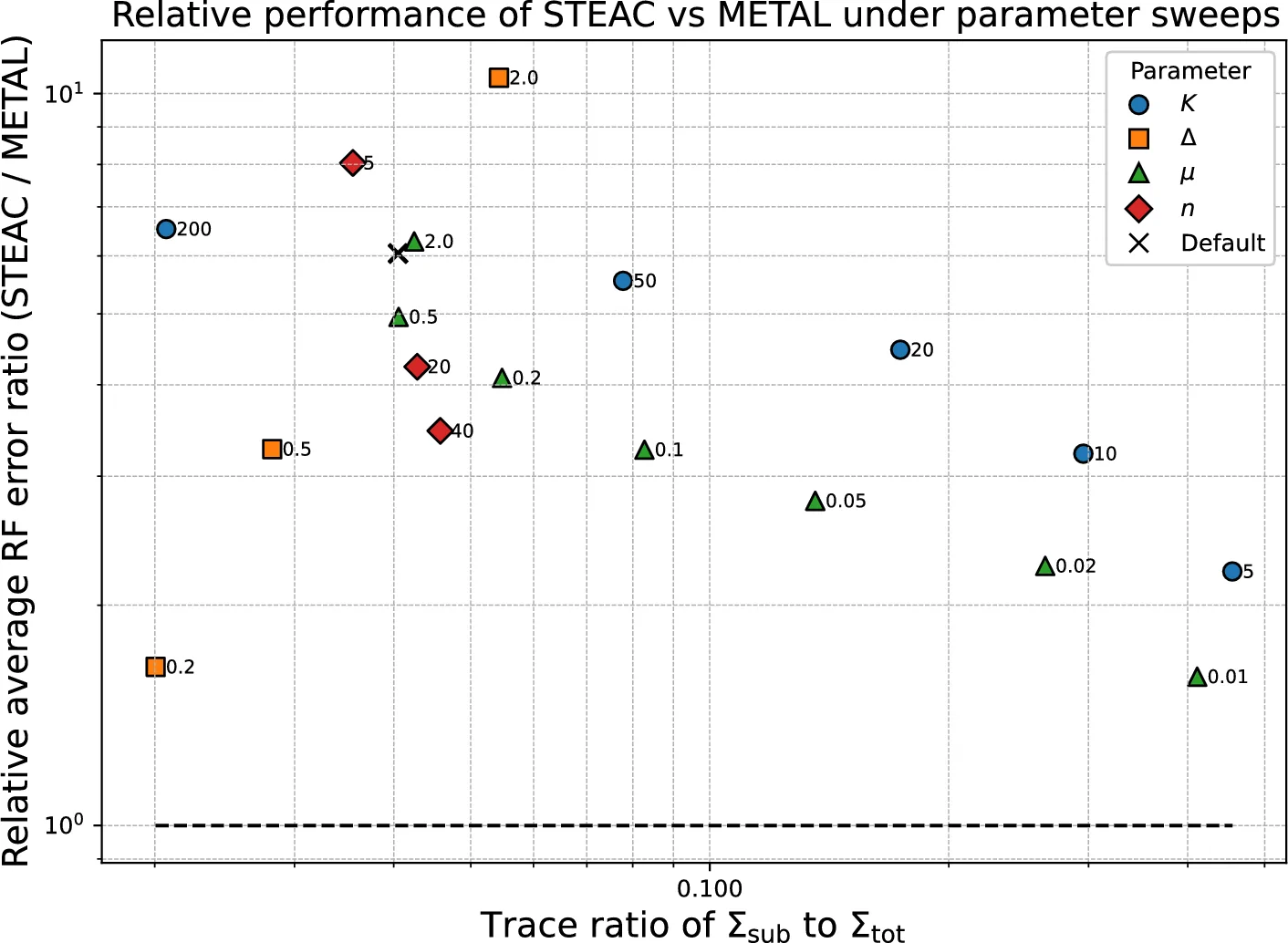
This Figures compares STEAC to METAL, similarly to Fig. 6. In all cases METAL outperforms STEAC

**Fig. 17:**
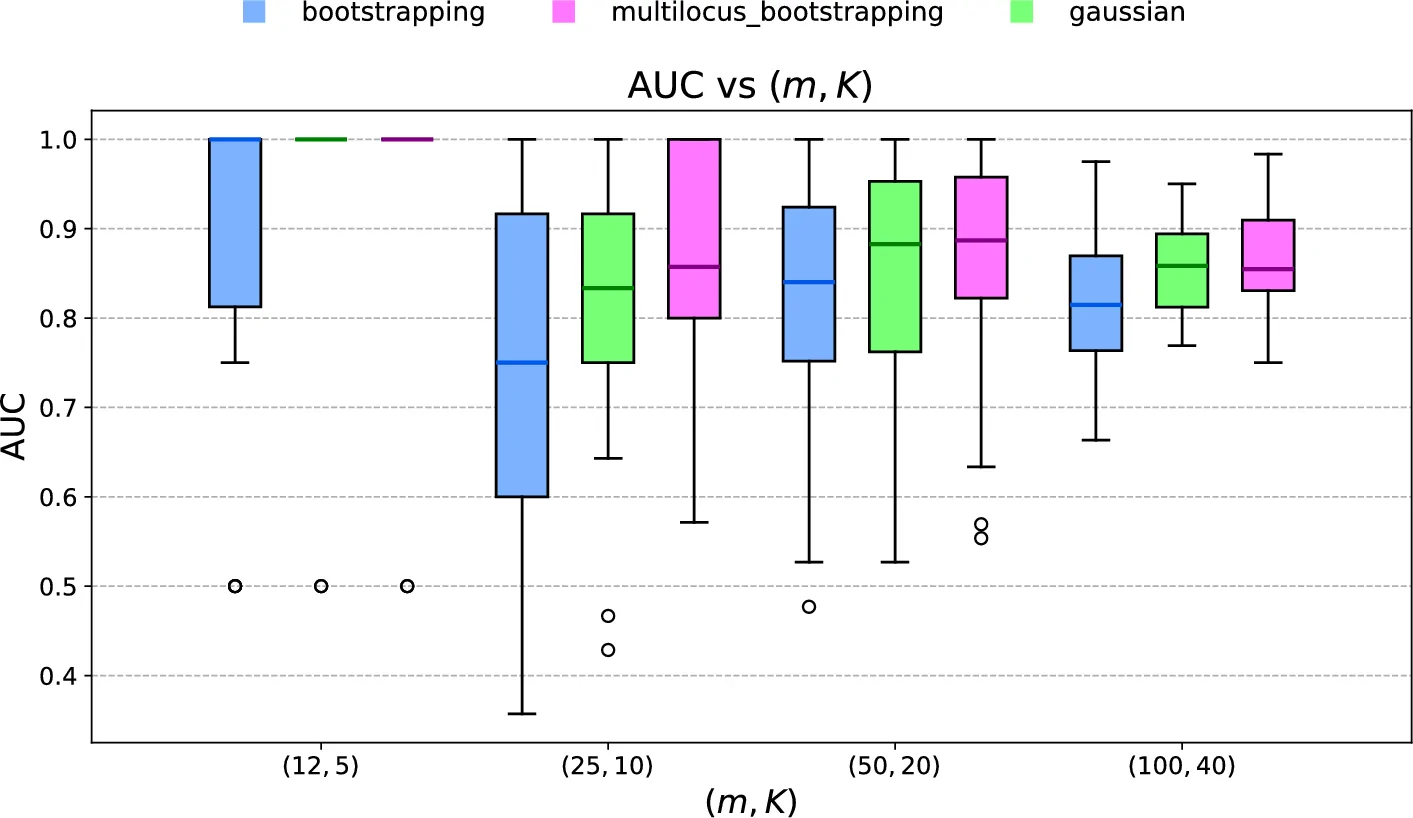
Boxplots of AUC values for different pairs (*m*, *K*) of number of genes and number of sites per gene, increasing together. For low values of both *m* and *K*, the results of gaussian sampling can be slightly worse than those of multilocus bootstrapping

Nonetheless, when the number of taxa is very large *(n ≫ 1)*, the one-time cost of the Cholesky decomposition becomes prohibitive, and bootstrapping may be more efficient. However, if the mutation rate is low $\mu \ll 1$ or if $\mu \Delta \gg 1$, we can instead leverage the theoretical results of Sect. “Theoretical results”, specifically Corollary 1 and Proposition 8. These results describe the asymptotic spectrum of the total covariance matrix of the METAL estimator. In both of these limiting cases, we have shown in Proposition 6 that $\Sigma _\textrm{coal} \ll \Sigma _\textrm{sub}$, and so that $\Sigma _\textrm{total} \approx \Sigma _\textrm{sub}$. For $\mu \ll 1$, there are three distinct eigenvalues with known variance ratios. As illustrated in Fig. 9, the asymptotic predictions of the eigenvalues of Corollary 1 closely match the empirical ones of a randomly generated species tree with $\Delta =1, n =30, \mu =0.2$. Knowing the principal components in advance allows us to bypass the Cholesky decomposition entirely, eliminating the most computationally expensive step of the method.

## Discussion

In this work, we explicitly formulate the covariant matrix of all pairwise distances under the MSC and substitution models for the species tree reconstruction problem. METAL constructs a distance matrix from concatenated multi-locus data [11], and then applies a clustering algorithm to infer the species tree. Our first main result is the derivation of the exact covariance matrix of METAL’s pairwise distance estimates. This covariance naturally decomposes into a term arising from coalescent genealogy variance and a term from sequence-substitution variance. We give explicit formulas for both components. We then analyze the asymptotic regime as the number of loci grows large; we show that METAL distances concetrate around expectations, and we study the spectrum of the limiting covariance matrix. The spectral analysis suggests that much of the phylogenetic signal lies in the first few principal components of the distance matrix when the number of leaves is large.

Our simulation study illustrates how the relative performance of distance-based methods depends on the underlying source of variation. In particular, METAL tends to outperform GLASS when the substitutional noise dominates, while GLASS becomes more accurate as coalescence variance takes over. This sensitivity reflects the design of each estimator; METAL effectively aggregates substitutional uncertainty across loci, whereas GLASS leverages gene-tree precision when it is available. Likewise, our results show METAL retaining strong performance even under rate variation and short branches. Finally, we introduce a new Gaussian-sampling procedure to estimate METAL split-support values, which uses the derived covariance to quantify support for inferred clades.

There are several natural extensions of our work. Our current analysis assumed a strict molecular clock, so that all lineages evolve at the same rate and that the gene and species trees are equidistant. Relaxing this assumption is important for real data and would allow us to combine our modular method with other phylogenetic inference schemes such as balanced minimum evolution [24]. Generalizing our covariance derivation to non-clock settings would be a major next step of our current analysis. Similarly, there is value in exploring other choices of distance, such as logDet distances [6], which may reduce bias in estimation. Moreover, although we derived the expected four-point gap of a quartet (i.e., the signal) in Sect. “Expected gap”, we have not yet considered the corresponding signal-to-noise ratio. Quantifying this ratio could provide insight into the likelihood of correctly reconstructing the species tree, as a higher signal relative to noise should generally increase reconstruction accuracy.

In conclusion, we have offered a systematic decomposition of the covariance structure in distance-based methods like METAL and STEAC, separating the contributions of gene-tree (coalescent) variability from sequence-level (substitutional) noise. Our framework not only illuminates how these two sources of uncertainty compare to each other, but also lays the groundwork for extending this analysis to a broader class of estimators. An important direction for future research is to determine the extent to which our findings on the balance between coalescent and substitutional effects hold under alternative models and estimators. Addressing this question will deepen our understanding of estimator performance across diverse biological scenarios.

## Figures and Tables

**Fig. 10:**
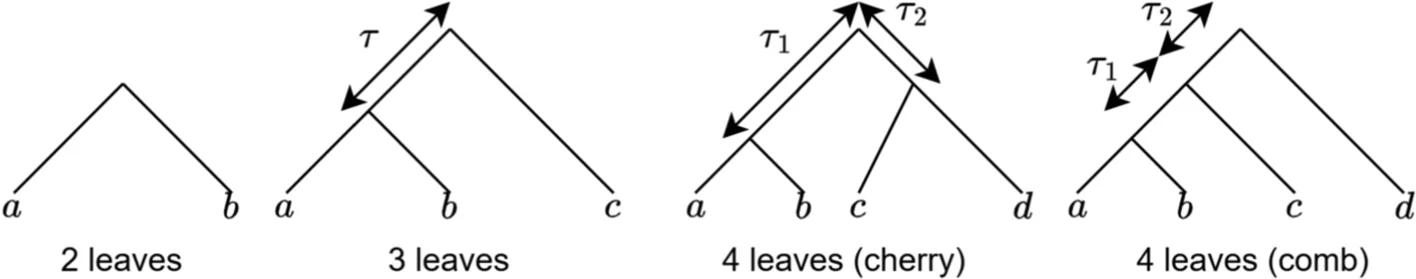
The four possible shapes of tree containing *a,b,c,d ∈ L* with $a\ne b, c\ne d$

**Fig. 11:**
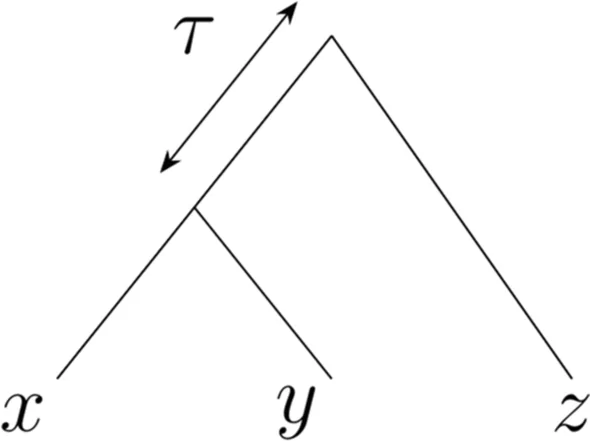
Three-leaf tree $\mathcal {A}_{\tau }$ with internal branch length *τ *. Note that $A_{0} = *$

**Fig. 12:**
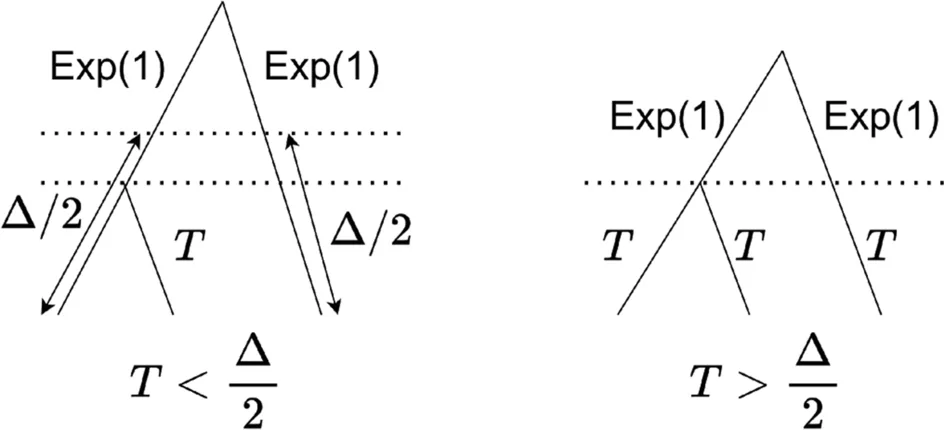
The total length of this gene tree is $T+2 \frac{\Delta }{2} + 2{{\,\textrm{Exp}\,}}(1)$ if *T < Δ / 2* (left) and $3T + 2{{\,\textrm{Exp}\,}}(1)$ if $T \ge \frac{\Delta }{2}$ (right)

**Algorithm 1:**
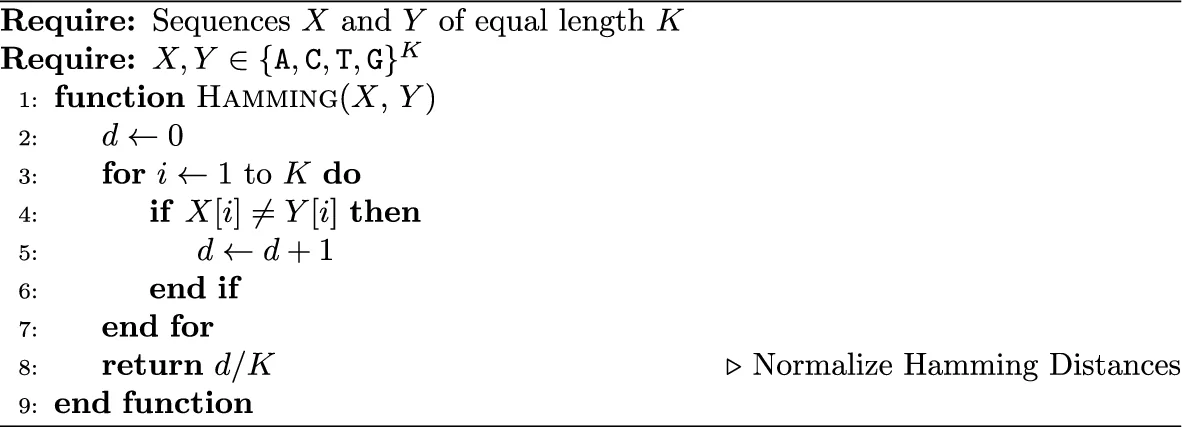
Hamming Distance Between Two Sequences

**Algorithm 2:**
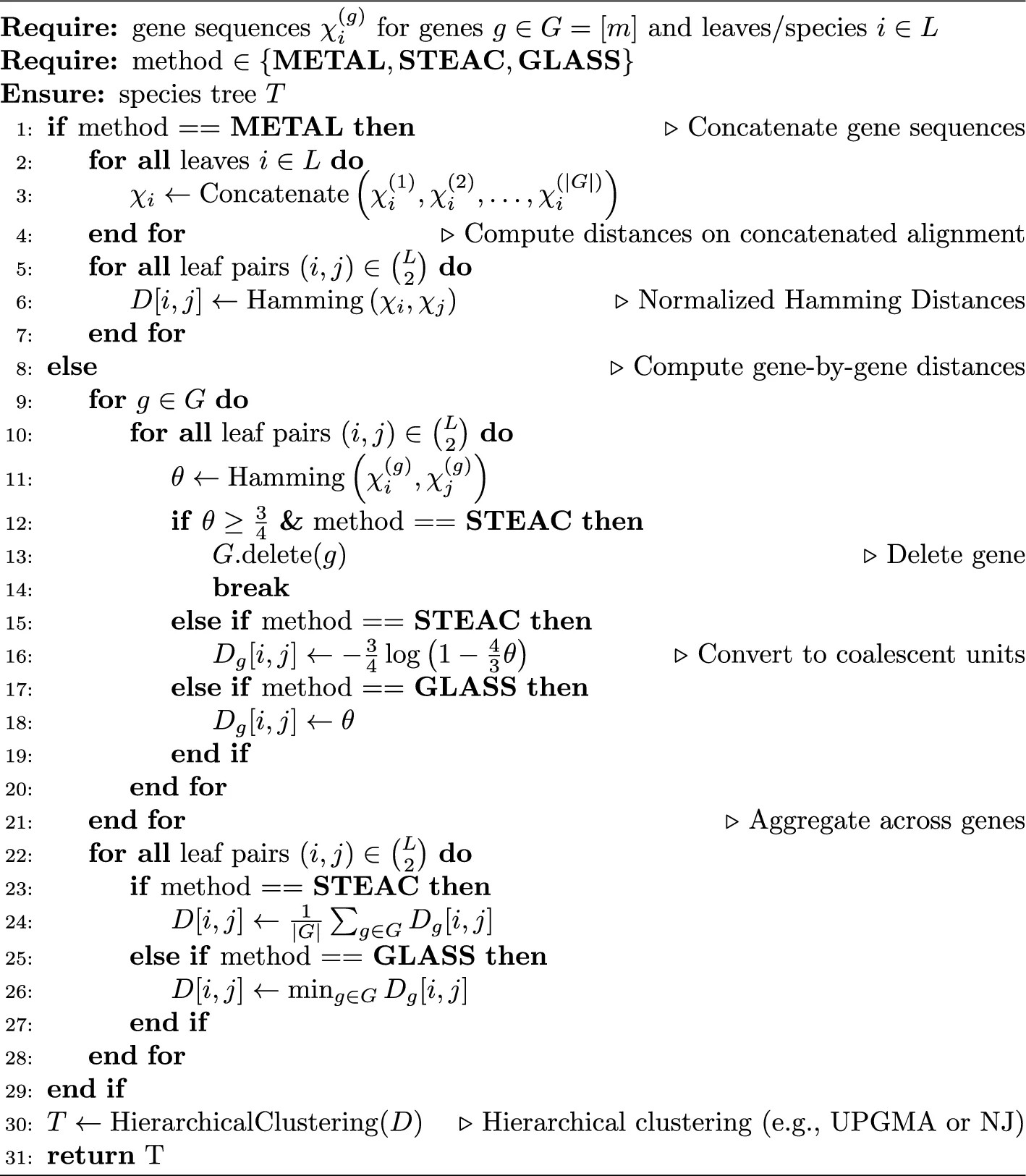
Species-Tree Estimation via Agglomerative Distance-Based Methods

**Fig. 13:**
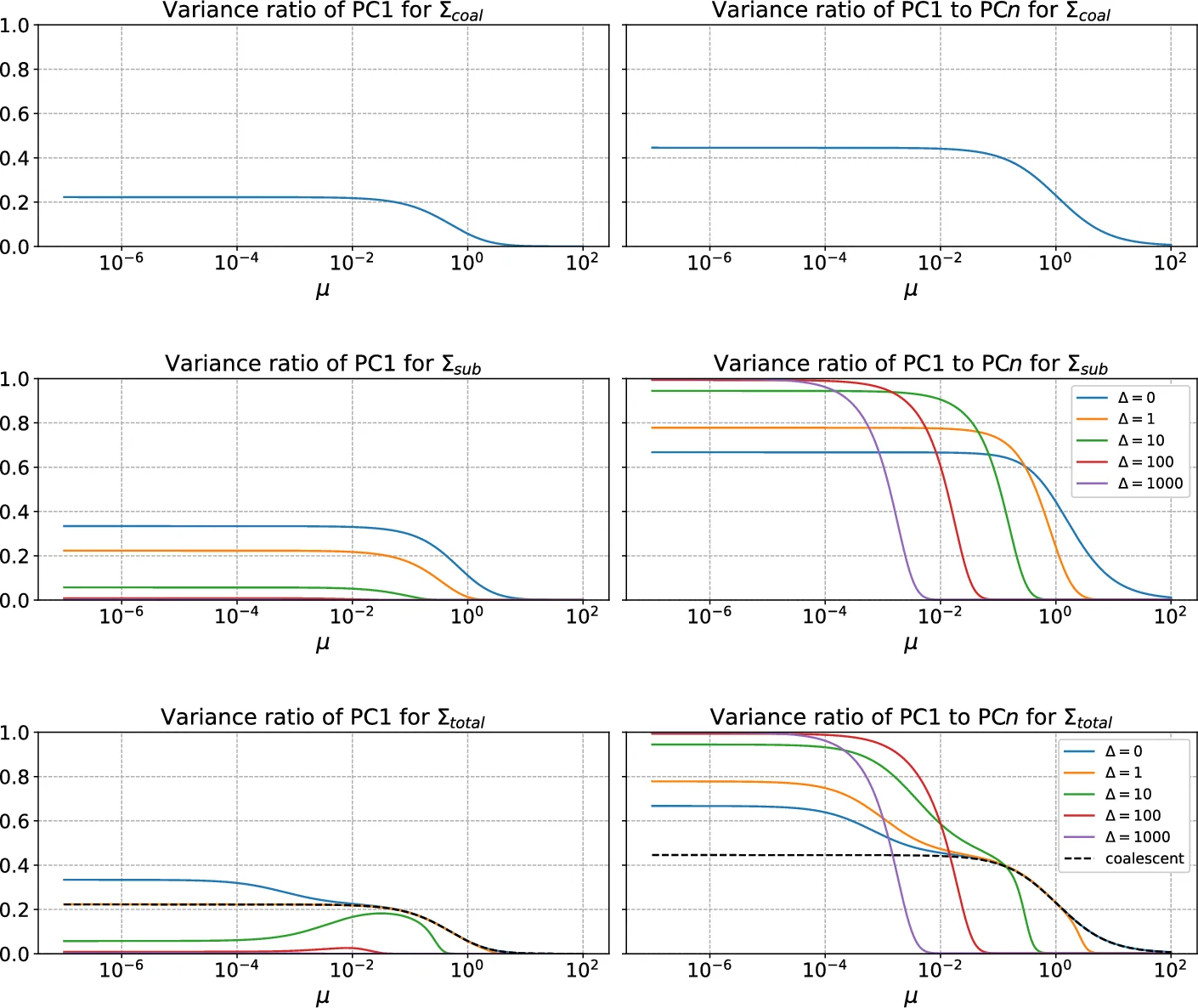
Variance ratios for (top) $\Sigma _\textrm{coal}$, (middle) $\Sigma _\textrm{sub}$, and (bottom) $\Sigma _\textrm{total}$, plotted against mutation rate *μ * and species-tree diameter *Δ *. In each row, the left panel shows the fraction of total variance explained by the first principal component, and the right panel shows the fraction of total variance explained by the first *n* principal components. Here, the number of sites per gene is *K=1000*. Since $\Sigma _\textrm{coal}$ does not depend on *Δ *, its row contains only a single curve. For $\Sigma _\textrm{total}$, note that when $\mu \ll 1$ and $\mu \Delta \gg 1$ (i.e., substitution variance dominates), its curves resemble those of $\Sigma _\textrm{sub}$, whereas for intermediate *μ * (when MSC variance dominates), all three models’ curves converge to that of the coalescent covariance

**Fig. 15:**
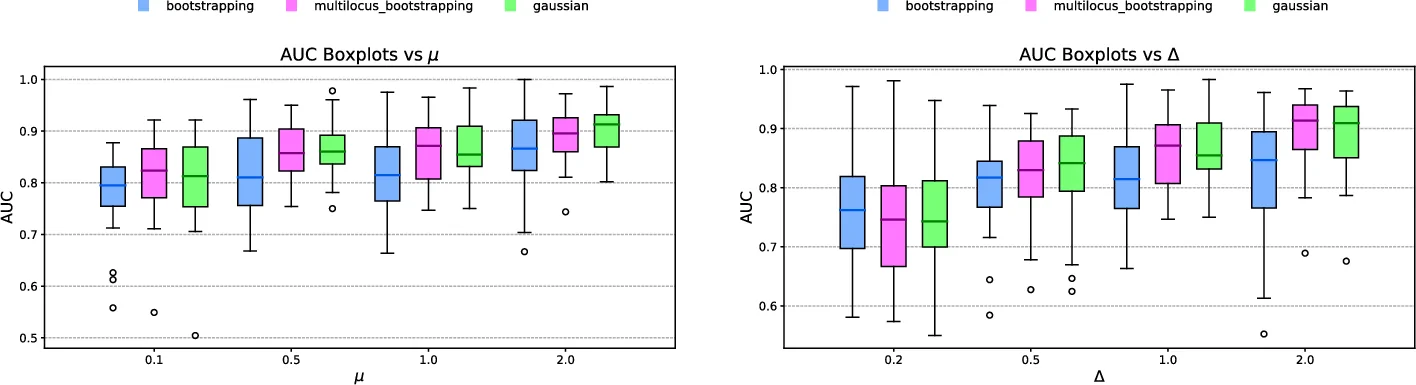
Boxplots of AUC values for different mutation rates *μ * (left), and different species tree diameter *Δ * (right)

**Fig. 16:**
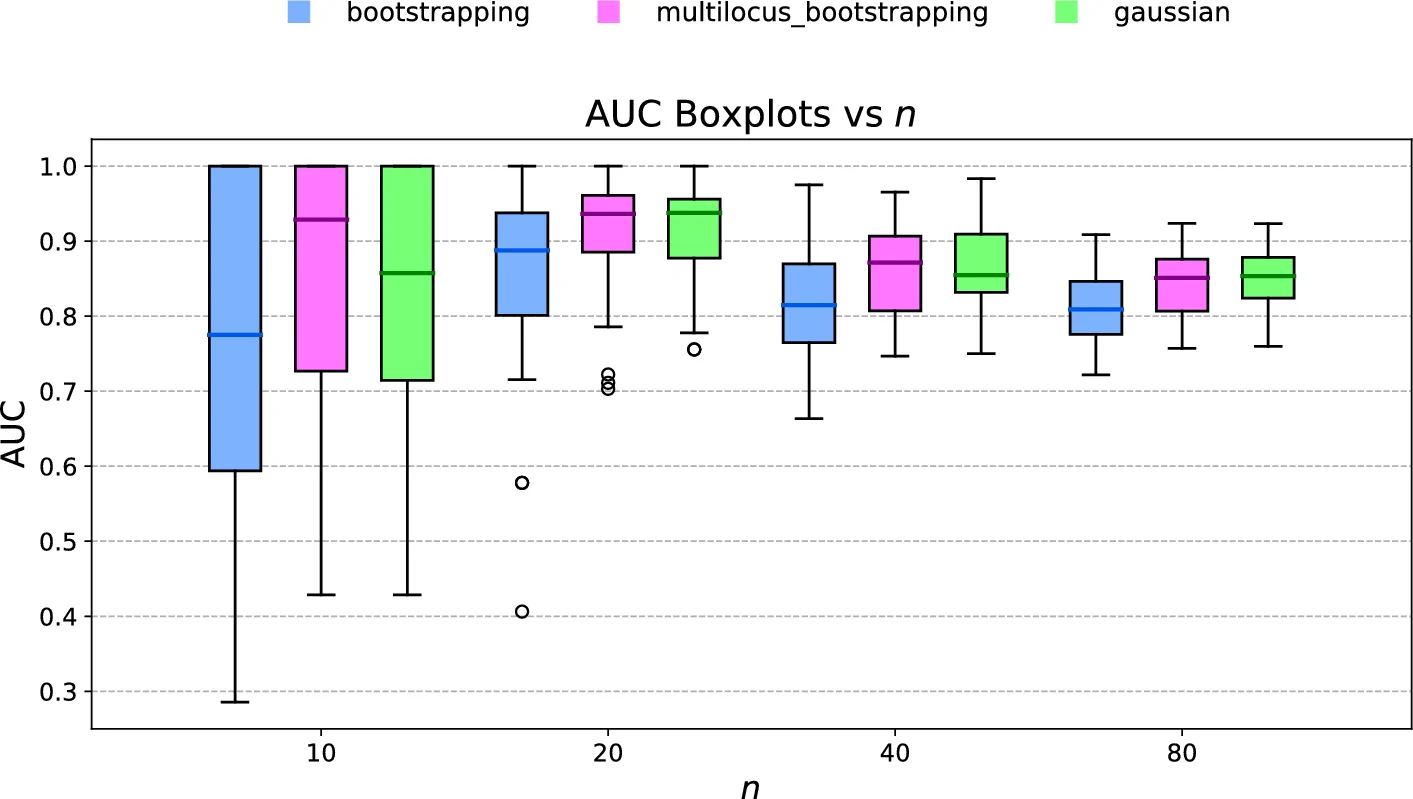
Boxplots of AUC values for different number of taxa *n*

## Data Availability

DRYAD with DOI: https://doi.org/10.5061/dryad.tht76hf65

## Appendix A Proofs

### **Proof of Proposition 1**

The definitions of the dissimilarity metrics can be found in Equations (1)–(3). We start by considering low mutation rates. Since,

$$\begin{aligned} \lim _{\mu \rightarrow 0}\frac{3}{4} \cdot \frac{1 - \exp \left( - \frac{4}{3} \mu g_{ab}^{(i)} \right) }{\mu } = g_{ab}^{(i)},\,\, \forall a,b \in L \end{aligned}$$

and by expressing Eq. (3) as the partial sum of terms $1 - \exp \left( - \frac{4}{3} \mu g_{ab}^{(i)} \right)$, it follows that

$$\begin{aligned} \lim _{\mu \rightarrow 0} \frac{d^\mathrm{(METAL)}(a,b)}{\mu } = \frac{1}{m} \sum _{i=1}^{m} g_{ab}^{(i)} = d^\mathrm{(STEAC)}(a,b), \,\, \forall a,b \in L. \end{aligned}$$

Therefore, in the limit $\mu \rightarrow 0$, the pairwise METAL distances become proportional to the STEAC distances. Since hierarchical clustering dendrograms are invariant under scalar multiplication of the distance matrix,

$$\begin{aligned} \lim _{\mu \rightarrow 0} \hat{T}_\textrm{METAL}(\mu ) = \hat{T}_\textrm{STEAC}. \end{aligned}$$

For high mutation rates, note that if the GLASS minimum coalescence times are unique, i.e. for any given pair of leaves $a,b \in L, a \ne b$ there exists $i^* \in [m]$ such that $\min _{j \in [m]} g_{ab}^{(j)} = g_{ab}^{(i^*)} < g_{ab}^{(i)}$ for all $i \in [m]\backslash \{i^*\}$, then

$$\begin{aligned} \lim _{\mu \rightarrow \infty } m \frac{1- \frac{4}{3}d^\mathrm{(METAL)}(a,b)}{\exp \left( - \frac{4}{3} \mu d^\mathrm{(GLASS)}(a,b) \right) } = \lim _{\mu \rightarrow \infty } \sum _{i=1}^m \exp \left( -\frac{4}{3} \mu \left( g_{ab}^{(i)}-g_{ab}^{(i^*)} \right) \right) = 1. \end{aligned}$$

In other words,

$$\begin{aligned} d^\mathrm{(METAL)}(a,b) = \frac{3}{4} \left( 1- \left( \frac{1}{m} + o(1) \right) \exp \left( - \frac{4}{3} \mu d^\mathrm{(GLASS)}(a,b) \right) \right) . \end{aligned}$$

Note that $f(x) = \frac{3}{4} \left( 1 - a \exp \left( - \frac{4}{3} \mu x \right) \right)$ is a strictly increasing function. Hence, for a sufficiently large *μ *,

$$\begin{aligned} d^\mathrm{(GLASS)}(a,b)< d^\mathrm{(GLASS)}(c,d) \Rightarrow d^\mathrm{(METAL)}(a,b) < d^\mathrm{(METAL)}(c,d). \end{aligned}$$

Since the number of combinations *a,b,c,d ∈ L* is finite, there exists a sufficiently large *μ * such that the above equation holds for all *a,b,c,d ∈ L*. Hence, the asymptotic ordering of METAL distances agrees with the order of GLASS distances. Another way of reaching the same conclusion is using the monotone admissibility of hierarchical clustering methods such as single linkage or complete linkage [25], which allows monotonic transformation of the elements of the distance matrix without altering the clustering. *□ *

### **Proof of Proposition 2**

Instead of writing $$g_{ab}^{(1)}$$ to refer to locus 1 specifically, we write $$g_{ab} $$ for the remainder of the proof. First, we address the trivial case where *a=b* or *c=d*. Assume that wlog *a=b*, then $$g_{ab}= 0$$ and $$e^{t g_{ab}} = 1$$, which implies that

$$ \textrm{Cov}(e^{tg_{aa}}) = \textrm{Cov}(g_{aa}) = 0, $$

which implies that the corresponding correlations will be zero too, validating the results immediately in this case.

For the rest of the proof, assume that *a ≠ b* and *c ≠ d*.

There are four cases to consider regarding the choice of the two pairs.

- **Two leaves only**, where wlog *a=c* and *b=d*.
- **Three leaves only**, where wlog *d=a* and $S_{ab} \le S_{ac} = S_{bc}$.
- **Four leaves forming a cherry tree**, where $S_{ab} \le S_{cd} \le S_{ad} = S_{bd} = S_{ac} = S_{bc}$.
- **Four leaves forming a comb tree**, where $S_{ab} \le S_{ac} = S_{bc} \le S_{ad} = S_{bd} = S_{cd}$.

These are the only cases to be considered; if *a*, *b*, *c*, *d* do not satisfy them, they can always be permuted so that at least one of the conditions above are satisfied.

Fig. 10 illustrates those four cases.

**Two leaf tree**   For the case where *(a,b) = (c,d)*, the covariance expressions becomes variances, which are positive. Under the MSC $g_{ab} = S_{ab} + 2 E_1$, where $E_1 \sim {{\,\textrm{Exp}\,}}(1)$, and so

A1 $$\begin{aligned} \textrm{Cov}(g_{ab},g_{ab}) = {{\,\textrm{Var}\,}}(g_{ab})&= 4,\end{aligned}$$

A2 $$\begin{aligned} \textrm{Cov}(e^{t g_{ab} },e^{t g_{ab} }) = {{\,\textrm{Var}\,}}(e^{t g_{ab} })&= \mathbb {E}\left( e^{2t g_{ab}} \right) - \left( \mathbb {E}( e^{t g_{ab}}) \right) ^2 \nonumber \\ &= e^{2tS_{ab}} \mathbb {E}\left( e^{2t E_1 } \right) - e^{2tS_{ab}} \mathbb {E}\left( e^{t E_1 } \right) ^2 \nonumber \\ &= \frac{4t^2e^{2tS_{ab}}}{(1-4t)(1-2t)^2}, \end{aligned}$$

where for the last result we use that the moment generating function (MGF) of ${{\,\textrm{Exp}\,}}(1)$ is $M(t) = \mathbb {E}\left( e^{t E_1 } \right) = 1/(1-t)$ and $\mathbb {E}\left( e^{2t E_1 } \right) = 1/(1-2t)$.

For the remaining three cases, we consider the variable

$$ E_{vw,xy} = \frac{ \left( g_{vw} + g_{xy} - S_{vw} - S_{xy} \right) }{2}, $$

where * v, w, x, y ∈ L *, and its moment-generating function (MGF)

$$ M_{vw,xy}(t) = \mathbb {E}\left( \exp \left( t E_{vw,xy} \right) \right) . $$

The moment-generating function allows access to the covariances of interest, as the two auxiliary results below (Eqs. (A3),(A4)) show. We now show these results.

For each pair *(i,j)*, define

$$ Y_{ij} := \frac{g_{ij}-S_{ij}}{2} \sim \textrm{Exp}(1), $$

with $\mathbb {E}[Y_{ij}]=1$, $\textrm{Var}(Y_{ij})=1$ and hence $\mathbb {E}[Y_{ij}^2]=2$. Writing

$$ E_{vw,xy} = Y_{vw}+Y_{xy}, $$

we have

$$ \frac{\partial ^2M_{vw,xy}}{\partial t^2}(0) = \mathbb {E}\bigl [(Y_{vw}+Y_{xy})^2\bigr ] = \mathbb {E}[Y_{vw}^2] + \mathbb {E}[Y_{xy}^2] + 2\,\mathbb {E}[Y_{vw}Y_{xy}]. $$

But,

$$ \mathbb {E}[Y_{vw}Y_{xy}] = \textrm{Cov}(Y_{vw},Y_{xy}) + \mathbb {E}[Y_{vw}]\;\mathbb {E}[Y_{xy}] = \textrm{Cov}(Y_{vw},Y_{xy}) + 1. $$

Therefore

$$ \frac{\partial ^2M_{vw,xy}}{\partial t^2}(0) = 2 + 2 + 2\bigl (\textrm{Cov}(Y_{vw},Y_{xy}) + 1\bigr ) = 6 + 2\,\textrm{Cov}(Y_{vw},Y_{xy}). $$

Noting that $\textrm{Cov}(Y_{vw},Y_{xy})=\tfrac{1}{4}\,\textrm{Cov}(g_{vw},g_{xy})$ recovers

A3 $$\begin{aligned} \frac{\partial ^2M_{vw,xy}}{\partial t^2}(0) =6+\tfrac{1}{2}\,\textrm{Cov}\bigl (g_{vw},g_{xy}\bigr ). \end{aligned}$$

Similarly, one shows that

A4 $$\begin{aligned} \textrm{Cov}\bigl (e^{t g_{vw}},e^{t g_{xy}}\bigr ) = \Bigl (M_{vw,xy}(2t)-\tfrac{1}{(1-2t)^2}\Bigr )\,e^{t(S_{vw}+S_{xy})}. \end{aligned}$$

Indeed, since $g_{ij}=S_{ij}+2Y_{ij}$ with $Y_{ij}\sim {{\,\textrm{Exp}\,}}(1)$,

$$ \mathbb {E}[e^{t g_{vw}}e^{t g_{xy}}] = e^{t(S_{vw}+S_{xy})}\,\mathbb {E}[e^{2t(Y_{vw}+Y_{xy})}] = e^{t(S_{vw}+S_{xy})}\,M_{vw,xy}(2t), $$

and

$$ \mathbb {E}[e^{t g_{ij}}] = e^{tS_{ij}}\,(1-2t)^{-1}, $$

where $1/(1-2t)^2$ is the MGF of $2{{\,\textrm{Exp}\,}}(1)$, so $\mathbb {E}[e^{t g_{vw}}]\, \mathbb {E}[e^{t g_{xy}}] = e^{t(S_{vw}+S_{xy})}(1-2t)^{-2}$. Subtracting gives (A4).

**Three leaf tree** Consider the case of a three-leaf tree where $S_{ab} \le S_{ac} = S_{bc}$, where $\tau = \frac{S_{ac} - S_{ab}}{2}$ as shown in the second leftmost tree in Fig. 10. Define the joint random variable $(T,\textbf{p})$, where *T* is the first time that a pair coalesces and $\textbf{p}$ is that pair. In other words,

$$\begin{aligned} T&= \min _{x,y \in \left( {\begin{array}{c}\{a,b,c\}\\ 2\end{array}}\right) } \{g_{xy}\}, \\ \textbf{p}&= \mathop {\mathrm {arg\,min}}\limits _{(x,y) \in \left( {\begin{array}{c}\{a,b,c\}\\ 2\end{array}}\right) } \{g_{xy}\}. \end{aligned}$$

Since the first speciation event happens at time $S_{ab}/2$, we require that $T \ge S_{ab}/2$. If $T \in (S_{ab}/2, S_{ac}/2)$, then $\textbf{p}=(a,b)$. The joint distribution function of $(T,\textbf{p})$ is

$$\begin{aligned} f_{T,\textbf{p}}(T,\textbf{p}) = {\left\{ \begin{array}{ll} \exp \left( -\left( T-\frac{S_{ab}}{2}\right) \right) , & \textrm{if} \,\, T \in \left( \frac{S_{ab}}{2}, \frac{S_{ac}}{2} \right) , \textbf{p} = (a,b) \\ \exp (-\tau ) \exp \left( - 3 \left( T-\frac{S_{ac}}{2} \right) \right) , & \textrm{if} \,\, T \ge \frac{S_{ac}}{2}, \, \forall \textbf{p} \in {\{a,b,c\} \atopwithdelims ()2}, \\ 0, & \textrm{otherwise}, \end{array}\right. } \end{aligned}$$

where for the remainder of this proof $$\left( {\begin{array}{c}\mathcal {A}\\ 2\end{array}}\right) $$ is the set containing pairs of distinct elements of the set $\mathcal {A}$.

Note that $T-\frac{S_{ac}}{2}|T>\frac{S_{ac}}{2} \sim {{\,\textrm{Exp}\,}}(3)$, but since there are three choices of pairs, the pdf of ${{\,\textrm{Exp}\,}}(3)$ is divided by 3 for each pair in the joint pdf above. Moreover, the conditioning probability $\mathbb {P}(T>\frac{S_{ac}}{2}) = \exp (-\tau )$ is included.

Conditional on $$(T,\textbf{p})$$,

$$\begin{aligned} E_{ab,ac} |(T,\textbf{p}) \overset{d}{=}\ {\left\{ \begin{array}{ll} \left( T- \frac{S_{ab}}{2} \right) + {{\,\textrm{Exp}\,}}(1), & \textrm{if} \,\, T \in \left( \frac{S_{ab}}{2}, \frac{S_{ac}}{2} \right) , \textbf{p} = (a,b) \\ 2\left( T-\frac{S_{ac}}{2} \right) + \tau + 2 {{\,\textrm{Exp}\,}}(1), & \textrm{if} \,\, T \ge \frac{S_{ac}}{2}, \textbf{p} = (b,c) \\ 2\left( T-\frac{S_{ac}}{2} \right) + \tau + {{\,\textrm{Exp}\,}}(1), & \textrm{if} \,\, T \ge \frac{S_{ac}}{2}, \textbf{p} = (a,b) \,\textrm{or}\, (a,c) \\ \end{array}\right. } \end{aligned}$$

and so

$$\begin{aligned} \mathbb {E}\left( e^{t E_{ab,ac}}|(T,\textbf{p})\right) = {\left\{ \begin{array}{ll} \frac{\exp \left( t \left( T- \frac{S_{ab}}{2}\right) \right) }{1-t}, & \textrm{if} \,\, T \in \left( \frac{S_{ab}}{2}, \frac{S_{ac}}{2} \right) , \textbf{p} = (a,b) \\ \frac{\exp {\left( 2t \left( T - \frac{S_{ac}}{2} \right) + t \tau \right) }}{1-2t}, & \textrm{if} \,\, T \ge \frac{S_{ac}}{2}, \textbf{p} = (b,c) \\ \frac{\exp {\left( 2t \left( T - \frac{S_{ac}}{2} \right) + t \tau \right) }}{1-t}, & \textrm{if} \,\, T \ge \frac{S_{ac}}{2}, \textbf{p} = (a,b) \,\textrm{or}\, (a,c) \\ \end{array}\right. } \end{aligned}$$

Finally, combining those two results gives us the MGF,

$$\begin{aligned} M_{ab,ac}(t)&= \mathbb {E}_T \left( \mathbb {E}( \exp (t E_{ab,ac} ) | T \right) \\ &= \int _{\frac{S_{ab}}{2}}^{\infty } \sum _{\textbf{p} \in \{(a,b),(b,c),(a,c)\}} f_{T,\textbf{p}}(T,\textbf{p}) \mathbb {E}\left( e^{t E_{ab,ac}|(T,\textbf{p})} \right) \, dT \\&= \int _{S_{ab}/2}^{S_{ac}/2} e^{ -\left( T- \frac{S_{ab}}{2}\right) } \frac{e^{t \left( T- \frac{S_{ab}}{2}\right) }}{1-t} \, dT \\ &\,\,\,\,\,\, + \int _{S_{ac}/2}^{+\infty } e^{-\tau } e^{\left( - 3 \left( T-\frac{S_{ac}}{2} \right) \right) } \frac{e^{\left( 2t \left( T - \frac{S_{ac}}{2} \right) + t \tau \right) }}{1-2t} \, dT \,\,\,\,\,\,\,\,\,\,\,\, (\textrm{for } \,\, \textbf{p} = (b,c)) \\ &\,\,\,\,\,\, + \int _{S_{ac}/2}^{+\infty } 2e^{-\tau } e^{\left( - 3 \left( T-\frac{S_{ac}}{2} \right) \right) } \frac{e^{\left( 2t \left( T - \frac{S_{ac}}{2} \right) + t \tau \right) }}{1-t} \, dT \,\,\,\,\,\,\,\,\, (\textrm{for } \,\, \textbf{p} \ne (b,c)) \\&= \frac{1}{(1-t)^2} + e^{(t-1)\tau } \frac{t^2}{(1-t)^2(1-2t)(3-2t) }. \end{aligned}$$

Taking the second derivative of this expression with respect to *t* at *t=0* yields

$$\begin{aligned} M_{ab,cd}''(0) = 6 + \frac{2}{3} e^{-\tau }, \end{aligned}$$

and so, using Eqs. (A3), and (A4), it follows that

$$\begin{aligned} \textrm{Cov}(g_{ab},g_{ac})&= \textrm{Cov}(g_{ab},g_{bc}) = \frac{4}{3} e^{-\tau } \\ \textrm{Cov}(e^{tg_{ab}},e^{tg_{ac}})&= \textrm{Cov}(e^{tg_{ab}},e^{tg_{bc}}) = e^{-\tau + 2tS_{ac} } \frac{4t^2}{(1-2t)^2 (1-4t) (3-4t)} \end{aligned}$$

which are all positive. Note that the reason these results hold for the pairs (*a*, *b*) and (*b*, *c*) is that $M_{ab,bc}(t) = M_{ab,ac}(t)$, since *a* and *b* are interchangeable. To get the correlations, we need the expression of the variances from Eqs. (A1), (A2), which yield

A5 $$\begin{aligned} \textrm{Cor}(g_{ab},g_{ac})&= \textrm{Cor}(g_{ab},g_{bc}) = \frac{1}{3} e^{-\tau } \end{aligned}$$

A6 $$\begin{aligned} \textrm{Cor}(e^{tg_{ab}},e^{tg_{ac}})&= \textrm{Cor}(e^{tg_{ab}},e^{tg_{bc}}) = \frac{e^{(2t-1)\tau }}{3-4t}, \end{aligned}$$

We also need to examine $M_{ac,bc}(t)$ which is distinct from the other two cases. Here,

$$\begin{aligned} E_{ac,bc} |(T,\textbf{p}) \overset{d}{=}\ {\left\{ \begin{array}{ll} 2 {{\,\textrm{Exp}\,}}(1), & \textrm{if} \,\, T \in \left( \frac{S_{ab}}{2}, \frac{S_{ac}}{2} \right) , \textbf{p} = (a,b) \\ 2\left( T-\frac{S_{ac}}{2} \right) + 2 {{\,\textrm{Exp}\,}}(1), & \textrm{if} \,\, T \ge \frac{S_{ac}}{2}, \textbf{p} = (a,b) \\ 2\left( T-\frac{S_{ac}}{2} \right) + {{\,\textrm{Exp}\,}}(1), & \textrm{if} \,\, T \ge \frac{S_{ac}}{2}, \textbf{p} \ne (a,b). \\ \end{array}\right. } \end{aligned}$$

Following the same approach as before,

$$\begin{aligned} M_{ac,bc}(t)&= \int _{S_{ab}/2}^{S_{ac}/2} e^{ -\left( T- \frac{S_{ab}}{2}\right) } \frac{1}{1-2t} \, dT \\ &\quad +\int _{S_{ac}/2}^{+\infty } e^{-\tau } e^{\left( - 3 \left( T-\frac{S_{ac}}{2} \right) \right) } \frac{e^{2t \left( T - \frac{S_{ac}}{2} \right) }}{1-2t} \, dT \,\,\,\,\, (\textrm{for } \,\, \textbf{p} = (a,b)) \\ &\quad + 2\int _{S_{ac}/2}^{+\infty } e^{-\tau } e^{\left( - 3 \left( T-\frac{S_{ac}}{2} \right) \right) } \frac{e^{2t \left( T - \frac{S_{ac}}{2} \right) }}{1-t} \, dT \,\,\,\,\, (\textrm{for } \,\, \textbf{p} \ne (a,b)) \\&= \frac{1}{1-2t} - e^{-\tau } \frac{2t^2}{(1-t)(1-2t)(3-2t) }. \end{aligned}$$

Hence, it follows that

A7 $$\begin{aligned} \textrm{Cov}(g_{ac},g_{bc})&= 4 - \frac{8}{3} e^{-\tau } \ge 0 \end{aligned}$$

A8 $$\begin{aligned} \textrm{Cov}(e^{tg_{ac}},e^{tg_{bc}})&= \frac{4t^2}{(1-2t) (1-4t)} e^{2tS_{ac}} \left( \frac{1}{1-2t}- e^{-\tau } \frac{2}{3-4t} \right) \end{aligned}$$

A9 $$\begin{aligned}&\ge e^{2tS_{ac}} \frac{4t^2}{(1-2t)^2 (1-4t)(3-4t)} \ge 0 \nonumber \\ \textrm{Cor}(g_{ac},g_{bc})&= 1- \frac{2}{3} e^{-\tau } \end{aligned}$$

A10 $$\begin{aligned} \textrm{Cor}(e^{tg_{ab}},e^{tg_{ac}})&= 1- \frac{2(1-2t)}{3-4t}e^{-\tau }. \end{aligned}$$

**Cherry tree** We continue by considering the cherry tree as shown in Fig. 10, with $S_{ab} \le S_{cd} \le S_{ad} = S_{bd} = S_{ac} = S_{bc} = \Delta$, where *Δ * is the diameter of the subtree containing *a*, *b*, *c*, *d*. We use this for notational convenience for the remainder of the proof and should not be confused with the diameter of the species tree contatining all leaves. We also defined, $\tau _1 = \frac{\Delta -S_{ab}}{2}$ and $\tau _2 = \frac{\Delta -S_{cd}}{2} \le \tau _1$. Once again, define the joint random variable $(T,\textbf{p})$ as before. For the cherry tree case, it has density function

$$\begin{aligned} f_{T,\textbf{p}}(T,\textbf{p}) = {\left\{ \begin{array}{ll} \exp \left( -\left( T-\frac{S_{ab}}{2}\right) \right) , & \textrm{if} \,\, T \in \left( \frac{S_{ab}}{2}, \frac{S_{ac}}{2} \right) , \textbf{p} = (a,b) \\ \exp (- (\tau _1-\tau _2)) \exp \left( -2\left( T-\frac{S_{ac}}{2}\right) \right) , & \textrm{if} \,\, T \in \left( \frac{S_{ac}}{2}, \frac{\Delta }{2} \right) , \textbf{p} = (a,b) \,\textrm{or }\, (c,d) \\ \exp (-\tau _1 - \tau _2)) \exp \left( - 6 \left( T-\frac{S_{ac}}{2} \right) \right) , & \textrm{if} \,\, T \ge \frac{\Delta }{2} \, \forall \textbf{p} \in {\{a,b,c,d\} \atopwithdelims ()2} \\ 0, & \mathrm{otherwise.} \end{array}\right. } \end{aligned}$$

Conditional on $(T,\textbf{p})$ and the species tree *S*,

$$\begin{aligned} E_{ab, cd} |(T,\textbf{p}), S \overset{d}{=}\ {\left\{ \begin{array}{ll} \left( T-\frac{S_{ab}}{2}\right) + {{\,\textrm{Exp}\,}}(1), & \textrm{if} \,\, T \in \left( \frac{S_{ab}}{2}, \frac{S_{cd}}{2} \right) , \textbf{p} = (a,b) \\ 2\left( T-\frac{S_{cd}}{2} \right) + \tau _1 - \tau _2+ {{\,\textrm{Exp}\,}}(1), & \textrm{if} \,\, T \in (\frac{S_{cd}}{2}, \frac{\Delta }{2}), \textbf{p} = (a,b), (c,d) \\ 2\left( T-\frac{\Delta }{2} \right) + \tau _1 + \tau _2 + {{\,\textrm{Exp}\,}}(1), & \textrm{if} \,\, T \ge \frac{\Delta }{2}, \textbf{p} = (a,b), (c,d) \\ 2\left( T-\frac{\Delta }{2} \right) + \tau _1 + \tau _2 + E_{xy,xz}|S=*, & \textrm{if} \,\, T \ge \frac{\Delta }{2}, \textbf{p} \ne (a,b), (c,d). \end{array}\right. } \end{aligned}$$

Note the last case where the first pair to coalesce is neither (*a*, *b*) nor (*a*, *c*). Let’s assume without loss of generality that the first pair was (*a*, *c*). Since species *a* and *c* have coalesced, we can treat them as one leaf *a=c* in a gene tree of 3 leaves *a=c, b,d* that has not been resolved yet. This gene tree is generated by the Multispecies coalescent model assuming that the species tree is the star tree, denoted *S=**, since all three species can coalesce after $T\ge \Delta /2$. Therefore,

$$ E_{ab, cd} |(T,\textbf{p}) \overset{d}{=}\ 2(T-\Delta /2) + \tau _1 + \tau _2 + E_{ab,ad}|S=*. $$

We have already computed the MGF of *E* for three-leaf trees. When *S=**, the internal branch length vanishes i.e. *τ = 0*. Hence,

$$ \mathbb {E}\left( e^{t (E_{xy,xz}|S=* )}\right) = \frac{1}{(1-t)^2} + \frac{t^2}{(1-t)^2(1-2t)(3-2t) } = \frac{3-5t}{(1-t)(1-2t)(3-2t)}, \textrm{and} $$

$$\begin{aligned} \mathbb {E}\left( e^{t E_{ab,cd}}|(T,\textbf{p})\right) = {\left\{ \begin{array}{ll} \frac{\exp \left( t \left( T- \frac{S_{ab}}{2}\right) \right) }{1-t},& \textrm{if} \,\, T \in \left( \frac{S_{ab}}{2}, \frac{S_{cd}}{2} \right) , \textbf{p} = (a,b) \\ \frac{\exp {\left( 2t \left( T - \frac{S_{cd}}{2} \right) + t (\tau _1 - \tau _2 ) \right) }}{1-t}, & \textrm{if} \,\, T \in \left( \frac{S_{ac}}{2}, \frac{\Delta }{2} \right) , \textbf{p} = (a,b) \,\textrm{or }\, (c,d) \\ \frac{\exp {\left( 2t \left( T - \frac{\Delta }{2} \right) + t (\tau _1+\tau _2) \right) }}{1-t}, & \textrm{if} \,\, T \ge \frac{\Delta }{2}, \textbf{p} = (a,b) \,\textrm{or }\, (c,d) \\ \frac{\exp {\left( 2t \left( T - \frac{\Delta }{2} \right) + t (\tau _1+\tau _2) \right) } (3-5t) }{(1-t)(1-2t)(3-2t)}, & \textrm{if} \,\, T \ge \frac{\Delta }{2}, \textbf{p} \ne (a,b) \,\textrm{or }\, (c,d). \end{array}\right. } \end{aligned}$$

Hence,

$$\begin{aligned} M_{ab,cd}(t)&= \int _{S_{ab}/2}^{S_{ac}/2} e^{ -\left( T- \frac{S_{ab}}{2}\right) } \frac{e^{ t\left( T-\frac{S_{ab}}{2} \right) }}{1-t} \, dT \\ &\quad + 2 \int _{S_{ac}/2}^{\Delta /2} e^{-(\tau _1 - \tau _2 )} e^{\left( - 2 \left( T-\frac{S_{ac}}{2} \right) \right) } \frac{e^{2t \left( T - \frac{S_{ac}}{2} \right) } e^{ t(\tau _1 - \tau _2) } }{1-2t} \, dT \\ &\quad + 2\int _{\Delta /2}^{+\infty } e^{-\tau _1 - \tau _2} e^{\left( - 6 \left( T-\frac{\Delta }{2} \right) \right) } \frac{e^{2t \left( T - \frac{\Delta }{2} \right) }e^{ t(\tau _1 + \tau _2) }}{1-t} \, dT \,\,\,\,\,\,\,\,\,\,\,\,\,\,\,\,\,\,\,\,\,\,\,\,\,\, (\textrm{for } \,\, \textbf{p} = (a,b), (c,d)) \\ &\quad + 4\int _{\Delta /2}^{+\infty } e^{-\tau _1 - \tau _2} e^{\left( - 6 \left( T-\frac{\Delta }{2} \right) \right) } \frac{e^{2t \left( T - \frac{\Delta }{2} \right) }e^{ t(\tau _1 + \tau _2)}(3-5t)}{(1-t)(1-2t)(3-2t)} \, dT \,\,\,\,\,\, (\textrm{for } \,\, \textbf{p} \ne (a,b), (c,d) ) \\&= \frac{1}{(1-t)^2} + e^{-\tau _1 - \tau _2} \frac{2t^2}{(1-t)^2(1-2t)(3-2t)(3-t) }. \end{aligned}$$

From this we conclude that

A11 $$\begin{aligned} \textrm{Cov}(g_{ab},g_{cd})&= \frac{8}{9} e^{-(\tau _1+\tau _2)} \ge 0 \end{aligned}$$

A12 $$\begin{aligned} \textrm{Cov}(e^{tg_{ab}},e^{tg_{cd}})&= e^{t(S_{ab}+S_{cd}) - (\tau _1 + \tau _2)} \frac{8t^2}{(1-2t)^2(1-4t)(3-4t)(3-2t) } \end{aligned}$$

A13 $$\begin{aligned} \textrm{Cor}(g_{ac},g_{bc})&= \frac{2}{9} e^{-(\tau _1+\tau _2)} \end{aligned}$$

A14 $$\begin{aligned} \textrm{Cor}(e^{tg_{ab}},e^{tg_{ac}})&= e^{-(\tau _1+\tau _2)} \frac{2}{(3-4t)(3-2t)}. \end{aligned}$$

A different approach is used for finding the MGF of $E_{ac,bd}$. Define the random variable $N \in \{0,1,2\}$ to be the number of pairs that coalesce before time *Δ /2*. The only pairs that could coalesce in that time interval are $\textbf{p} = (a,b)$ or (*c*, *d*) and the probabilities of each one of these pairs coalescing is independent of the other. Therefore,

$$\begin{aligned} \mathbb {P}(N=0)&= e^{-\tau _1 - \tau _2}, \\ \mathbb {P}(N=1)&= (1-e^{-\tau _1}) e^{-\tau _2} + (1-e^{-\tau _2}) e^{-\tau _1}, \\ \mathbb {P}(N=2)&= (1-e^{-\tau _1}) (1-e^{-\tau _2}). \end{aligned}$$

If *N=0*, then $E_{ac,bd} = E_{xy,zw} | S=*$ i.e. the MGF of $E_{ac,bd}$ will be the same as $M_{ab,cd}(t)$ with $\tau _1 = \tau _2=0$ or

$$\begin{aligned} \mathbb {E}\left( e^{tE_{ac,bd}}|N=0\right) = \frac{1}{(1-t)^2} + \frac{2t^2}{(1-t)^2(1-2t)(3-2t)(3-t) }. \end{aligned}$$

If *N=1*, either (*a*, *b*) or (*c*, *d*) have coalesced before time *Δ /2* but not both, and so at time *Δ /2* there will be three nodes that have not coalesced yet. Consequently, $E_{ac,bd} = E_{xy,xw} | S=*$ i.e. the MGF of $E_{ac,bd}$ will be the same as $M_{ab,ac}(t)$ with *τ = 0* or

$$\begin{aligned} \mathbb {E}\left( e^{tE_{ac,bd}}|N=1\right) = \frac{1}{(1-t)^2} + \frac{t^2}{(1-t)^2(1-2t)(3-2t)}. \end{aligned}$$

For *N=2*, both (*a*, *b*) and (*c*, *d*) have coalesced, $E_{ac,bd} = 2{{\,\textrm{Exp}\,}}(1)$, and thus

$$\begin{aligned} \mathbb {E}\left( e^{tE_{ac,bd}}|N=2\right) = \frac{1}{1-2t} = \frac{1}{(1-t)^2} + \frac{t^2}{(1-t)^2 (1-2t)}. \end{aligned}$$

Combining those three results,

$$\begin{aligned} M_{ac,bd}(t)&= \mathbb {E}_N(\mathbb {E}(e^{tE_{ac,bd}}|N) ) = \sum _{n=0}^{2} \mathbb {E}(e^{tE_{ac,bd}}|N=n) \mathbb {P}(N=n)\\ &= \frac{1}{(1-t)^2} + \frac{2t^2 e^{-\tau _1-\tau _2}}{(1-t)^2(1-2t)(3-2t)(3-t) } \\ &\quad + \frac{t^2 \left( (1-e^{-\tau _1}) e^{-\tau _2} + (1-e^{-\tau _2}) e^{-\tau _1}\right) }{(1-t)^2(1-2t)(3-2t)} + \frac{t^2 (1-e^{-\tau _1}) (1-e^{-\tau _2})}{(1-t)^2 (1-2t)}. \end{aligned}$$

The same argument can be made for the pair (*a*, *d*) and (*b*, *c*), since *a*, *b* are interchangeable. We conclude that

A15 $$\begin{aligned} \textrm{Cor}(g_{ad},g_{bc}) = \textrm{Cor}(g_{ac},g_{bd})&= 1 - \frac{2}{3} \left( e^{-\tau _1} + e^{-\tau _2} \right) + \frac{5}{9} e^{-(\tau _1+\tau _2)} \end{aligned}$$

A16 $$\begin{aligned} \textrm{Cor}(e^{tg_{ad}},e^{tg_{bc}}) = \textrm{Cor}(e^{tg_{ac}},e^{tg_{bd}})&= 1- \frac{2(1-2t)}{3-4t} (e^{-\tau _1} + e^{-\tau _2})\nonumber \\&\quad +e^{-(\tau _1+\tau _2)} \frac{(5-4t)(1-2t)}{(3-4t) (3-2t)}. \end{aligned}$$

We now need to prove that the correlations in Eqs. (A15) and (A16) are non-negative and that the correlation of $\textrm{Cor}(e^{tg_{ac}},e^{tg_{bd}})$ is an increasing function of $t \in (-\infty , 0]$. Note that since,

$$\begin{aligned} \lim _{t \rightarrow - \infty } \textrm{Cor}(e^{tg_{ad}},e^{tg_{bc}})&= (1-e^{-\tau _1}) ( 1 - e^{-\tau _2} ) \ge 0, \, \textrm{and} \\ \lim _{t \rightarrow 0 } \textrm{Cor}(e^{tg_{ad}},e^{tg_{bc}})&= \textrm{Cor}(g_{ad},g_{bc}), \end{aligned}$$

proving the monotonicity of $\textrm{Cor}(e^{tg_{ac}},e^{tg_{bd}})$ on *(-∞ , 0]* is sufficient for the positivity of (A15) and (A16).

To do that, we compute the derivative,

$$\begin{aligned} \frac{d(\textrm{Cor}(e^{tg_{ac}},e^{tg_{bd}}))}{dt}&= \frac{4}{(3-4t)^2} \left( (e^{-\tau _1} + e^{-\tau _2}) - \frac{8t^2 - 16t + 9}{(3-2t)^2} e^{-\tau _1-\tau _2} \right) \\ &\ge \frac{4}{(3-4t)^2} \left( (e^{-\tau _1} + e^{-\tau _2}) - 2 e^{-\tau _1-\tau _2} \right) \ge 0 . \end{aligned}$$

The first inequality comes from the observation that

$$\begin{aligned} \frac{8t^2 - 16t + 9}{(3-2t)^2} \ge 2 ~~\Leftrightarrow ~~ t < \frac{9}{8}. \end{aligned}$$

The second inequality stems from the fact that the function

$$\begin{aligned} g:[0,1]^2 \rightarrow \mathbb {R},\, g(x,y)=x+y-2xy \end{aligned}$$

has non-negative range, which can be proved by rewriting *g* as

$$ g(x,y) = 1/2 - \left( x-\frac{1}{2} \right) \left( 2y-1 \right) , $$

which is clearly minimized at *x,y = (1,1)*, and so $\min _{(x,y) \in [0,1]^2} g(x,y) = g(1,1) = 0$. This concludes the proof for the case of the cherry tree.

**Comb tree** Finally, we conclude this proof with the last tree shape of Fig. 10, the comb tree, where $S_{ab} \le S_{ac} = S_{bc} \le S_{ad} = S_{bd} = S_{cd}= \Delta$ and $\tau _1 = \frac{S_{ac} - S_{ab}}{2}, \, \tau _2 = \frac{\Delta - S_{ac}}{2}$.

The joint density function of $(T,\textbf{p})$ is

$$\begin{aligned} f_{T,\textbf{p}}(T,\textbf{p}) = {\left\{ \begin{array}{ll} \exp \left( -\left( T-\frac{S_{ab}}{2}\right) \right) , & \textrm{if} \,\, T \in \left( \frac{S_{ab}}{2}, \frac{S_{ac}}{2} \right) , \textbf{p} = (a,b) \\ \exp (- \tau _1) \exp \left( -3\left( T-\frac{S_{ac}}{2}\right) \right) , & \textrm{if} \,\, T \in \left( \frac{S_{ac}}{2}, \frac{\Delta }{2} \right) , \forall \textbf{p} \in {\{a,b,c\} \atopwithdelims ()2} \\ \exp (-\tau _1 - 3\tau _2)) \exp \left( - 6 \left( T-\frac{\Delta }{2} \right) \right) , & \textrm{if} \,\, T \ge \frac{\Delta }{2} \, \forall \textbf{p} \in {\{a,b,c,d\} \atopwithdelims ()2} \\ 0, & \mathrm{otherwise.} \end{array}\right. } \end{aligned}$$

Furthermore,

$$\begin{aligned} E_{ab, cd} |(T,\textbf{p}), S \overset{d}{=}\ {\left\{ \begin{array}{ll} \left( T-\frac{S_{ab}}{2}\right) + {{\,\textrm{Exp}\,}}(1), & \textrm{if} \,\, T \in \left( \frac{S_{ab}}{2}, \frac{S_{ac}}{2} \right) , \textbf{p} = (a,b) \\ \left( T-\frac{S_{ab}}{2}\right) + {{\,\textrm{Exp}\,}}(1), & \textrm{if} \,\, T \in (\frac{S_{ac}}{2}, \frac{\Delta }{2}), \textbf{p} = (a,b) \\ \left( T-\frac{S_{ab}}{2}\right) + E_{xy,xz} | S=\mathcal {A}_{\Delta /2 - T}, & \textrm{if} \,\, T \in (\frac{S_{ac}}{2}, \frac{\Delta }{2}), \textbf{p} = (a,c), (b,c)\\ 2\left( T-\frac{\Delta }{2} \right) + \tau _1 + \tau _2 + {{\,\textrm{Exp}\,}}(1), & \textrm{if} \,\, T \ge \frac{\Delta }{2}, \textbf{p} = (a,b), (c,d) \\ 2\left( T-\frac{\Delta }{2} \right) + \tau _1 + \tau _2 + E_{xy,xz}|S=*, & \textrm{if} \,\, T \ge \frac{\Delta }{2}, \textbf{p} \ne (a,b), (c,d). \end{array}\right. } \end{aligned}$$

where the three-leaf tree $\mathcal {A}_{\tau }$, for *τ >0*, is drawn in Fig. 11.

The MGF of $E_{xy,xz} | S=\mathcal {A}_{\tau }$ and $E_{xy,xz} | S=*$ has already been computed before (three-leaf tree) as

$$\begin{aligned} M_{xy,xz|\mathcal {A}_{\tau }}(t)&= \frac{1}{(1-t)^2} + e^{(t-1)\tau } \frac{t^2}{(1-t)^2(1-2t)(3-2t) }, \\ M_{xy,xz|*}(t)&= M_{xy,xz|\mathcal {A}_{0}}(t) = \frac{3-5t}{(1-t)(1-2t)(3-2t)}. \end{aligned}$$

Hence,

$$\begin{aligned} M_{ab,cd}(t)&= \int _{S_{ab}/2}^{S_{ac}/2} e^{ -\left( T- \frac{S_{ab}}{2}\right) } \frac{e^{ t\left( T-\frac{S_{ab}}{2} \right) }}{1-t} \, dT \\ &\quad + \int _{S_{ac}/2}^{\Delta /2} e^{-\tau _1} e^{\left( - 3 \left( T-\frac{S_{ac}}{2} \right) \right) } \frac{e^{t \left( T - \frac{S_{ac}}{2} \right) } e^{ t\tau _1 } }{1-t} \, dT \,\,\,\,\, (\textrm{for } \,\, \textbf{p} = (a,b))\\&\quad+ \int _{S_{ac}/2}^{\Delta /2} e^{-\tau _1} e^{\left( - 3 \left( T-\frac{S_{ac}}{2} \right) \right) } e^{2t \left( T - \frac{S_{ac}}{2} \right) } e^{ t\tau _1 } M_{xy,xz| \mathcal {A}_{\Delta /2-T}}(t) \, dT \,\,\,\,\, (\textrm{for } \,\, \textbf{p} = (a,c),(b,c))\\&\quad +2\int _{\Delta /2}^{+\infty } e^{-\tau _1 - 3\tau _2} e^{\left( - 6 \left( T-\frac{\Delta }{2} \right) \right) } \frac{e^{2t \left( T - \frac{\Delta }{2} \right) }e^{ t(\tau _1 + \tau _2) }}{1-t} \, dT \,\,\,\,\, (\textrm{for } \,\, \textbf{p} = (a,b), (c,d)) \\ &\quad +4\int _{\Delta /2}^{+\infty } e^{-\tau _1 - 3\tau _2} e^{\left( - 6 \left( T-\frac{\Delta }{2} \right) \right) } e^{2t \left( T - \frac{\Delta }{2} \right) }e^{ t(\tau _1 + \tau _2)} M_{xy,xz|*}(t) \, dT \,\,\,\,\, (\textrm{for } \,\, \textbf{p} \ne (a,b), (c,d) ) \\&= \frac{1}{(1-t)^2}+ e^{(t-1) (\tau _1+ \tau _2)} \frac{t^2 \left( (3-t) - (1-t) e^{-2 \tau _2} \right) }{(1-t)^2(1-2t)(3-2t)(3-t)}, \end{aligned}$$

and so

A17 $$\begin{aligned} \textrm{Cor}(g_{ab},g_{cd})&=\frac{1}{3} e^{-\tau _1-\tau _2} - \frac{1}{9} e^{-\tau _1 - 3\tau _2} \in \left( 0, \frac{1}{3} \right) , \end{aligned}$$

A18 $$\begin{aligned} \textrm{Cor}(e^{tg_{ab}},e^{tg_{cd}})&= \frac{e^{(2t-1) (\tau _1+\tau _2)}}{3-4t} \left( 1 - \frac{1-2t}{3-2t} e^{-2\tau _2} \right) . \end{aligned}$$

Note that $\textrm{Cor}(e^{tg_{ab}},e^{tg_{cd}})$ is an increasing function of *t* on *(-∞ ,0)*, since the first term is increasing and $- \frac{1-2t}{3-2t}$ is also increasing.

$$\begin{aligned} E_{ac, bd} |(T,\textbf{p}), S \overset{d}{=}\ {\left\{ \begin{array}{ll} E_{xy,xz} | S = \mathcal {A}_{\tau _2}, & \textrm{if} \,\, T \in \left( \frac{S_{ab}}{2}, \frac{S_{ac}}{2} \right) , \textbf{p} = (a,b) \\ E_{ab,cd}|(T,\textbf{p}), S, & \textrm{if} \,\, T \ge \frac{S_{ac}}{2}. \end{array}\right. } \end{aligned}$$

The last case follows from the fact that *a*, *b*, *c* are interchangeable conditional on (*a*, *b*) not coalescing before time $S_{ac}/2$. Moreover, note that $E_{ab,cd}|T > S_{ac}/2, S$ has MGF equal to the above $M_{ab,cd}(t)$, but with $\tau _1=0$ i.e.

$$\begin{aligned} M_{ab,cd|\tau _1=0}(t) = \frac{1}{(1-t)^2}+ e^{(t-1) \tau _2} \frac{t^2 \left( (3-t) - (1-t) e^{-2 \tau _2} \right) }{(1-t)^2(1-2t)(3-2t)(3-t)}. \end{aligned}$$

Hence, it follows that

$$\begin{aligned} M_{ac,bd}(t)&= \left( 1-e^{-\tau _1}\right) M_{xy,xz|\mathcal {A}_{\tau _2}}(t) + e^{-\tau _2} M_{ab,cd|\tau _1=0}(t) \\&= \frac{1}{(1-t)^2} + \frac{t^2}{(1-t)^2(1-2t)(3-2t)} e^{(t-1)\tau _2} \left( 1 - e^{-2\tau _2-\tau _1} \frac{1-t}{3-t} \right) . \end{aligned}$$

Also note that $M_{ac,bd}(t) = M_{ad,bc}(t)$. It follows that the correlations are as follows

A19 $$\begin{aligned} \textrm{Cor}(g_{ac},g_{bd}) = \textrm{Cor}(g_{ad},g_{bc})&=\frac{1}{3} e^{-\tau _2} - \frac{1}{9} e^{-\tau _1 - 3\tau _2} \in \left( 0, \frac{1}{3} \right) , \end{aligned}$$

A20 $$\begin{aligned} \textrm{Cor}(e^{tg_{ac}},e^{tg_{bd}}) = \textrm{Cor}(e^{tg_{ad}},e^{tg_{bc}})&= \frac{e^{(2t-1) \tau _2}}{3-4t} \left( 1 - \frac{1-2t}{3-2t} e^{-2\tau _2- \tau _1} \right) . \end{aligned}$$

Similarly, the correlation is an increasing function of *t*. This concludes the proof for the case of the comb tree. *□ *

### **Proof of Proposition 3**

The covariance of interest can be rewritten as follows, using the properties of indicator random variables,

A21 $$\begin{aligned} \textrm{Cov}( 1\!\!1( \chi _a \ne \chi _b ), 1\!\!1( \chi _c \ne \chi _d ) )&= \textrm{Cov}( 1\!\!1( \chi _a = \chi _b ), 1\!\!1( \chi _c = \chi _d ) ) \nonumber \\ \&= \mathbb {P}(\chi _a = \chi _b, \chi _c = \chi _d) - \mathbb {P}(\chi _a = \chi _b) \mathbb {P}(\chi _c = \chi _d). \end{aligned}$$

Under the Jukes-Cantor evolutionary model, when there is a substitution, it is equally likely that the current base is substituted by any one of the four bases A, C, T, G. Since the probability of no substitution across a branch of time length *t* is $\exp (-\mu t)$, the probability that a randomly selected base stays the same across the same branch is

$$\exp (-\mu t) + \frac{1}{4}(1-\exp (-\mu t)) = \frac{1}{4} + \frac{3}{4} \exp (-\mu t).$$

It follows that,

A22 $$\begin{aligned} \mathbb {P}(\chi _i = \chi _j) = \frac{3}{4} e^{-\mu g_{ij}} + \frac{1}{4}, \, \forall i,j \in L. \end{aligned}$$

The second term of Eq. (A21) can then be computed through a product of terms computed from Eq. (A22). We now focus on the first term of Eq. (A21).

Let *r* and *q* be the internal nodes connecting *a* and *c* and *b* and *d* respectively, such that the length of

the path from *q* to *r* is equal to $\delta _{ab,cd}$. Similarly to Eq. (A22),

A23 $$\begin{aligned} \mathbb {P}(\chi _r = \chi _q) = \frac{3}{4} e^{-\mu \delta _{ab,cd} } + \frac{1}{4}. \end{aligned}$$

We replace the states *A*, *C*, *G*, *T* with numbers 0, 1, 2, 3 respectively. Define the random variable $N_{v,w} \equiv |\chi _{v} - \chi _{w}| \mod 4$ and note that

$$\begin{aligned} \{\chi _r = \chi _q\}&= \{ N_{rq} \equiv 0 \mod 4 \}, \\ \{\chi _a = \chi _b\}&= \{ N_{ab} \equiv 0 \mod 4 \}, \\ \{\chi _c = \chi _d\}&= \{ N_{cd} \equiv 0 \mod 4 \},\quad \textrm{where} \\ N_{ab}&\equiv N_{ar} + N_{rq} + N_{qb} \mod 4, \\ N_{cd}&\equiv N_{cr} + N_{rq} + N_{qd} \mod 4 \end{aligned}$$

Moreover, $N_{ar}, N_{rq}, N_{qb}, N_{cr}, N_{qd}$ are all independent of each other since their corresponding paths are disjoint. Hence, $N_{ar} + N_{qb}$ is independent of $N_{cr} + N_{qd}$. For the remainder of the proof *\mod 4* will be dropped when *≡ * is used.

Conditioning on $$N_{rq}$$,

A24 $$\begin{aligned} \mathbb {P}(\chi _a = \chi _b, \chi _c = \chi _d)&= \sum _{i=0}^3 \mathbb {P}(\chi _a = \chi _b, \chi _c = \chi _d | N_{rq} \equiv i) \mathbb {P}(N_{rq} \equiv i) \nonumber \\&= \sum _{i=0}^3 \mathbb {P}(N_{ar} + N_{qb} \equiv -i, N_{cr} + N_{qd} \equiv -i) \mathbb {P}(N_{rq} \equiv i ) \nonumber \\&= \sum _{i=0}^3 \mathbb {P}(N_{ar} + N_{qb} \equiv -i) \mathbb {P}(N_{cr} + N_{qd} \equiv -i) \mathbb {P}(N_{rq} \equiv i ) \nonumber \\ &= \mathbb {P}(N_{ar} + N_{qb} \equiv 0) \mathbb {P}(N_{cr} + N_{qd} \equiv 0) \mathbb {P}(\chi _r = \chi _q ) \nonumber \\&\quad + \frac{1}{9} \mathbb {P}(N_{ar} + N_{qb} \not \equiv 0) \mathbb {P}(N_{cr} + N_{qd} \not \equiv 0) \mathbb {P}(\chi _r \ne \chi _q ), \end{aligned}$$

where the third equality holds because of the independence of $N_{ar} + N_{qb}$ and $N_{cr} + N_{qd}$, and the fourth equality holds because all the terms in the summand are equal and each probabilities involving sums of *N* being *≡ i* are a third of the corresponding probabilities with $\not \equiv 0$.

Now the only thing left is to find the distribution of $N_{ar}+ N_{qb}$ and $N_{cr} + N_{qd}$. Without loss of generality, we will focus on the former. The probability that there are no substitutions across the paths from *a* to *r* and *q* to *b* is $\exp (-\mu (g_{ab} - \delta _{ab,cd}))$, since the length of the sum of the paths is $g_{ab} - \delta _{ab,cd}$. If there has been no substitution along those paths, then $N_{ar}+ N_{qb} \equiv 0$. Otherwise, if there has been at least one substitution, $N_{ar}+ N_{qb} \equiv i$, where *i=0,1,2,3* are all equiprobable. Therefore,

A25 $$\begin{aligned} \mathbb {P}(N_{ar}+ N_{qb} \equiv 0)&= \frac{1}{4} + \frac{3}{4} e^{-\mu (g_{ab} - \delta _{ab,cd})} \end{aligned}$$

A26 $$\begin{aligned} \mathbb {P}(N_{cr} + N_{qd} \equiv 0)&= \frac{1}{4} + \frac{3}{4} e^{-\mu (g_{cd} - \delta _{ab,cd})} \end{aligned}$$

Substituting Equations (A25)–(A26) and Eq. (A23) into Eq. (A24) gives

$$\begin{aligned} \mathbb {P}(\chi _a = \chi _b, \chi _c = \chi _d)&= \frac{1}{16} \left( 1 + 3e^{-\mu (g_{ab}+g_{cd} - 2\delta _{ab,cd})} + 3e^{-\mu g_{ab}} \right. \\ &\left. \quad + 3 e^{-\mu g_{cd}} + 6e^{-\mu (g_{ab} + g_{cd} -\delta _{ab,cd}) } \right) \end{aligned}$$

Substituting the above to Eq. (A21) yields the desired result. *□ *

### **Proof of Proposition 4**

Under the Jukes–Cantor model, the expected estimated distance satisfies $\mathbb {E}[\hat{d}_{xy}] = 1 - \mathbb {E}(e^{-\mu g_{xy}})$, where $g_{xy}$ is the gene-tree distance between *x* and *y* and *μ * is the substitution rate. Under the multispecies coalescent (MSC), we may write $g_{xy} = S_{xy} + 2E_{xy}$, where $S_{xy}$ is the species-tree path length and $E_{xy} \sim \textrm{Exp}(1)$ is the coalescent waiting time after speciation. Hence,

$$ \mathbb {E}(e^{-\mu g_{xy}}) = e^{-\mu S_{xy}}\,\mathbb {E}\left( e^{-2\mu E_{xy}} \right) = \frac{1}{1+2\mu }\,e^{-\mu S_{xy}}. $$

The constant terms (the leading 1’s in each $\mathbb {E}[\hat{d}_{xy}] = 1 - \mathbb {E}(e^{-\mu g_{xy}})$) cancel in the four-point expression, giving

$$ \mathbb {E}\!\left( \hat{d}_{ad} + \hat{d}_{bc} - \hat{d}_{ab} - \hat{d}_{cd}\right) = \mathbb {E}(e^{-\mu g_{ab}}) + \mathbb {E}(e^{-\mu g_{cd}}) - \mathbb {E}(e^{-\mu g_{ad}}) - \mathbb {E}(e^{-\mu g_{bc}}), $$

and substituting the expectation above yields (7). For the cherry topology, $S_{ad}=S_{bc}=\Delta _{\{a,b,c,d\}}$, which gives the stated simplification. For the comb tree case with topology * (((a,b),c),d) *, $S_{cd}=S_{ad}$ and $S_{bc}=S_{ac}$, and so the expression collapses to $\tfrac{1}{1+2\mu }(e^{-\mu S_{ab}} - e^{-\mu S_{ac}})$. *□ *

### **Proof of Proposition 5**

Using the law of total covariance, the total covariance between pairs of METAL distance estimates for a single loci is

27 $$\begin{aligned}&\textrm{Cov}\left( \hat{p}_{ab}^{(1)}, \hat{p}_{cd}^{(1)} \right) \nonumber \\&= \mathbb {E}_{G^{(1)}| \mathcal {S}} \left( \textrm{Cov}\left( \hat{p}_{ab}^{(1)}, \hat{p}_{cd}^{(1)} | G^{(1)}\right) \right) + \textrm{Cov}_{G^{(1)}| \mathcal {S}} \left( \mathbb {E}\left( \hat{p}_{ab}^{(1)}|G^{(1)} \right) , \mathbb {E}\left( \hat{p}_{cd}^{(1)}|G^{(1)} \right) \right) \nonumber \\ &= \frac{3}{16 K } \mathbb {E}_{G^{(1)}| \mathcal {S}} \left( e^{-\mu \left( g_{ab}^{(i)} + g_{cd}^{(i)} \right) } \left( e^{2\mu \delta _{ab,cd}^{(i)}} + 2 e^{\mu \delta _{ab,cd}^{(i)}} - 3 \right) \right) + \frac{9}{16} \textrm{Cov}\left( e^{-\mu g_{ab}}, e^{-\mu g_{cd}}\right) . \end{aligned}$$

To derive the last line we use Proposition 3 for the first term and the Jukes-Cantor formula

$$\begin{aligned} \mathbb {E}\left( \hat{p}_{xy}^{(1)}|G^{(1)} \right) = p^{(1)}_{xy | G^{(1)}} = \frac{3}{4} \left( 1- e^{-\mu g_{xy}^{(1)} }\right) . \end{aligned}$$

for the second term. Using the definitions in Equations (8), (10), (11), Eq. (9) is equivalent to (27).

It is easy to see that $\Sigma _\textrm{coal}$ is positive semi-definite, as a covariance matrix of the random vector $$\exp {\left( -\mu g_{\left( {\begin{array}{c}L\\ 2\end{array}}\right) } \right) }$$. For $\Sigma _\textrm{sub}$, observe that

$$ \Sigma _{\hat{p}_{\left( {\begin{array}{c}L\\ 2\end{array}}\right) }^{(1)} | G^{(1)} } {:}{=}\textrm{Cov}\left( \hat{p}_{ab}^{(1)}, \hat{p}_{cd}^{(1)} | G^{(1)}\right) , $$

is positive semidefinite i.e. for any vector $$\textbf{v} \in \mathbb {R}^{\left( {\begin{array}{c}n\\ 2\end{array}}\right) }$$, $$\textbf{v} \Sigma _{\hat{p}_{\left( {\begin{array}{c}L\\ 2\end{array}}\right) }^{(1)} | G^{(1)} } \textbf{v}^T \ge 0$$ and so

$$ \textbf{v} \Sigma _\textrm{sub} \textbf{v}^T = \mathbb {E}\left( \textbf{v} \Sigma _{\hat{p}_{\left( {\begin{array}{c}L\\ 2\end{array}}\right) }^{(1)} | G^{(1)} } v^T \right) \ge 0 $$

This proves that $\Sigma _\textrm{sub}$ is a positive semi-definite matrix. *□ *

### **Proof of Proposition 6**

Using the formulas from the Proof of Proposition 2 for the coalescent covariance matrix and the formulas from Appendix B for the substitution covariance matrix. Observe that for the star species tree, the three leaf covariances agree with each other for both the coalescent and the substitution matrix. So do the results for the four leaf covariances. In other words, $(\Sigma _\textrm{coal})_{ab,cd}$ and $(\Sigma _\textrm{sub})_{ab,cd}$ only depend on the total number of leaves $|\{a,b,c,d\}| \in \{2,3,4\}$. Also, note that $|\{a,b\} \cap \{c,d\}| = 4 - |\{a,b,c,d\}| \in \{0,1,2\}$ and so $\sigma ^{(0)},\sigma ^{(1)},\sigma ^{(2)}$ now refer to 4-leaf, 3-leaf, and 2-leaf covariances respectively.

First, we derive the formulas of $\sigma _\textrm{mode}^{(i)}$ for $\textrm{mode} \in \{ \textrm{coal, sub} \}, i \in \{0,1,2\}$.

A28 $$\begin{aligned} \sigma _\textrm{coal}^{(0)}(\mu )&= \frac{8\mu ^2}{(1+4\mu )(1+2\mu )^2(3+4\mu )(3+2\mu )} e^{-2\mu \Delta } \end{aligned}$$

A29 $$\begin{aligned} \sigma _\textrm{coal}^{(1)}(\mu )&= \frac{4\mu ^2}{(1+4\mu )(1+2\mu )^2(3+4\mu )} e^{-2\mu \Delta } = \frac{3+2\mu }{2}\sigma _\textrm{coal}^{(0)}(\mu ) \end{aligned}$$

A30 $$\begin{aligned} \sigma _\textrm{coal}^{(2)}(\mu )&= \frac{4\mu ^2}{(1+4\mu )(1+2\mu )^2} e^{-2\mu \Delta } = (3+4\mu )\sigma _\textrm{coal}^{(1)}(\mu ) \end{aligned}$$

A31 $$\begin{aligned} \sigma _\textrm{sub}^{(0)}(\mu )&= \frac{e^{-2\mu \Delta }}{(1+2\mu )(3+2\mu )} - \sigma _\textrm{coal}^{(0)}(\mu ) - \frac{e^{-2\mu \Delta }}{(1+2\mu )^2} \nonumber \\&= \frac{4\mu (8\mu ^2+ 17\mu + 6)}{3(\mu +1)(2\mu +1)(2\mu +3)(4\mu +1)(4\mu +3)} e^{-2\mu \Delta } \end{aligned}$$

A32 $$\begin{aligned} \sigma _\textrm{sub}^{(1)}(\mu )&= \frac{2}{3} \frac{e^{-3\mu \Delta /2}}{(1+2\mu )(1+\mu )} + \frac{1}{3} \frac{e^{-\mu \Delta }}{1+2\mu } - \sigma _\textrm{coal}^{(1)}(\mu ) - \frac{e^{-2\mu \Delta }}{(1+2\mu )^2} \nonumber \\&\ge e^{-2\mu \Delta } \frac{2\mu (8\mu ^2 + 17\mu + 6)}{3(\mu +1)(4\mu +1)(4\mu +3)(2\mu +1)} = \frac{3+2\mu }{2}\sigma _\textrm{sub}^{(0)}(\mu ) \end{aligned}$$

A33 $$\begin{aligned} \sigma _\textrm{sub}^{(2)}(\mu )&= \frac{2}{3} \frac{e^{-\mu \Delta }}{1+2\mu } + \frac{1}{3} - \sigma _\textrm{coal}^{(2)}(\mu ) - \frac{e^{-2\mu \Delta }}{(1+2\mu )^2} \ge \frac{8}{3} \frac{\mu (\mu +1)}{(4\mu +1)(2\mu +1)} e^{-2\mu \Delta } \end{aligned}$$

The inequalities are derived by using $e^{-i \mu \Delta } \le e^{-2 \mu \Delta }$ for all *i ≤ 2*. From here, the asymptotic results as $\mu \rightarrow 0$ and $\mu \rightarrow \infty$ are easy to derive. It is also clear that $\sigma _\textrm{coal}^{(i)}$ is decreasing with $i \in \{0,1,2\}$, and that $\sigma _\textrm{sub}^{(1)}(\mu ) \ge \sigma _\textrm{sub}^{(0)}(\mu )$. We now prove that $\sigma _\textrm{sub}^{(2)}(\mu ) \ge \sigma _\textrm{sub}^{(1)}(\mu )$,

$$\begin{aligned} \sigma _\textrm{sub}^{(2)}(\mu ) - \sigma _\textrm{sub}^{(1)}(\mu )&\ge \frac{1}{3} e^{-3\mu \Delta /2} \frac{2\mu (\mu +2)}{(1+2\mu ) (1+\mu )} - 2(1 + 2\mu )\sigma _\textrm{coal}^{(1)}(\mu ) \\&\ge e^{-2\mu \Delta } \frac{2 \left( 16\mu ^3 + 36\mu ^2 + 23\mu + 6\right) }{3 ( \mu + 1 )( 2\mu + 1 )( 4\mu + 1 )( 4\mu + 3 )} >0. \end{aligned}$$

This concludes the proof. *□ *

### **Proof of Proposition 7**

1 First we find the spectrum of the following matrix;

$$\begin{aligned} A \in \mathbb {R}^{ {n \atopwithdelims ()2} \times {n \atopwithdelims ()2} }, A_{ab,cd} = |\{a,b\} \cap \{c,d\}| \in \{0,1,2\} \end{aligned}$$

We need to prove the following results for *A*

i. *r(A) ≤ n*.
ii. Take two distinct leaves *x,y ∈ [n]*. Consider the vector $v^{(xy)} \in \mathbb {R}^{n \atopwithdelims ()2}$ with $v^{(xy)}_{xy} = 2n - 4$, $v^{(xy)}_{xz} = v^{(xy)}_{yz} = n-4$ and $v^{(xy)}_{zw} = -4$ for all distinct *z,w ≠ x,y*. Then $v^{(xy)}$ is an eigenvector of *A* with eigenvalue *n-2*. In particular, the eigenvectors $v^{(12)}, v^{(13)}, \dots , v^{(1n)}$ are linearly independent and so $\dim (E_{n-2}(A) ) \ge n-1$, where $E_{\rho }(Y)$ is the eigenspace of matrix *Y* associated with eigenvalue *ρ *.

The first result shows that the dimension of the eigenspace $E_{0}(A)$ is at least ${n \atopwithdelims ()2} - n = n(n-3)/2$ by the rank-nullity theorem. The second result shows that $\dim \left( E_{n-2}(A)\right) \ge n-1$. The dominant eigenvalue is *2n - 2* (with corresponding eigenvector $\textbf{1}$) and the corresponding eigenspace has dimension at least 1. Since the sum of the dimensions of eigenspaces is *n \atopwithdelims ()2*, it follows that

$$\begin{aligned} \dim \left( E_{2n-2}(A)\right)&= 1, \\ \dim \left( E_{n-2}(A)\right)&= n-1, \\ \dim \left( E_{0}(A)\right)&= \frac{n(n-3)}{2}. \end{aligned}$$

*Proof of i.* First, observe that the sum of rows is a covariance under MSC derived $\textbf{1} = (1,\dots , 1)$, since

$$\begin{aligned} \sum _{a<b} A_{ab, cd} = \sum _{a<b} |\{a,b\} \cap \{c,d\}| = 2(n-1), \, \, \forall (c,d) \in \left( {\begin{array}{c}[n]\\ 2\end{array}}\right) . \end{aligned}$$

Hence, it suffices to prove that the space spanned by the first *n-1* rows $A_{1l,.}$ for $l \in \{2,\dots ,n\}$ along with the row *(1,… ,1)* (which is the sum of all the rows of *A*) contains all the other rows $A_{xy,.}$ for *x, y ≠ 1*. To do that, our goal is to write $A_{xy,.}$ as a linear combination of the first *n-1* rows the vector *(1,… ,1)* for any *x,y >1*, and proving element-wise that it holds. Consider the coefficients $\lambda ^{}_j = \lambda + \left| \{x,y\} \cap \{j\} \right| , j \in \{2,\dots ,n\}$, corresponding to row $A_{1j,.}$, where *λ * is a free parameter to be determined. Also let * -2 λ * be the coefficient corresponding to the newly created row $\textbf{1} = (1,1,\dots ,1)$. Then, for all components *zw* with *z ≠ w* and *z,w ∈ [n]*,

$$\begin{aligned} \sum _{j=2}^n \lambda ^{}_j A_{1j, zw} + (-2\lambda ) \textbf{1}_{zw}&= - 2 \lambda + \sum _{j=2}^n \left( \lambda + \left| \{j\} \cap \{x,y\} \right| \right) \left| \{1,j \} \cap \{z,w\} \right| \\ &= - 2 \lambda + \sum _{j=2}^n \left( \lambda + \left| \{j\} \cap \{x,y\} \right| \right) \left( \left| \{1\} \cap \{z,w\} \right| + \left| \{j\} \cap \{z,w\} \right| \right) \\ &= - 2 \lambda - 2 \lambda \left| \{1\} \cap \{z,w\} \right| \\ &\,\,\,\, + \sum _{j=1}^n \left( \lambda + \left| \{j\} \cap \{x,y\} \right| \right) \left( \left| \{1\} \cap \{z,w\} \right| + \left| \{j\} \cap \{z,w\} \right| \right) \\ &= - 2 \lambda - 2 \lambda \left| \{1\} \cap \{z,w\} \right| (\lambda n + 2) \left| \{1\} \cap \{z,w\} \right| + 2 \lambda \\ &\,\,\,\, + \sum _{j=1}^n \left| \{ j \} \cap \{z,w\} \right| \cdot \left| \{j\} \cap \{x,y\} \right| \\ &= \left| \{1\} \cap \{z,w\} \right| \left( \lambda (n-2) + 2 \right) + \left| \{z,w\} \cap \{x,y\}\right| = A_{xy,zw} \end{aligned}$$

For the last equality to hold, note that first term vanishes if we choose $\lambda = \frac{-2}{n-2}$ and the summation term is equal to $\left| \{z,w\} \cap \{x,y\}\right|$. Hence, we have proved the following result

$$\begin{aligned} \sum _{j=2}^n \lambda ^{(x,y)}_j A_{1j,.} + \lambda _1^{(x,y)} \textbf{1} = A_{xy,.}, \forall x,y \in \{ 2, 3, \dots , n \}, x \ne y \end{aligned}$$

which means that for all distinct *x,y ∈ [n]* we have $A_{xy,.} \in {{\,\textrm{span}\,}}\{ A_{12,.}, \dots , A_{1n,.}, \textbf{1} \}$. Hence, the rank of *A* cannot exceed *n*.

*Proof of ii.* We now need to verify that $A v^{(xy)} = (n-2) v^{(xy)}$. First, observe that

$$\begin{aligned} (A^2)_{ab,cd} = \sum _{x<y} A_{ab,xy} A_{xy,cd} = {\left\{ \begin{array}{ll} 2n, & \textrm{if} |\{a,b\} \cap \{c,d\} | = 2, \\ n+2, & \textrm{if} |\{a,b\} \cap \{c,d\} | = 1, \\ 4, & \textrm{if} |\{a,b\} \cap \{c,d\} | = 0 \\ \end{array}\right. } \end{aligned}$$

and so it follows that

$$\begin{aligned} A^2 = (n-2) A + 4 \cdot \textbf{1} \textbf{1}^T, \end{aligned}$$

where $\textbf{1} = (1, \dots ,1)$ is a column vector i.e. $$\textbf{1} \textbf{1}^T \in \mathbb {R}^{ \left( {\begin{array}{c}n\\ 2\end{array}}\right) \times \left( {\begin{array}{c}n\\ 2\end{array}}\right) }$$ is a matrix of all-ones. Given that $A \textbf{1} = (2n-2) \textbf{1}$, we conclude that

$$\begin{aligned} A( nA - 4 \cdot \textbf{1} \textbf{1}^T ) = (n-2) ( nA - 4 \cdot \textbf{1} \textbf{1}^T), \end{aligned}$$

which implies that the columns of $V {:}{=}nA - 4 \cdot \textbf{1} \textbf{1}^T$ are all eigenvectors of A with eigenvalue *n-2*. Note that $v^{(xy)} = V_{., xy}$ i.e. the vectors constructed in statement (ii) are precisely the columns of *V* and hence elements in the eigenspace $E_{n-2}(A)$.

To prove that $v^{(12)}, \dots , v^{(1n)}$ are linearly independent, consider the square matrix $B \in \mathbb {R}^{(n-1) \times (n-1)}$ with $B_{i,j} = v_{i}^{(1j)}$. It suffices to prove that *B* is non-singular. Note that $B_{i,i} = 2n-4> n-4 = B_{i,j} > 0$ for all *j ≠ i* and $i \in \{2,\dots ,n\}$ and denote them as $B_{ii}= x, B_{ij} = y$ for all $i \in [N], j \ne i$ where *N=n-1* and *x> y > 0*. Observing that *B* is a circulant matrix, its eigenvalues are

$$\begin{aligned} \rho _k = \sum _{j=0}^{N-1} c_j \omega ^{kj}, k \in \{0,\dots , N-1\} \end{aligned}$$

where $c_0 = x, \, c_1= \dots = c_{N-1} = y$, and *ω * is the $N^\textrm{th}$-root of unity. Hence, $\rho _0 = x+ (n-1) y > 0$ and $\rho _k = x + y \sum _{j=1}^{N-1} \omega ^{kj} = x-y > 0$ for $k \in \{1,\dots ,N-1\}$. All eigenvalues are positive and so *B* is non-singular. Having found the spectrum of *A*, the task is to now find the spectrum of *C*. Observe that

$$ C = (\beta - \gamma ) A + ( \gamma + \alpha - 2\beta ) I + \gamma \textbf{1}, $$

where $\textbf{1}$ is a matrix whose entries are all 1.

Every eigenvalue and eigenvector that is not $\textbf{1}$ is transformed

$$\begin{aligned} \tilde{ \lambda }&= (\beta - \gamma ) \lambda + (\gamma + \alpha - 2\beta ) \\ \tilde{v}&= v - \textbf{1} \frac{\gamma v^T \textbf{1}}{ (\beta -\gamma ) (2n-2-\lambda ) + \gamma n} \end{aligned}$$

In particular, if *Av = λ v*, then $C \tilde{v} = \tilde{\lambda } \tilde{v}$. This argument doesn’t work for $v = \textbf{1}$ since $\tilde{v} = 0$. But it’s straightforward to compute the eigenvalue directly

$$\begin{aligned} C\textbf{1} = \left( (\beta - \gamma ) (2n-2) + \gamma + \alpha - 2 \beta + {n \atopwithdelims ()2} \gamma \right) \textbf{1} \end{aligned}$$

The eigenvalue *n-2* of *A* becomes $(\beta -\gamma ) (n-2) + \gamma +\alpha - 2\beta$ and the corresponding eigenvectors do not change because $v^T \textbf{1} = 0$ for all the vectors in $E_{n-2}(A)$. The remaining eigenvalue is $\gamma +\alpha - 2\beta$. *□ *

### Proof of Proposition 7

For the trace and Frobenius norm, the results of Propositions 6 suffice to prove them. For the determinant, the spectral norm, and the results about variance ratios, the spectrum of those matrices is given by Proposition 7.

Regarding the variance ratio, note that Proposition 7 provides limits of variance ratios as $n \rightarrow \infty$. The variance ratio of PC1 is $\gamma /\alpha$ and the variance variance of the first *n* prinipal components is $\frac{2\beta -\gamma }{\alpha }$. For the covariance matrix $\Sigma _\textrm{mode}$ where mode is either coal or sub, $\alpha = \sigma _\textrm{mode}^{(2)}, \beta = \sigma _\textrm{mode}^{(1)}, \gamma = \sigma _\textrm{mode}^{(0)}$.

We start with $\Sigma _\textrm{coal}$, whose components $\alpha ,\beta ,\gamma$ are given by Equations (A28)–(A30). As $\mu \rightarrow 0$,

$$\begin{aligned} \frac{\sigma _\textrm{coal}^{(i) }}{\mu ^2} \rightarrow {\left\{ \begin{array}{ll} 4, & \textrm{if } \quad i=2\\ \frac{4}{3}, & \textrm{if } \quad i=1\\ \frac{8}{9}, & \textrm{if } \quad i=0 \end{array}\right. }. \end{aligned}$$

From that it follows that

$$\begin{aligned} \lim _{\mu \rightarrow 0} \frac{\gamma _\textrm{coal}}{\alpha _\textrm{coal}}&= \frac{2}{9}\\ \lim _{\mu \rightarrow 0} \frac{2\beta _\textrm{coal}-\gamma _\textrm{coal}}{\alpha _\textrm{coal}}&= \frac{4}{9} \end{aligned}$$

Similarly, for $\Sigma _\textrm{sub}$, as $\mu \rightarrow 0$,

$$\begin{aligned} \frac{\sigma _\textrm{sub}^{(i) }}{\mu } \rightarrow {\left\{ \begin{array}{ll} \frac{8}{3} + \frac{4}{3}\Delta , & \textrm{if } \quad i=2\\ \frac{4}{3}+\frac{2}{3}\Delta , & \textrm{if } \quad i=1\\ \frac{8}{9}, & \textrm{if } \quad i=0 \end{array}\right. }. \end{aligned}$$

From that it follows that

$$\begin{aligned} \lim _{\mu \rightarrow 0} \frac{\gamma _\textrm{sub}}{\alpha _\textrm{sub}}&= \frac{2}{6+3\Delta }\\ \lim _{\mu \rightarrow 0} \frac{2\beta _\textrm{sub}-\gamma _\textrm{sub}}{\alpha _\textrm{sub}}&= \frac{4+3\Delta }{6+3\Delta } \end{aligned}$$

This concludes the proof of the Corollary. *□ *

### **Proof of Proposition 8**

Two statements need to be proved; first, that $v^{(i)}$ is an eigenvector of $C^{\infty }_\textrm{coal}$ for all interior nodes *i*, and second, that the remaining eigenvalues are zero. Observe that $C^{\infty }_\textrm{coal}$ only takes values $\{0,1\}$. The value of $\left( C^{\infty }_\textrm{coal}\right) _{ab,cd}$ is 1 if and only if the topology of the leaves $\{a,b,c,d\}$ is either ( (*a*, *c*), (*b*, *d*) ) or ( (*a*, *d*), (*b*, *c*) ). Hence,

$$\begin{aligned} \left( C^{\infty }_\textrm{coal} v^{(i)} \right) _{ab}&= \sum _{cd} \mathbb {I}\left( ( (a,d), (b,c) ) \text { or } ( (a,c), (b,d) ) \right) \cdot \mathbb {I}\left( (c,d) \in (L_i \times R_i) \right) \\ &= \sum _{c \in L_i, d \in R_i} \mathbb {I}\left( ( (a,d), (b,c) ) \text { or } ( (a,c), (b,d) ) \right) \\ &= \sum _{c \in L_i, d \in R_i} \mathbb {I}\left( (a,d), (b,c) \right) + \sum _{c \in L_i, d \in R_i} \mathbb {I}\left( (a,c), (b,d) \right) \\ &= \mathbb {I}\left( (a,b) \in R_i \times L_i \right) |L_i| |R_i|+ \mathbb {I}\left( (a,b) \in L_i \times R_i \right) |L_i| |R_i| \\ &= |L_i| |R_i| \mathbb {I}\left( (a,b) \in (L_i \times R_i) \cup (R_i \times L_i) \right) \\ &= |L_i| |R_i| v^{(i)}_{ab} \end{aligned}$$

To prove the second statement, it suffices to prove that $\sum _{i} |L_i| |R_i| = \text {tr}(C^{\infty }_\textrm{coal})$ where the sum is over all internal nodes; that’s because the trace is the sum of eigenvalues and $C^{\infty }_\textrm{coal}$ is a symmetric semi-positive definite matrix. The diagonal of $C^{\infty }_\textrm{coal}$ only includes ones, and so $\text {tr}(C^{\infty }_\textrm{coal}) = {m \atopwithdelims ()2}$. Let *r* be the root of the tree with $|L_r| + |R_r| = m$. Denote $\xi _{\mathcal {A}}$ be the sum of eigenvalues (corresponding to interior nodes) for the tree formed from the leaf set $\mathcal {A} \subset [m]$. It follows that

$$\begin{aligned} \xi _{[m]} = |L_r| ( m - |L_r| ) + \xi _{L_i} + \xi _{R_i} \end{aligned}$$

Using strong induction, we write $\xi _{L_r} = {|L_r| \atopwithdelims ()2}$ and $\xi _{R_r} = {m - |L_r| \atopwithdelims ()2}$. Substituting those to the formula above yields $\xi _{[m]} = {m \atopwithdelims ()2}$. That concludes the inductive step. *□ *

## Appendix B Covariance calculations

Recall that using Eq. (27) we decompose the covariance into an MSC and substitution component. Formulae for the former can be found in the Proof of Proposition 2. In this section of the Appendix, the task is to compute the latter component, namely the substitution covariance matrix for all four subtree topology cases shown in Fig. 10. This substitution component can be further decomposed as follows

$$\begin{aligned} \frac{16}{9} K \left( \Sigma _\textrm{sub} \right) _{ab,cd}&= \mathbb {E}_{G^{(1)}| \mathcal {S}} \left( e^{-\mu \left( g_{ab} + g_{cd} \right) } \left( \frac{1}{3}e^{2\mu \delta _{ab,cd}} + \frac{2}{3} e^{\mu \delta _{ab,cd}} - 1 \right) \right) \\ &= \frac{1}{3} e^{(2)}_{ab,cd} + \frac{2}{3} e^{(1)}_{ab,cd} \\ &\,\,\,\,\,\,\,\,\, -\left( \textrm{Cov}\left( e^{-\mu g_{ab}}, e^{-\mu g_{cd}}\right) + \frac{e^{- \mu (S_{ab} + S_{cd}))}}{(1+2\mu )^2 } \right) , \quad \textrm{where}\\ e^{(1)}_{ab,cd}&{:}{=}\,\mathbb {E}_{G^{(1)}| \mathcal {S}} \left( e^{-\mu (g_{ab} + g_{cd} - \delta _{ab,cd})} \right) \\ e^{(2)}_{ab,cd}&{:}{=}\,\mathbb {E}_{G^{(1)}| \mathcal {S}} \left( e^{-\mu (g_{ab} + g_{cd} - 2\delta _{ab,cd})} \right) \end{aligned}$$

where $\textrm{Cov}\left( e^{-\mu g_{ab}}, e^{-\mu g_{cd}}\right)$ is the covariance under MSC derived in Proposition 2. Therefore, the task now is to compute $e^{(1)}_{ab,cd}$ and $e^{(2)}_{ab,cd}$ for all different tree topologies.

### B.1 Computing $e^{(1)}$ terms

First, we will state all the results for $e^{(1)}_{ab,cd}$, and then provide their proofs.

- **Two-leaves.** If $\{a,b\} = \{c,d\}$, then $\delta _{ab,ab} = g_{ab}$ and so
  $$\begin{aligned} e^{(1)}_{ab,ab} = \frac{e^{-\mu S_{ab} }}{ 1+2\mu }. \end{aligned}$$
- **Three-leaf tree** with tree topology ((*a*, *b*), *c*) has
  $$\begin{aligned} e^{(1)}_{ab,ac} = e^{(1)}_{ac,bc} = e^{(1)}_{ab,bc}= \frac{e^{-\mu \left( \Delta + \frac{S_{ab}}{2} \right) } }{(1+\mu ) (1+2\mu )} \end{aligned}$$
- **Four leaves (Cherry tree)** with tree topology ((*a*, *b*), (*c*, *d*)) has
  $$\begin{aligned} e^{(1)}_{ab,cd}&= \frac{e^{-\mu ( S_{ab} + S_{cd}) }}{(1+2\mu )^2} + e^{-(2\mu +1) \Delta + \frac{S_{ab} + S_{cd}}{2}} \frac{2\mu }{(\mu + 1)(2\mu + 1)^2(2\mu + 3)} \\ e^{(1)}_{ac,bd} = e^{(1)}_{ad,bc}&= \frac{e^{-\mu \left( \Delta + \frac{S_{ab} + S_{cd}}{2} \right) }}{(1+2\mu )(1+\mu )^2} + e^{-(2\mu +1) \Delta + \frac{S_{ab} + S_{cd}}{2}} \frac{\mu (\mu + 2)}{(\mu + 1)^2(2\mu + 1)(2\mu + 3)} \end{aligned}$$
- **Four leaves (Comb tree)** with tree topology (((*a*, *b*), *c*), *d*) has
  $$\begin{aligned} e^{(1)}_{ab,cd}&= \frac{e^{-\mu (S_{ab} + \Delta )}}{(1+2\mu )^2} + e^{-\mu (\Delta + S_{ac}) - \frac{S_{ac} - S_{ab}}{2}} \frac{2\mu }{(\mu + 1)(2\mu + 1)^2(2\mu + 3)} \\ e^{(1)}_{ac,bd} = e^{(1)}_{ad,bc}&= \frac{e^{-\mu \left( \Delta + \frac{S_{ab} + S_{ac}}{2} \right) }}{(1+2\mu )(1+\mu )^2} + e^{-\mu (\Delta + S_{ac}) - \frac{S_{ac} - S_{ab}}{2}} \frac{\mu (\mu + 2)}{(\mu + 1)^2(2\mu + 1)(2\mu + 3)} \end{aligned}$$

Here and for the remainder of the Appendix, *Δ * is the diameter of the subtree with leaves *a*, *b*, *c*, *d* and should not be confused with the diameter of the total tree, which may include more leaves and thus be larger. A more accurate notation would be $\Delta _{abcd}$, but we will write *Δ * for convenience.

For the **2-leaf** case, note that $\delta _{ab,ab} = g_{ab}$ and so

$$ e^{(1)}_{ab,ab} = \mathbb {E}(e^{-\mu g_{ab}}) = e^{\mu S_{ab}} \mathbb {E}\left( e^{-2\mu E_1} \right) = \frac{ e^{\mu S_{ab}}}{1+2\mu }, $$

where $E_1 {:}{=}(g_{ab} - S_{ab})/2 \sim {{\,\textrm{Exp}\,}}(1)$ under the MSC, whose moment generating function is *1/(1-t)* which gives the *1/(1+2μ )* term in the expression above.

For the **3-leaf** case, note that

$$ \Lambda {:}{=}g_{ab} + g_{ac} - \delta _{ab,ac} = g_{ab} + g_{bc} - \delta _{ab,bc} = g_{ac} + g_{bc} - \delta _{ac,bc}, $$

which is equal to the sum of the branches of the gene tree containing the three leaves $\{a,b,c\}$, regardless of the topology of the gene tree. Let *T* be the first coalescent time in the gene tree i.e. $T = \frac{1}{2} \min \{ g_{ab}, g_{ac}, g_{bc}\}$. Then, the total branch length of the gene tree generated by $\{a,b,c\}$ is

$$\begin{aligned} \Lambda = {\left\{ \begin{array}{ll} T+\Delta + 2E_1, & \textrm{if} \,\, T \in \left( \frac{S_{ab}}{2}, \frac{\Delta }{2} \right) \\ 3T + 2E_1, & \textrm{if} \,\, T \ge \frac{\Delta }{2} \, \end{array}\right. } \end{aligned}$$

where $E_1 \sim {{\,\textrm{Exp}\,}}(1)$ is independent of *T*. Figure 12 illustrates this result.

The pdf of *T* is

$$\begin{aligned} f_{T}(T) = {\left\{ \begin{array}{ll} \exp \left( -\left( T-\frac{S_{ab}}{2}\right) \right) , & \textrm{if} \,\, T \in \left( \frac{S_{ab}}{2}, \frac{\Delta }{2} \right) \\ 3 \exp \left( - \frac{\Delta - S_{ab} }{2} - 3 \left( T-\frac{\Delta }{2} \right) \right) , & \textrm{if} \,\, T \ge \frac{\Delta }{2} \\ 0, & \textrm{otherwise} \end{array}\right. } \end{aligned}$$

Therefore, we have that

B34 $$\begin{aligned} e^{(1)}_{ab,ac} = e^{(1)}_{ac,bc} = e^{(1)}_{ab,bc}&= \mathbb {E}_T\left( \mathbb {E}(e^{t\Lambda }|T)\right) \nonumber \\ &= \frac{1}{1+2\mu } \left[ \int _{\frac{S_{ab}}{2}}^{ \frac{\Delta }{2} } \exp \left( -\left( T-\frac{S_{ab}}{2}\right) - \mu T - \Delta \right) \, dT \right. \nonumber \\ &\left. \quad + \int _{ \frac{\Delta }{2} }^{\infty } 3 \exp \left( - \frac{\Delta - S_{ab} }{2} - 3 \left( T-\frac{\Delta }{2} \right) -3\mu T\right) \, dT \right] \nonumber \\ &= \frac{e^{-\mu \left( \Delta + \frac{S_{ab}}{2} \right) } }{(1+\mu ) (1+2\mu )}, \end{aligned}$$

as stated for the **3-leaf tree**.

For the **4-leaf cherry tree** with topology ((*a*, *b*), (*c*, *d*)), again denote *T* as the first coalescence time and without loss of generality let $S_{ab} \le S_{cd}$. We also define the random variable $\Lambda _{0}$ as the sum of branch lengths of a 3-leaf gene tree, whose species tree is the star tree with zero diameter, and whose MGF is a special case of Eq. (B34) with $\Delta = S_{ab} = 0$, i.e.

B35 $$\begin{aligned} \mathbb {E}\left( e^{-\mu \Lambda _{0}}\right) = \frac{1}{(1+2\mu )(1+\mu )}. \end{aligned}$$

The central idea of this derivation is that after 2 leaves have coalesced into one, the problem is reduced to a 3-leaf case that is already computed. Note that if $T< \frac{\Delta }{2}$, then either the first pair to coalesce is either (*a*, *b*) or (*c*, *d*). Either way, $\delta _{ab,cd} = 0$. Conditioning on *T* and the corresponding first coalescent pair $\textbf{p}$,

$$\begin{aligned} g_{ab} + g_{cd} - \delta _{ab,cd}|(T,\textbf{p}) \overset{d}{=} {\left\{ \begin{array}{ll} 2T + \Delta + 2 E_1, & \textrm{if} \,\, T \in \left( \frac{S_{ab}}{2}, \frac{\Delta }{2} \right) \\ 4T + 2 E_1,& \textrm{if} \,\, T> \frac{\Delta }{2}, \textbf{p} = (a,b) \text { or } (c,d)\\ 4T + \Lambda _{0}, & \textrm{if} \,\, T > \frac{\Delta }{2}, \textbf{p} \ne (a,b) \text { or } (c,d) \end{array}\right. } \end{aligned}$$

where $E_1 \sim {{\,\textrm{Exp}\,}}(1)$ and $\Lambda _0$ are independent of *T*. It follows that,

$$\begin{aligned} e^{(1)}_{ab,cd}&= \mathbb {E}\left( e^{-\mu (g_{ab} + g_{cd} - \delta _{ab,cd}) } \right) \\ &= \int _{\frac{S_{ab}}{2}}^{\frac{\Delta }{2}} \frac{\exp \left( -\left( T-\frac{S_{ab}}{2}\right) -\mu (2T + \Delta ) \right) }{1+2\mu } \, dT \\ &\quad + e^{-\frac{\Delta -S_{ab}}{2} } \int _{\frac{S_{cd}}{2}}^{\frac{\Delta }{2}} \frac{\exp \left( -\left( T-\frac{S_{cd}}{2}\right) -\mu (2T + \Delta ) \right) }{1+2\mu } \, dT \\ &\quad + e^{-\left( \Delta - \frac{S_{ab} + S_{cd}}{2} \right) } \left( \int _{\frac{\Delta }{2}}^{\infty } \frac{6\exp \left( -6(T-\Delta ) - 4 \mu T) \right) }{1+2\mu }\, dT \cdot \mathbb {P}\left( \textbf{p}= (a,b),(c,d)\right) + \right. \\ &\quad + \left. \int _{\frac{\Delta }{2}}^{\infty } 6\exp \left( -6(T-\Delta ) - 4 \mu T) \right) \mathbb {E}\left( e^{-\mu \Lambda _{0}}\right) \, dT \cdot \mathbb {P}\left( \textbf{p}\ne (a,b),(c,d) \right) \right) \end{aligned}$$

which yields the desired result, once we substitute Eq. (B35) and note that $\mathbb {P}(\textbf{p} = (a,b),(c,d) ) =2/6 = 1/3$. Similarly, note that

$$\begin{aligned} g_{ac} + g_{bd} - \delta _{ac,bd}|(T,\textbf{p}) \overset{d}{=} {\left\{ \begin{array}{ll} 4T + \Lambda (\Delta -2T,S_{cd}-2T), & \textrm{if} \,\, T \in \left( \frac{S_{ab}}{2}, \frac{S_{cd}}{2} \right) , \textbf{p} = (a,b)\\ 4T + \Lambda (\Delta -2T,0), & \textrm{if} \,\, T \in \left( \frac{S_{cd}}{2}, \frac{\Delta }{2} \right) , \textbf{p} = (a,b), (c,d)\\ 4T + 2 E_1,& \textrm{if} \,\, T> \frac{\Delta }{2}, \textbf{p} = (a,c) \text { or } (b,d)\\ 4T + \Lambda _{0}, & \textrm{if} \,\, T > \frac{\Delta }{2}, \textbf{p} \ne (a,c) \text { or } (b,d) \end{array}\right. } \end{aligned}$$

where $E_1 \sim {{\,\textrm{Exp}\,}}(1)$ is independent of *T*. It follows that

$$\begin{aligned} e^{(1)}_{ac,bd}&= \mathbb {E}\left( e^{-\mu (g_{ac} + g_{bd} - \delta _{ac,bd}) } \right) \\ &= \int _{\frac{S_{ab}}{2}}^{\frac{S_{cd}}{2}} \exp \left( -\left( T-\frac{S_{ab}}{2}\right) -4\mu T) \right) \frac{\exp \left( -\mu \left( \Delta + \frac{S_{ab}}{2} \right) +3\mu T \right) }{(1+\mu )(1+2\mu )} \, dT \\ &\quad + e^{-\frac{S_{cd}-S_{ab}}{2} } \int _{\frac{S_{cd}}{2}}^{\frac{\Delta }{2}} 2\exp \left( -2\left( T-\frac{S_{cd}}{2}\right) -4\mu T) \right) \frac{\exp \left( -\mu \Delta + 2\mu T \right) }{(1+\mu )(1+2\mu )} \, dT \\ &\quad + e^{-\left( \Delta - \frac{S_{ab} + S_{cd}}{2} \right) } \left( \int _{\frac{\Delta }{2}}^{\infty } \frac{6\exp \left( -6(T-\Delta ) - 4 \mu T) \right) }{1+2\mu }\, dT \cdot \mathbb {P}\left( \textbf{p} = (a,c),(b,d)\right) \right. \\ &\quad + \left. \int _{\frac{\Delta }{2}}^{\infty } 6\exp \left( -6(T-\Delta ) - 4 \mu T) \right) \frac{dT}{(1+\mu )(1+2\mu )} \cdot \mathbb {P}\left( \textbf{p} \ne (a,c),(b,d)\right) \right) \\ &= \frac{e^{-\mu \left( \Delta + \frac{S_{ab} + S_{ac}}{2} \right) }}{(1+2\mu )(1+\mu )^2} + e^{-\mu (\Delta + S_{ac}) + \frac{\Delta - S_{ab}}{2}} \frac{\mu (\mu + 2)}{(\mu + 1)^2(2\mu + 1)(2\mu + 3)} \end{aligned}$$

Note that since *a* and *b* are interchangeable in this tree topology, $e^{(1)}_{ad,bc} = e^{(1)}_{ac,bd}$. This concludes the proof for the cherry tree case.

Finally, for the **4-leaf comb tree** case with topology (((*a*, *b*), *c*), *d*), and noting that

$$\begin{aligned} g_{ab} + g_{cd} - \delta _{ab,cd}|(T,\textbf{p}) \overset{d}{=} {\left\{ \begin{array}{ll} 2T + \Delta + 2 E_1, & \textrm{if} \,\, T \in \left( \frac{S_{ab}}{2}, \frac{S_{ac}}{2} \right) \\ 2T + \Delta + 2 E_1, & \textrm{if} \,\, T \in \left( \frac{S_{ac}}{2}, \frac{\Delta }{2} \right) , \textbf{p} = (a,b)\\ 4T + \Lambda (\Delta -2T,0), & \textrm{if} \,\, T \in \left( \frac{S_{ac}}{2}, \frac{\Delta }{2} \right) , \textbf{p} = (a,c),(b,c)\\ 4T + 2 E_1,& \textrm{if} \,\, T> \frac{\Delta }{2}, \textbf{p} = (a,c) \text { or } (b,d)\\ 4T + \Lambda _{0}, & \textrm{if} \,\, T > \frac{\Delta }{2}, \textbf{p} \ne (a,c) \text { or } (b,d) \end{array}\right. } \end{aligned}$$

$$\begin{aligned} g_{ac} + g_{bd} - \delta _{ac,bd}|(T,\textbf{p}) \overset{d}{=} {\left\{ \begin{array}{ll} 4T + \Lambda (\Delta -2T,S_{ac}-2T), & \textrm{if} \,\, T \in \left( \frac{S_{ab}}{2}, \frac{S_{ac}}{2} \right) \\ 2T + \Delta + 2 {{\,\textrm{Exp}\,}}(1), & \textrm{if} \,\, T \in \left( \frac{S_{ac}}{2}, \frac{\Delta }{2} \right) , \textbf{p} = (a,c)\\ 4T + \Lambda (\Delta -2T,0), & \textrm{if} \,\, T \in \left( \frac{S_{ac}}{2}, \frac{\Delta }{2} \right) , \textbf{p} = (a,b),(b,c)\\ 4T + 2 {{\,\textrm{Exp}\,}}(1),& \textrm{if} \,\, T> \frac{\Delta }{2}, \textbf{p} = (a,c) \text { or } (b,d)\\ 4T + \Lambda _{0}, & \textrm{if} \,\, T > \frac{\Delta }{2}, \textbf{p} \ne (a,c) \text { or } (b,d) \end{array}\right. } \end{aligned}$$

where $E_1 \sim {{\,\textrm{Exp}\,}}(1)$ is independent of *T*, the desired results are derived by following the same process in the cherry tree case.

### B.2 Computing $e^{(2)}$ terms

First, we will state all the results for $e^{(2)}_{ab,cd}$, and then provide key ideas for their proof.

- **Two-leaves.** If $\{a,b\} = \{c,d\}$, then $\delta _{ab,ab} = g_{ab}$ and so the random variable in the exponent is zero, i.e.
  $$\begin{aligned} e^{(2)}_{ab,ab} = 1. \end{aligned}$$
- **Three-leaf tree** with tree topology ((*a*, *b*), *c*) has
  $$\begin{aligned} e^{(2)}_{ab,ac} = e^{(2)}_{ab,bc}&= \frac{e^{-\mu S_{ac}}}{1+2\mu } \\ e^{(2)}_{ac,bc}&= \frac{e^{-\mu S_{ab}}}{1+2\mu } \end{aligned}$$
- **Four leaves (Cherry tree)** with tree topology ((*a*, *b*), (*c*, *d*)) has
  $$\begin{aligned} e^{(2)}_{ab,cd} = e^{(2)}_{ac,bd}= e^{(2)}_{ad,bc} = \frac{e^{-\mu ( S_{ab} + S_{cd}) }}{(1+2\mu )^2} + e^{-(2\mu +1) \Delta + \frac{S_{ab} + S_{cd}}{2}} \frac{4\mu }{(2\mu + 1)^2(2\mu + 3)} \end{aligned}$$
- **Four leaves (Comb tree)** with tree topology (((*a*, *b*), *c*), *d*) has
  $$\begin{aligned} e^{(2)}_{ab,cd} = e^{(2)}_{ac,bd}= e^{(2)}_{ad,bc} = \frac{e^{-\mu (S_{ab} + \Delta )}}{(1+2\mu )^2} + e^{-\mu (\Delta + S_{ac}) - \frac{S_{ac} - S_{ab}}{2}} \frac{4\mu }{(2\mu + 1)^2(2\mu + 3)} \end{aligned}$$

To prove the three leaf case, observe that

B36 $$\begin{aligned} g_{xy} + g_{xz} - 2 \delta _{xy,xz} = g_{yz}, \quad \forall (x,y,z) \in \left( {\begin{array}{c}L\\ 3\end{array}}\right) \end{aligned}$$

In other words,

$$\begin{aligned} e^{(2)}_{xy,xz} = \mathbb {E}(e^{-\mu g_{yz}}) = \frac{e^{-\mu S_{yz}}}{1+2\mu } \end{aligned}$$

For the four-leaf comb and cherry case, we observe that

$$\begin{aligned} g_{ab} + g_{cd} - 2\delta _{ab,cd} = g_\textbf{p} + g_\mathbf{p^c}, \end{aligned}$$

where $$\textbf{p} \in \left( {\begin{array}{c}\{a,b,c,d\}\\ 2\end{array}}\right) $$ is the first pair in the gene tree to coalesce and $\textbf{p}^c$ is the complimentary pair; the remaining two leaves. Conditioning on $T,\textbf{p}$ just like before yields the desired results.

## Appendix C Algorithms

Algorithm 1 describes the procedure to compute the Hamming distance between two alignment sequences. This is a sub-routine within the distance-based approaches for species tree clustering - METAL, GLASS and STEAC - which are presented in Algorithm 2.

## Appendix D Additional plots

See Figs. 14, 15, 16, and 17.
